# Genetic association and transcriptome integration identify contributing genes and tissues at cystic fibrosis modifier loci

**DOI:** 10.1371/journal.pgen.1008007

**Published:** 2019-02-26

**Authors:** Jiafen Gong, Fan Wang, Bowei Xiao, Naim Panjwani, Fan Lin, Katherine Keenan, Julie Avolio, Mohsen Esmaeili, Lin Zhang, Gengming He, David Soave, Scott Mastromatteo, Zeynep Baskurt, Sangook Kim, Wanda K. O’Neal, Deepika Polineni, Scott M. Blackman, Harriet Corvol, Garry R. Cutting, Mitchell Drumm, Michael R. Knowles, Johanna M. Rommens, Lei Sun, Lisa J. Strug

**Affiliations:** 1 Program in Genetics and Genome Biology, The Hospital for Sick Children, Toronto, ON, Canada; 2 Department of Statistical Sciences, University of Toronto, Toronto, ON, Canada; 3 Program in Physiology and Experimental Medicine, The Hospital for Sick Children, Toronto, ON, Canada; 4 Program in Translational Medicine, The Hospital for Sick Children, Toronto, ON, Canada; 5 Biostatistics Division, Dalla Lana School of Public Health, University of Toronto, Toronto, ON, Canada; 6 Wilfrid Laurier University, Department of Mathematics, Waterloo, Ontario, Canada; 7 Ontario Institute for Cancer Research, Department of Computational Biology, Toronto, Ontario, Canada; 8 Marsico Lung Institute and Cystic Fibrosis Pulmonary Research and Treatment Center, University of North Carolina, Chapel Hill, North Carolina, United States of America; 9 Department of Internal Medicine, University of Kansas Medical Centre, Kansas City, Kansas, United States of America; 10 Department of Pediatrics, Johns Hopkins University School of Medicine, Baltimore, Maryland, United States of America; 11 Assistance Publique-Hôpitaux de Paris (AP-HP), Hôspital Trousseau, Pediatric Pulmonary Department; Institut National de la Santé et la Recherche Médicale (INSERM) U938, Paris, France; 12 Sorbonne Universités, Université Pierre et Marie (UPMC) Paris, Paris, France; 13 McKusick-Nathans Institute of Genetic Medicine, Johns Hopkins University School of Medicine, Baltimore, Maryland, United States of America; 14 Department of Pediatrics, Case Western Reserve University, Cleveland, Ohio, United States of America; 15 Department of Genetics, Case Western Reserve University, Cleveland, Ohio, United States of America; 16 Department of Molecular Genetics, University of Toronto, Toronto, ON, Canada; 17 The Centre for Applied Genomics, The Hospital for Sick Children, Toronto, ON, Canada; Newcastle University, UNITED KINGDOM

## Abstract

Cystic Fibrosis (CF) exhibits morbidity in several organs, including progressive lung disease in all patients and intestinal obstruction at birth (meconium ileus) in ~15%. Individuals with the same causal *CFTR* mutations show variable disease presentation which is partly attributed to modifier genes. With >6,500 participants from the International CF Gene Modifier Consortium, genome-wide association investigation identified a new modifier locus for meconium ileus encompassing *ATP12A* on chromosome 13 (min p = 3.83x10^-10^); replicated loci encompassing *SLC6A14* on chromosome X and *SLC26A9* on chromosome 1, (min p<2.2x10^-16^, 2.81x10^−11^, respectively); and replicated a suggestive locus on chromosome 7 near *PRSS1* (min p = 2.55x10^-7^). *PRSS1* is exclusively expressed in the exocrine pancreas and was previously associated with non-CF pancreatitis with functional characterization demonstrating impact on PRSS1 gene expression. We thus asked whether the other meconium ileus modifier loci impact gene expression and in which organ. We developed and applied a colocalization framework called the *Simple Sum (SS)* that integrates regulatory and genetic association information, and also contrasts colocalization evidence across tissues or genes. The associated modifier loci colocalized with expression quantitative trait loci (eQTLs) for *ATP12A* (p = 3.35x10^-8^), *SLC6A14* (p = 1.12x10^-10^) and *SLC26A9* (p = 4.48x10^-5^) in the pancreas, even though meconium ileus manifests in the intestine. The meconium ileus susceptibility locus on chromosome X appeared shifted in location from a previously identified locus for CF lung disease severity. Using the *SS* we integrated the lung disease association locus with eQTLs from nasal epithelia of 63 CF participants and demonstrated evidence of colocalization with airway-specific regulation of *SLC6A14* (p = 2.3x10^-4^). Cystic Fibrosis is realizing the promise of personalized medicine, and identification of the contributing organ and understanding of tissue specificity for a gene modifier is essential for the next phase of personalizing therapeutic strategies.

## Author summary

Cystic Fibrosis (CF) impacts the normal functioning of several organs including the pancreas, intestines and lungs. CF is caused by mutations in the CF transmembrane conductance regulator, but individuals with the same mutations have different disease severity. For example, only ~15% of individuals with CF are born with intestinal obstruction at birth (meconium ileus) while all have progressive lung disease with varying severity. Modifier genes contribute to variation across individuals with CF. In this study we identified modifiers of meconium ileus susceptibility, encompassing the previously identified *SLC26A9* and *SLC6A14*, and two new loci *ATP12A* and *PRSS1*. To understand the mechanism by which these modifier genes impact CF severity we developed a new statistical method that assesses colocalization with regulatory information. Using this methodology we show, surprisingly, that all the meconium ileus association signals colocalize with gene expression in the *pancreas* rather than in intestinal tissues. A distinct and neighbouring locus near *SLC6A14* also contributes to CF lung disease. Aided by transcriptomics of nasal epithelia from CF patients we found that each locus impacts variation in gene expression of *SLC6A14* with tissue specificity. Understanding the contributing tissue and responsible gene are necessary to prioritize modifiers as alternative therapeutic targets.

## Introduction

Greater than 348 CF transmembrane conductance regulator *(CFTR)* gene variants are known to cause CF, where epithelial function is impaired in several organs including the lung, pancreas, and intestine, amongst others (see CFTR2 (Web Resources in Material and Methods) and [1]). Individuals with the same causal *CFTR* genotype display a wide range of disease burden within and across organs and exhibit variable response to costly *CFTR* mutation-directed therapies. Modifier gene relationships with CFTR may involve tissue-specific facets that explain some of this variability, and need to be understood to improve therapeutic strategies [2].

Significant morbidity and mortality in CF is a consequence of progressive lung disease, for which the estimated heritability is approximately 50% [3] and correlates poorly with *CFTR* genotype [4]. Meconium ileus, intestinal obstruction due to failure to pass the meconium, occurs in 13–21% [5] of CF newborns, with equal frequency in males and females. Meconium ileus displays heritability estimates of ~88% [6] and can be discordant for CF siblings with identical *CFTR* mutations, further highlighting the importance of genetic background beyond *CFTR*. Meconium, or first stool of a newborn, is typically comprised of intestinal mucins, proteins, bile salts, and cellular debris that are shed from the intestinal mucosa during the fetal period [7]. The meconium exhibits increased thickness and adherent properties in CF, with blockage. Evidence for blockage or meconium ileus can be seen on ultrasound as early as the second trimester, and although there can be resolution *in utero*, persistence to beyond birth is fatal without enema or surgical intervention [7]. Given the early occurrence, unambiguous presentation and high heritability, genes that contribute to meconium ileus may be more amenable to gene mapping. Moreover, genetic contributors to meconium ileus have been shown to inform disease severity in other CF affected organs.

Genome-wide association studies (GWAS) have identified modifier genes that contribute to morbidity across multiple CF-affected organs [8], with the associated variants frequently annotated to putative regulatory regions of nearby genes. For example, allelic variation near the transcription start site (TSS) of *SLC26A9*, which encodes an anion transporter in epithelial cells that interacts with CFTR to enhance its functional expression [9–11], has been shown to associate with aspects of CF lung disease [2], meconium ileus [12], CF-related diabetes (CFRD) [13], as well as exocrine pancreatic damage at birth [14, 15]. Although the same modifier can show association with multiple CF co-morbidities, it is unclear whether these are independent effects as their timing of impact and expression levels vary. Exemplary is how *SLC26A9* appears to contribute to CFRD through exocrine pancreatic damage in utero [15], an example of vertical pleiotropy [16].

Here, we are interested in understanding the relation between the intestine, where meconium develops, and the pancreas. The presence of meconium ileus is correlated with disease in other CF affected organs, in particular pancreatic disease. Direct pancreatic function studies have shown that individuals with CF have reduced digestive enzyme secretory fluid with low pH and high protein concentrations [17, 18]. Meconium ileus occurs almost exclusively in individuals with severe *CFTR* mutations known to confer pancreatic exocrine insufficiency [5, 19] (~90% of the CF population [20]), therefore modifier gene studies of meconium ileus have been restricted to this CF sub-population. It has been noted that those with meconium ileus on average have relatively less elevated immunoreactive trypsinogen (IRT) levels at birth compared to patients without meconium ileus ([15, 21]); elevated IRT is the newborn screening biomarker for CF and is also reflective of the extent of prenatal pancreatic injury [19, 22]. The phenotypic correlation between meconium ileus and CF pancreatic disease, including CFRD [13], leads one to consider the involvement of the pancreas in the development of meconium ileus. Evidence that gene expression in the pancreas is mediating the association between meconium ileus and the GWAS variants would support this hypothesis.

Using the largest study population of individuals with CF to date from the International CF Gene Modifier Consortium (*GMC*), we (1) identify genetic loci contributing to meconium ileus using a combination of whole genome genotype arrays and next generation sequencing; (2) integrate publicly available cross-tissue gene expression [23] (and epigenomic data [24–27]) with genotypes (Material and Methods and S1 Appendix) to test the hypothesis that the variants associated with meconium ileus are colocalizing with variants that impact gene expression and (3) determine the most probable originating tissue for the meconium ileus modifiers. We also (4) integrate RNA-sequencing of nasal epithelia from individuals with CF to determine cross-phenotypic mechanisms and tissue-specific GWAS signals.

## Results

Participants include n = 6,770 individuals with CF from Canada, the United States and France, recruited in two phases by the GMC (Table 1), and genotyped on various Illumina platforms (Material and Methods, S1 and S2 Tables). With the incorporation of a hybrid reference [28] and after standard quality control (Material and Methods), a total of 8,763,019 (genotyped and imputed) variants were analyzed for association with meconium ileus (with clinical definitions as previously described; [12]).

**Table 1:** Characteristics of CF participants included in the genome-wide study of meconium ileus from the International CF Gene Modifier Consortium (GMC). The four consortium sites are Canadian CF Gene Modifier Study (CGMS), UNC/Case Western Reserve Modifier Study (UNC/Case), Johns Hopkins University CF Twin and Sibling Study (JHU), and French Gene Modifier Study (FGMS). Additional sample details stratified by genotyping platform are provided in S1 Table.

Consortium Site	Participants^a^			Female %	*CFTR* genotype		meconium ileus		
					Phe508del/	Phe508del/	Yes	No	%F^b^ with MI (p-value^c^)
					Phe508del	other			
	Phase I-NA	Phase II-NA	Mega-all		%	%			
CGMS	1519	338	1857	46.3	61.1	31.3	310	1547	53.2 (0.2805)
UNC/Case	1303	602	1905	45.7	78.4	19.1	326	1579	46.6 (0.2448)
JHU	1042	700	1742	47.4	56.2	37.9	320	1422	47.2 (0.3419)
FGMS	-	-	1266	48.7	58.1	35.8	159	1107	54.7 (0.2669)
Total	3864	1640	6770	46.8	64.2	30.4	1115	5655	49.8 (0.9046)

^a^All patients from the four study sites had two severe CFTR mutations associated with pancreatic insufficiency, and all patients clustered within 6 S.D. of the Hapmap (v3) [29] Utah residents with Northern and Western European ancestry from the CEPH collection (CEU) and Toscani in Italia (TSI) in a principal component analysis (PCA).

^b^%F indicates percentage of female participants.

^c^p-value tests whether the proportion of females differ from 0.5

### Genome-wide association analysis

**Fig 1:**
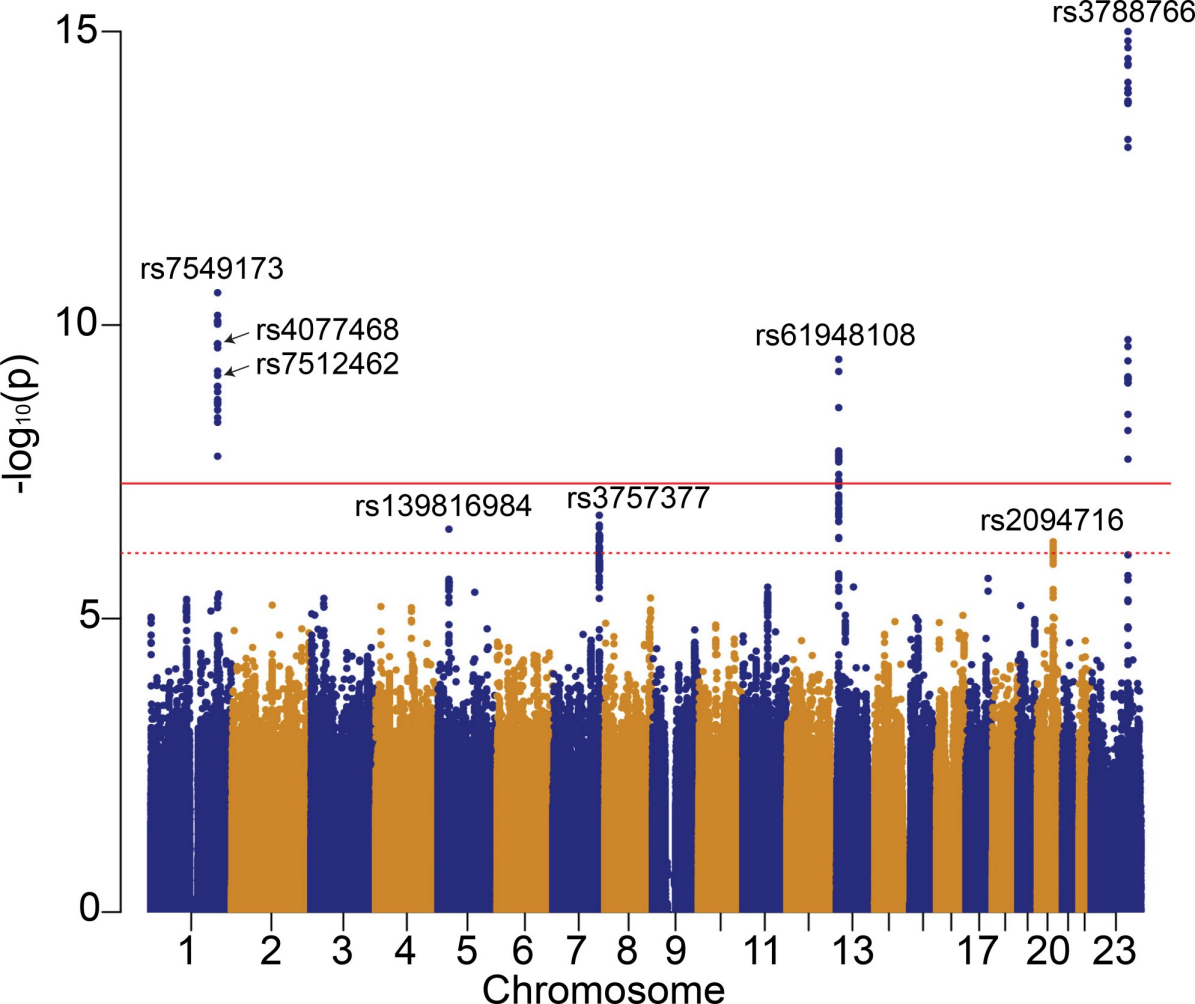
Manhattan plot for the genome-wide association study with meconium ileus in the GMC sample of 6,770 individuals with CF. All variants with MAF>1% are included in the analysis. The solid horizontal line corresponds to the genome-wide significance threshold of p = 5x10^-8^ [30], and the dotted horizontal line is the false discovery rate control threshold of q = 0.05 (equivalent to p<7.67x10^-7^) [31]_._ Accuracy of p-value calculation was up to 2.2x10^-16^ by the geeglm function [100] in R. Variants rs4077468 and rs7512462 identified in previous CF gene modifier studies also noted.

Genome-wide association analysis used generalized estimating equations with an exchangeable correlation structure (to account for siblings present in the data), and included adjustment for consortium site, genotype platform and population stratification (S1 Fig for the first three principal components). Three genome-wide significant loci with nearest genes *SLC6A14*, *SLC26A9* and *ATP12A* (p-values<5x10^-8^ based on the conventional family-wise error control [30]), and three additional loci with nearest genes *PRSS1*, *TARS*, *CEBPB* (q-values<0.05 based on the false discovery rate control [31]) were evident (Fig 1).

**Fig 2:**
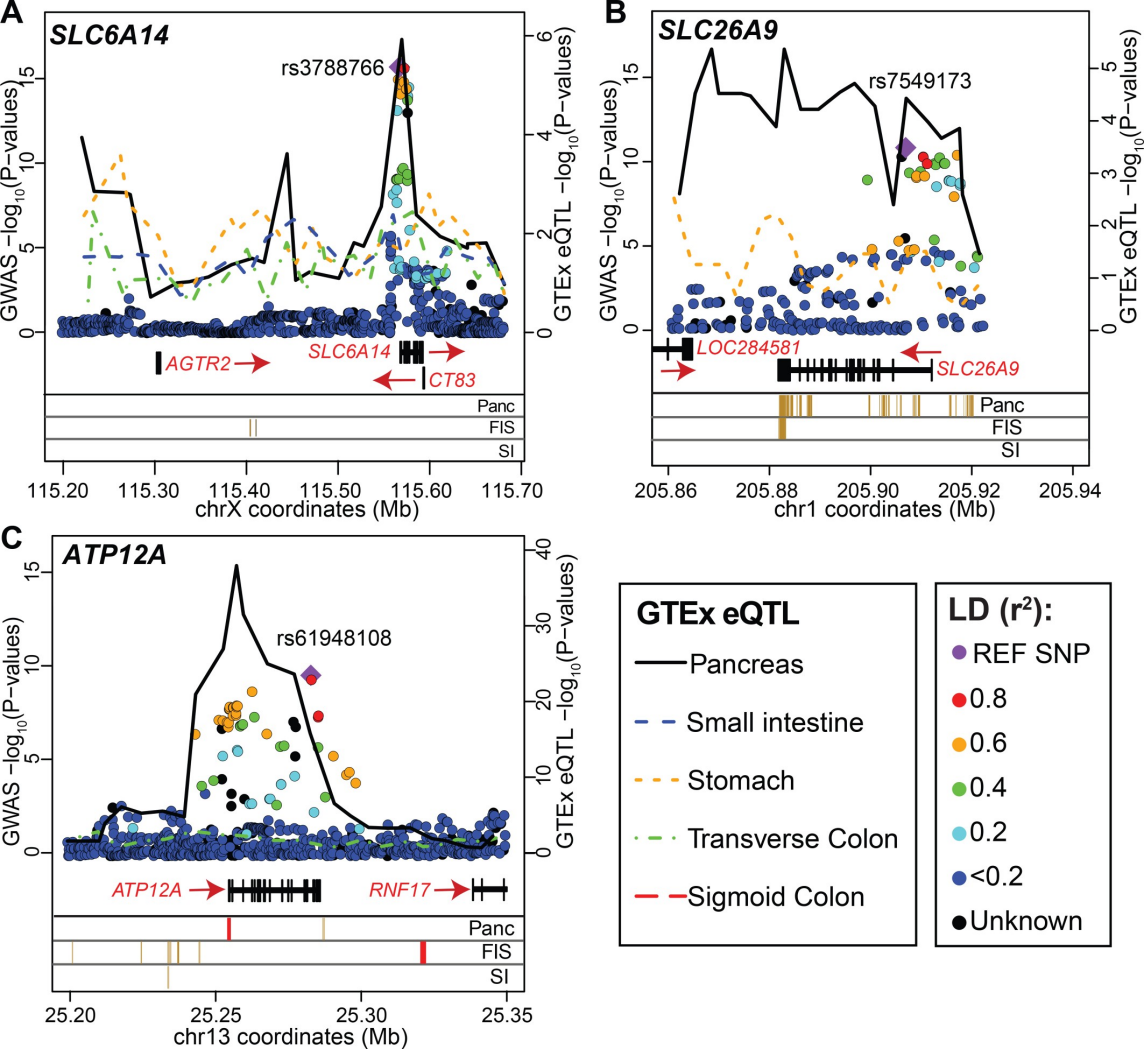
Overlay of the p-values of individual SNPs from the meconium ileus GWAS and GTEx eQTL association and the enhancer and promoter states from the REMC at the three genome-wide significant loci. All the p-values are on the–log_10_ scale with the left y-axis representing the GWAS (with the filled diamond for the SNP with the lowest p-value and colored dots indicating the LD with this SNP) and the right y-axis representing the GTEx eQTL results (version 7; with colored lines indicated for different tissues) for *SLC6A14* (A), *SLC26A9* (B), and *ATP12A* (C). Note that eQTLs for *SLC6A14* in the pancreas and *ATP12A* in the transverse colon were not mapped by GTEx v7, but were calculated and included here—refer to Material and Methods, GTEx Data section for details. To display the eQTL data, each physical region shown was divided into 25 equal windows and lines were drawn for each tissue by connecting the minimum GTEx eQTL p-value within each window for that gene. The absence of a line indicates insufficient gene expression for that particular tissue (see GTEx Data section for details on expression threshold). In the lower panels, the 5-mark 15-state observed chromHMM enhancer (orange) and promoter (red) states within DNase hypersensitive sites are shown (Material and Methods) from adult pancreas (Panc), fetal small intestine (FIS) and adult small intestine (SI).

The chromosome X (*SLC6A14*; panel (a) of Fig 2) and chromosome 1 (*SLC26A9*; panel (b) of Fig 2) loci were identified as genome-wide significant in an earlier meconium ileus study by the GMC [12] using a subset of the current study sample (S3 Table); this earlier study also demonstrated that a set of 157 genes that code for constituents of the apical plasma membrane where CFTR resides (see Supplementary Table 3 in [12]) were significantly enriched for variants associated with meconium ileus, and this gene-set association was replicated in the French Gene Modifier Study (FGMS) cohort [12, 32]. The expanded sample (Table 1 and S1 Table) analyzed here provides further support for the same variants at the *SLC6A14* and *SLC26A9* loci (Table 2, minimum p<2.2x10^-16^ and p = 2.81x10^-11^ in column ‘Mega-all’, and S3 Table). Support for *CFTR* (min p = 2.34x10^-5^ at rs213972; gene-based permutation p = 0.0001; S2 Fig) and the association between the apical plasma membrane gene-set and meconium ileus was also observed in this study (permutation p<1x10^-4^ in a subset of 5,869 unrelated individuals from the full consortium data and p = 0.017 in the North American Phase II subgroup alone). The confirmation of multiple contributing genes supports the existence of genetic heterogeneity, indicating that meconium ileus is a phenotype of complex genetic etiology within a mendelian disease.

**Table 2:** Top ranked variants by p-value from each genome-wide significant locus in the GMC sample of 6,770 individuals with CF. The first three rows correspond to loci with SNPs that reached genome-wide significance and the bottom three rows correspond to loci with SNPs that exceeded false discovery rate (FDR) control threshold of q = 0.05 (equivalent to p<7.67x10^-7^). The top SNP in the *SLC6A14* region remains the same at rs3788766 as in [12], while the top SNP at the *SLC26A9* locus is now rs7549173. However all significant loci reported at the *SLC26A9* locus in [12] are in linkage disequilibrium with rs7549173 (S3 Table), including rs7512462 which has been used as an instrumental variable in a Mendelian Randomization analysis of CF-related diabetes and shown to associate with exocrine pancreas disease [15] and CFTR-directed therapeutics response [2]. The variants in the *ATP12A* and *PRSS1* regions in the previous study [12] are provided in S3 Table. The chromosome 5 and 20 variants are new findings and were not previously identified in [12].

Chr	BP	SNPs	Nearest gene	Risk Allele	^1^Mega-all		^2^Mega-DFDF		^3^Phase I-NA		^4^Phase II-NA		^5^FGMS	
					OR	P	OR	P	OR	P	OR	P	OR	P
X	115566839	rs3788766	*SLC6A14*	A	1.44	<2.2x10^-16^	1.40	2.21x10^−11^	1.53	4.15x10^−14^	1.33	2.98x10^−4^	1.42	1.50x10^−3^
1	205906897	rs7549173	*SLC26A9*	C	1.37	2.81x10^−11^	1.41	3.37x10^−9^	1.40	8.88x10^−8^	1.37	5.80x10^−4^	1.24	0.076
13	25282819	rs61948108	*ATP12A*	T	1.55	3.83x10^-10^	1.70	5.69x10^−10^	1.54	1.42x10^−6^	1.48	4.62x10^−3^	1.78	2.50x10^−3^
7	142455538	rs3757377	*PRSS1*	T	1.29	2.55x10^-7^	1.31	5.55x10^−6^	1.34	3.88x10^−6^	1.28	8.43x10^−3^	1.12	0.4
5	33471002	rs139816984	*TARS*	AAAAAAAAT	1.35	3.01x10^-7^	1.30	1.93x10^−4^	1.33	1.85x10^−4^	1.32	0.012	1.58	6.80x10^−3^
20	48835331	rs2094716	*CEBPB*	A	1.30	4.92x10^-7^	1.28	8.90x10^−5^	1.26	1.10x10^−3^	1.29	9.30x10^−3^	1.48	3.80x10^−3^

^1^Mega-all: all GMC CF participants (n = 6,770)

^2^Mega-DFDF: individuals homozygous for the Phe508del mutation (n = 4,343)

^3^Phase I-NA: the sample (n = 3,864) analyzed previously in [12], although the current analysis differs by inclusion of additional variants available through genome-wide imputation with the hybrid reference panel

^4^Phase II-NA: continued North American recruitment (n = 1,640)

^5^FGMS: individuals from the French CF Gene Modifier Study (n = 1,266)

The *SLC6A14* association region is subject to X-inactivation based on the H3K27me3 histone methylation pattern provided by the Roadmap Epigenomics Mapping Consortium (REMC (Web Resources in Material and Methods); experiments ENCSR204NFO and ENCSR727VOB [25] in female lung tissue where strong *SLC6A14* expression occurs, GTEx [23]). Therefore, the observation that the risk alleles for the genome-wide significant SNPs display lower odds ratios (OR) in females (with two versus zero copies of the risk alleles) than the ORs in males (one versus zero copies of the risk alleles), p = 0.074, 0.046, and 0.058, respectively, for rs3768766 A, rs5905177 T, and rs12710568 C risk alleles (S4 Table), suggests the possibility of sex-specific differences beyond what is expected under random X-inactivation.

A new genome-wide significant locus was identified on chromosome 13 with minimum p-value = 3.83x10^-10^ at rs61948108 (Table 2 and S3 Table), and located in intron 17 of the ATPase H+/K+ Transporting Non-Gastric Alpha2 Subunit (*ATP12A)* (panel (c) in Fig 2). Restricting the association analysis to the 4,343 individuals homozygous for Phe508del resulted in a larger effect size (OR = 1.7 vs. 1.55, Table 2, S3 Fig). Although this did not result in statistically significant GxG interaction (interaction p = 0.057), the dependence on *CFTR* genotype of the *ATP12A*-meconium ileus relationship requires further investigation. Modifier-CFTR-mutation relationships have previously been reported for CF lung disease [2, 33], and *ATP12A* was previously implicated as a modifier of CF lung disease using candidate gene analysis in pig models [34].

Conditional analysis at each of the three genome-wide significant loci displayed no evidence for allelic heterogeneity (S4 Fig). Top variants from the three genome-wide significant loci explain 7% of the phenotypic variability for meconium ileus. Of the three FDR significant loci (Table 2), the chromosome 7 locus (‘Mega-all’ p = 2.55x10^-7^ at rs3757377 located upstream of *PRSS1*; S5 Fig) was suggestive in the previous consortium study (Fig 1 of [12]), and is replicated in the additional North American cohort (‘Phase II-NA’ p = 8.43x10^-3^, Table 2). We note that the physical landscape encompassing this locus has been revised in recent genome map versions, such that it remains unclear if variation at this locus has been fully appreciated. The new chromosomes 5 and 20 loci were not identified in the previous study [12] and will need to be investigated further.

### Integration of GWAS and tissue-specific gene expression data

The identified associated variants and those nearby in high linkage disequilibrium (LD) do not occur in gene coding regions; however, they include variants near the transcription start sites (TSS) of the nearest genes suggesting roles in gene regulation. *PRSS1*, or cationic trypsinogen, is used as a measure of early pancreatic disease in CF [35, 36]. At the *PRSS1* locus, the meconium ileus associated SNP, rs3757377 (Table 2, Chr7:142455538, 1.8kb 5’ of the TSS, OR = 1.29, p = 2.55x10^-7^; S5 Fig) is in high LD (r^2^ = 0.71 and D’ = -0.99) with rs10273639, the variant that was identified in a GWAS of alcoholic-related chronic pancreatitis in Europeans [37]. A follow-up study [38] further showed that rs4726576 (chr7:142457132, 186bp upstream of *PRSS1*; S3 Table), a variant in high LD with both GWAS study-associated variants (rs3757377 and rs10273639), is contributing to the pancreatitis relationship by directly influencing gene expression levels based on reporter gene assays in acinar pancreatic cells. These assays and consideration that the encoded digestive enzyme function originates from the pancreas argue that variants associated with meconium ileus at the *PRSS1* locus impact gene expression in this organ. The pancreatitis *protective* T allele of rs10273639 that is associated with less *PRSS1* expression is a *risk* allele for meconium ileus. The meconium of individuals with CF contain high levels of protein and less trypsinogen may adversely retain consistency [39]. We did note that no eQTLs for *PRSS1* are reported in the pancreas in the genotype tissue expression project (GTEx). This may be reflecting its extremely high expression (the *PRSS1* transcript is the most highly expressed gene in the pancreas with median TPM of 9.91 x 10^4^ [23]), or that eQTL determination is affected by the local genome landscape that has been altered in the most recent genome assembly (GRCh38/hg38) with added genes as well as indication of alternative assemblies.

To determine whether gene expression variation at the three genome-wide significant loci (*SLC6A14*, *SLC26A9* and *ATP12A*) influence meconium ileus risk and in which tissue(s), we used data from the GTEx v7 (accessed on Oct 1, 2018) [23] and considered the enhancer and promoter states from the Regulatory Regions Map (Reg2map; see Web Resources in Material and Methods) [24, 25]. We first display the association findings (Fig 2, colored circles as in the LocusZoom plots [40]) at the three loci in the context of the available eQTLs of gastro-intestinal tissues (Fig 2, colored lines indicating different tissues; eQTLs for *SLC6A14* in the pancreas and *ATP12A* in the transverse colon were included in the GTEx v6 but not v7. They were calculated using v7 data and included here; Material and Methods and sex-stratified analysis S6 Fig), with the locations of tissue-specific enhancers and promoters noted when available to complement the comparisons. Notably, the pattern of eQTL evidence for each of *SLC6A14*, *SLC26A9* and *ATP12A* in the pancreas (black solid line) mirrors the genetic association pattern (colored circles), with the enhancer and promoter states overlapping with pancreas-specific regulatory elements for at least *SLC26A9* and *ATP12A*. In contrast, eQTLs in the small intestine (blue dashed line) do not meet expression threshold criteria by either GTEx v6 or v7 (due to a very low level of expression) for *SLC26A9* and *ATP12A* (panels (b) and (c) in Fig 2) or do not overlap (*SLC6A14*, panel (a) in Fig 2) with the GWAS findings. Based on GTEx data, the meconium ileus risk alleles are associated with decreased *SLC26A9* expression, but increased *ATP12A* and *SLC6A14* expression in the pancreas.

**Fig 3:**
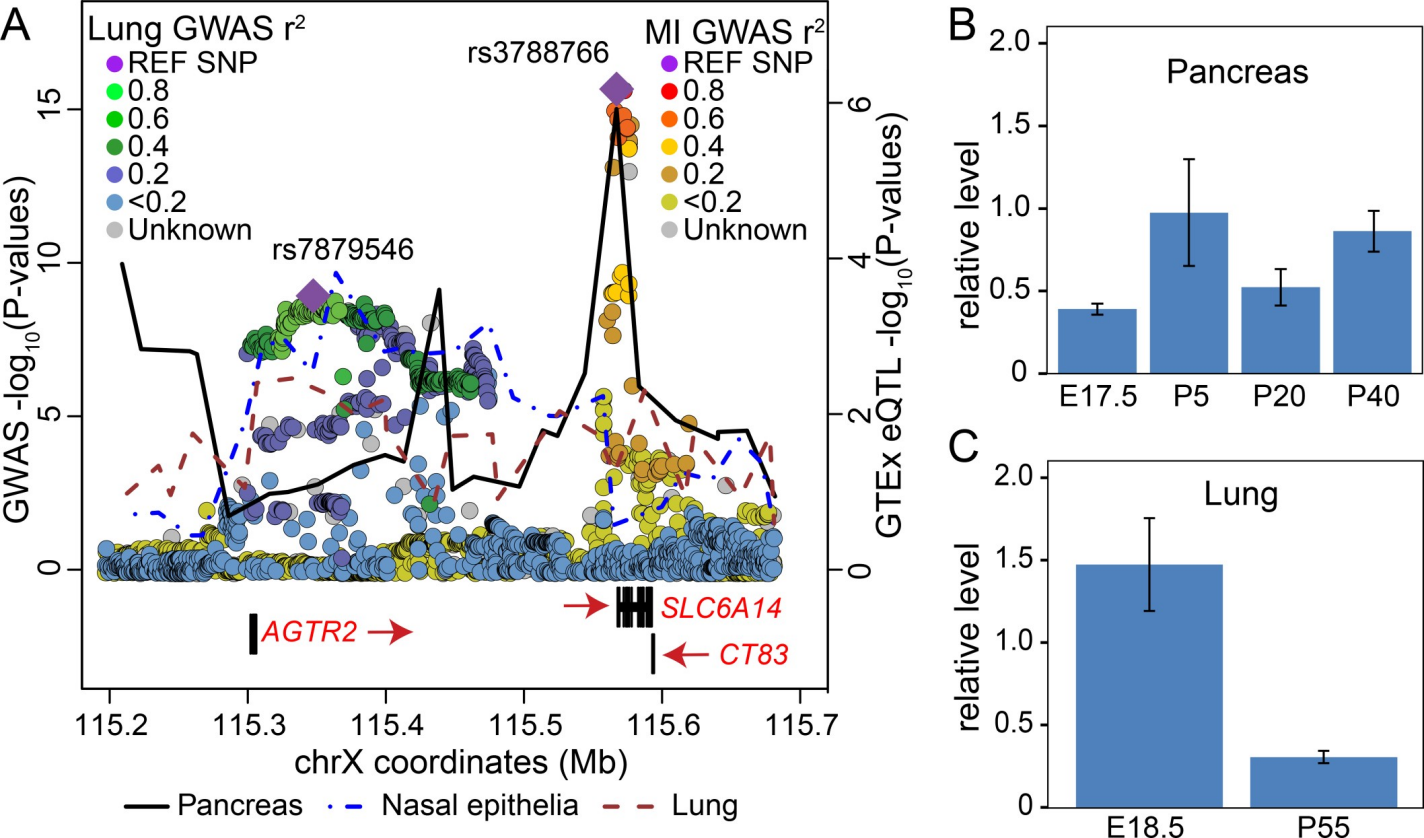
*SLC6A14* meconium ileus and lung association and gene expression profiles point to *cis*-regulation that is distinct in pancreas and lung. (A) Overlay of p-values (on the–log_10_ scale) from the meconium ileus GWAS (red/yellow palette of colored dots; this study), from the previous lung function GWAS (green/blue palette of colored dots; [41]) and from the GTEx eQTL for association between SNPs and *SLC6A14* expression (lines, derived as for Fig 2) for tissues of interest. (B) and (C) *Slc6a14* gene expression levels measured by RT-PCR of RNA from C57BL6/J mice (n = 4) from pancreas (B) and lung (C); expression is shown relative to *Gusb* at gestational (E) or post-natal (P) days indicated.

The chromosome X locus associated with meconium ileus also displayed association with CF lung function severity [41], with similar effects in males and females. However, the previously identified broad peak of association for lung function appeared shifted and was notably closer to the angiotensin II receptor type 2 (*AGTR2*) gene (panel (a) in Fig 3, blue/green dot palette for lung function association [41], and red/yellow dot palette for meconium ileus association). The *SLC6A14* eQTLs from the GTEx lung samples (panel (a) in Fig 3, brown dash-dotted line) did not appear to colocalize with the lung GWAS association evidence. Given that the GTEx protocol for lung tissue collection largely avoids the respiratory epithelia (which include the most relevant cell types in CF), we confirmed the lung eQTLs by RNA sequencing of nasal epithelia (a surrogate airway model in CF [42]) from 63 Canadians with CF included in the GWAS (panel (a) in Fig 3, blue dash line). The *SLC6A14* eQTLS from the nasal epithelia (blue dashed line) and pancreas (black solid line) tissues appeared to coincide, respectively, with lung disease and meconium ileus associated variants. eQTLs for *AGTR2* in lung from GTEx also colocalize with the lung association signals in this region (S7 Fig), however *AGTR2* is not expressed in the nasal epithelia.

To formally evaluate the colocalization patterns observed in Figs 2 and 3, we developed a frequentist regulatory integration analytic framework, referred to as the *Simple Sum* (*SS*, Material and Methods and S1 Appendix), to directly test whether eQTLs for a given gene and in a given tissue colocalize with meconium ileus-associated GWAS variants, and can also test whether one tissue (or expression for one gene) shows greater colocalization over another. Only summary statistics for the respective eQTL and association evidence are required, and the colocalization p-values are simple to calculate analytically. We developed the *SS* because detecting colocalization in the presence of high LD and/or allelic heterogeneity is challenging for several commonly used colocalization methods [43–48], the majority of which are Bayesian approaches, such as COLOC [43] and eCAVIAR [44], that estimate the posterior probability of co-localization under specific causal variation assumptions. COLOC and eCAVIAR can be used with summary statistics, but COLOC assumes a single causal variant in both eQTL and GWAS, and the presence of allelic heterogeneity reduces the co-localization posterior probability. eCAVIAR can accommodate allelic heterogeneity, but the posterior probability of co-localization will be averaged across several variants in high LD. In contrast, the goal of the *SS* approach is not to estimate which variant is causal, but rather to assess the evidence for a regulatory mechanism at the associated locus. The performance of the *SS* approach, including in the presence of high LD and allelic heterogeneity, was confirmed by extensive simulation studies (Material and Methods, S1 Appendix, and S5–S7 Tables), demonstrating correct type 1 error control across most settings (S8–S13 Figs, and S8 and S9 Tables) and good power performance (S11–S16 Tables), as well as agreement with permutation-based results in applications (Table 3 and S10 Table). Results of method comparison with the alternative approaches COLOC [43] and eCAVIAR[44] are also provided (Table 3, and S8, S11–S16 Tables).

**Table 3:** Results of Simple Sum colocalization and contrasting colocalization analyses for the three meconium ileus genome-wide significant loci, and colocalization posterior probabilities from COLOC and eCAVIAR. The eQTL evidence is the GTEx p-value based on the -log10(eQTL p) scale for a specified gene and tissue. The meconium ileus-SNP association evidence is the $\chi_{1}^{2}$-distributed Wald test statistic obtained in this study. Analytical (and permutation-based) Simple Sum (SS) colocalization p-values correspond to each individual tissue and evaluates if the eQTLs and meconium ileus-associated variants colocalize. Simple Sum contrasting (SSC) colocalization p-value evaluates if the eQTLs in the pancreas colocalize with meconium ileus-associated variants more than eQTLs in another tissue; NAs are listed for the pancreas since we do not contrast pancreas with itself. All p-values are one-sided because only positive association implies eQTL-association colocalization (i.e. eQTL peaks coincide with association peaks). COLOC [43] PP represents the posterior probability of eQTL-association colocalization calculated for each individual tissue. eCAVIAR [44] calculates SNP-level colocalization posterior probability (CLPP) for each variant in a locus for a given gene and in a given tissue. The regional colocalization probability (RCP) can be defined by summation of the SNP-level CLPP in a given LD block; RCP is recommended in [49] and the results are provided in the ‘eCAVIAR RCP’ column. eCAVIAR maximum regional CLPP provides similar qualitative results as eCAVIAR RCP thus are not shown here.

Gene	Tissue	SS Analytical colocalization p-value	SS Permutation colocalization p-value	SSC colocalization p-value (Pancreas vs. other tissue)	COLOC PP	eCAVIAR RCP
SLC6A14	Pancreas	1.12x10^-10^	0	NA	0.9966	0.4916
	Esophagus	1	1	9.97x10^-9^	0.0191	0.0035
	Transverse Colon	9.49x10^-9^	0	7.65x10^-9^	0.3755	0.0213
	Stomach	0.983	0.983	7.64x10^-8^	0.0265	0.0035
	Lung	1	1	7.68x10^-9^	0.014	0.0039
SLC26A9	Pancreas	4.48x10^-5^	2.00x10^-5^	NA	0.6926	0.0172
	Esophagus	0.0301	0.0303	5.47x10^-5^	0.0064	0.002
	Stomach	1	1	5.73x10^-6^	0.0275	0.0046
	Lung	0.641	0.642	4.05x10^-7^	0.0241	0.006
ATP12A	Pancreas	3.35x10^-8^	< 10^−5^	NA	0.285	0.0188
	Transverse Colon	1	1	1.67x10^-9^	0.06	0.0017
	Esophagus	1	1	8.85x10^-9^	0.0474	0.0026
	Lung	1	1	1.86x10^-8^	0.0372	0.0028

Consistent with the patterns seen in Fig 2, the eQTL evidence for *SLC6A14*, *SLC26A9* and *ATP12A* in the pancreas (as measured by −log10 (eQTL p) are significantly correlated with the GWAS evidence (as measured by meconium ileus-SNP Wald $\chi_{1}^{2}$ association statistic) at each of these three loci, with SS p-values = 1.12x10^-10^, 4.48x10^-5^ and 3.35x10^-8^, respectively for the three loci (Table 3, analytical results, confirmed by the permutation-based results also provided in Table 3). Posterior probabilities of colocalization using the alternative COLOC [43] and eCAVIAR [44] methods provide supportive although less conclusive findings (Table 3), particularly for the *ATP12A* locus. In addition, eCAVIAR, which calculates SNP-level colocalization statistics and can be challenged by substantial LD in regions of interest, did not provide a regional colocalization probability that exceeded 0.50 across any of the three regions or tissues; the eCAVIAR maximum occurred at rs3788766 at chromosome X for the pancreas.

At the chromosome X lung GWAS locus (Fig 3 and S7 Fig), application of the SS colocalization analysis indicates that eQTLs from GTEx for *AGTR2* in the lungs show evidence of colocalization with the lung-associated variants but not for *SLC6A14* (colocalization p-values = 1 and 2.82x10^-7^ for *SLC6A14* and *AGTR2*, respectively; S17 Table). Given sub-optimal content of epithelia in the GTEx lung tissue samples by design, we calculated *SLC6A14* eQTLs from the CF nasal epithelia which provided colocalization evidence with the lung disease associated variants(p = 2.4x10^-4^); *AGTR2* was not expressed in the nasal epithelia. Individually, the lung and meconium ileus GWAS association findings could be interpreted as simply indicating distinct genes for the two CF phenotypes. However, the integration of the tissue-specific eQTL patterns for *SLC6A14* (Fig 3) support a role for *SLC6A14* in both CF lung and pancreas. Further, as regulatory elements can exert effects over long distances, the differential positioning of the GWAS peaks with the co-alignment of eQTL peaks could accordingly reflect alternate modes of *cis*-regulation of *SLC6A14*.

Significant colocalization can occur in more than one tissue (Table 3, S10 Table and S14 Fig) or for expression of more than one gene. Therefore, it is also of interest to directly contrast the colocalization evidence between tissues or genes (Material and Methods, Table 3, S10 and S17 Tables), suggesting for example, the most probable tissue(s) of origin or target gene for an association locus identified via GWAS. Contrast colocalization analysis for the meconium ileus GWAS loci showed that eQTLs for *SLC6A14*, *SLC26A9* and *ATP12A* in the pancreas colocalize with meconium ileus-associated variants significantly more than eQTLs for these genes in the other meconium ileus-relevant tissues tested including the intestine (Table 3, SSC colocalization p-value results). The maximum posterior probability of colocalization from COLOC and eCAVIAR also occurred with the pancreas eQTLs versus the other tissues evaluated (Table 3). These findings are consistent with the pancreas-specific *PRSS1* locus in that variation in *SLC6A14*, *SLC26A9* and *ATP12A* gene expression in the pancreas appears to mediate the association between meconium ileus and the GWAS variants.

### Is exocrine pancreatic damage a causal contributor to meconium ileus?

We previously used the *SLC26A9* variant rs7512462 (S3 Table) as an instrumental variable in a Mendelian Randomization (MR) study to assess whether damage to the exocrine pancreas is a causal contributor to CF-related diabetes [15]. To determine whether variation in exocrine pancreatic injury *in utero* (measured by immunoreactive trypsinogen (IRT) at birth) is a causal contributor to meconium ileus, we now likewise applied a two-sample MR procedure [50, 51] using the same instrumental variable (Material and Methods). The two-sample method first assessed the effect of rs7512462 on meconium ileus in the Canadian Gene Modifier Study (CGMS) Phase I sample (n = 1,661, OR = 1.39, 95% CI = [1.14, 1.70]). Then a sub-sample of n = 126 was used, for whom IRT measurements were available, to estimate the effect of rs7512462 on log(IRT) levels estimated at birth (mean change in log(IRT) per additional rs7512462 risk allele (T) = -0.60, 95% CI = [-1.06, -0.14]). These lead to the estimate of the causal effect of pancreatic damage on meconium ileus risk (OR = e^[log(1.39)/-0.60]^ = 0.58, 95% CI = [0.34, 0.99]). This significant causal estimate is similar to that obtained from a logistic regression of estimated log(IRT) on meconium ileus status (OR = 0.54, 95% CI = [0.40, 0.70]), suggesting this direct association is largely free of confounding. Together these data contribute to evidence that variation in gene expression in the exocrine pancreas may be a causal contributor to meconium ileus, although the MR assumption of no horizontal pleiotropy [16] is difficult to confirm for single-instrument analyses. In support of this assumption, we note that expression of *SLC26A9* is reported in the pancreas (median TPM 1.60), with essentially none in the relevant terminal ileum tissue (*i*.*e*. small intestine; median TPM 0.02) in human (GTEx, [23]). Similarly, Slc26a9 was noted in the pancreas but was undetectable in the distal segments of the small intestine by RT-PCR analysis of murine tissues at adult stages (see below and [52]).

## Discussion

Together with the identification of *PRSS1* as a meconium ileus modifier, the integration of GTEx eQTL evidence and meconium ileus susceptibility associations through colocalization analysis support that variation at the identified modifier loci may reflect *SLC6A14*, *SLC26A9* and *ATP12A* gene expression, and in the CF pancreas rather than in the intestine or any other meconium ileus-relevant tissues tested. The underlying mechanism of this complex intestinal obstruction phenotype that requires loss of CFTR in the intestine [53] must then include the critical roles played by the transporters in early development and/or the early tissue damage that arises with loss of CFTR in the pancreas.

*SLC6A14* encodes a neutral and cationic amino acid transporter [54] that based on GTEx information is most notably expressed in human lung. Various cancers of epithelial origin, including breast [55], colon [56] and pancreas [57] also exhibit robust expression that is relatively elevated from their source tissues. The *SLC6A14* rs3788766 was the most significant SNP in this (Table 2, panel (a) in Fig 2) and our previous meconium ileus GWAS [12] (S3 Table), and its risk allele T corresponds to increased transcript level based on the GTEx eQTL data of adult pancreas tissue (panel (a) in Fig 2). Although human data from early stages of development would be preferable, we did examine pancreatic and lung tissue from the mouse spanning late embryonic to adult stages of development (panel (b) and (c) in Fig 3) to find maintained, albeit relatively low, *Slc6a14* transcript levels in the pancreas, with decreasing levels in lung over time. The rs3788766 SNP is in perfect LD (r^2^ = 0.8 and D’ = 1) with rs12710568, which is just 714bp 5’ of the *SLC6A14* TSS. ChIP-seq evidence [58] demonstrates the binding of primed AR (androgen receptor), which has been shown in seven independent studies (GSE58428, GSE84432, GSE56288, GSE28950, GSE56086, GSE65478, GSE32892) and this SNP falls within the androgen receptor binding motif (5'*-*GGA/TACA**[N]**NNTGTTCT-3'—SNP position marked in brackets [59]) and could potentially contribute to the sex-specific risk differences observed (S4 Table, S6 Fig).

In contrast to the chromosome 13 and X loci, there are additional genes and tissues that show evidence of colocalization with the GWAS variants (S14 Fig) within 1Mbp of the chromosome 1 locus. As a consequence it is more challenging to pinpoint the responsible gene and tissue from the colocalization evidence alone. However, published experimental evidence for *SLC26A9* complements the colocalization results. *SLC26A9* has been characterized as an anion channel in epithelial cells [60, 61]. *Slc26a9*^*-/-*^ */Cftr*^*-/-*^ double knock out models have a lower rate of survival post-birth than either *Cftr*^*-/-*^ or *Slc26a9*^*-/-*^ mice [52]. *SLC26A9* is expressed in several tissues including the lung, stomach, upper intestinal tract, kidney, and pancreas [25, 52, 62, 63]. Several studies indicate that CFTR and SLC26A9 interact in lung tissue, with biochemical and electrophysiological studies indicating that interaction enhances functional expression of CFTR [9–11]. Interestingly, this genome-wide study, as well as previous studies of early-onset pancreatic phenotypes that identified *SLC26A9* as a CF modifier [12–14], highlight that *SLC26A9* and *CFTR* may not require interaction in the pancreas in the same way apparent for the lung phenotype. The meconium ileus association is seen in patient cohorts composed of many different *CFTR* genotypes, and the effect size is similar in individuals who are Phe508del/Phe508del (OR = 1.41, se = 0.058) versus other *CFTR* genotypes (OR = 1.32, se = 0.083). Indeed, notable differences in murine mRNA levels between late gestation and adult stages for *Slc26a9* in the pancreas and the lung, in opposing directions, suggests the function of SLC26A9 may be different across time and tissue (panel (c) in Fig 4 of [2]). Detailed analysis of murine Slc26a9 expression in the intestine by others have also indicated changes in expression with young versus older ages [52]. Taken together with previous reports of *SLC26A9-*associated pancreatic phenotypes [13–15] we purport that SLC26A9 may provide alternative chloride transport at early developmental stages, and that its contribution to meconium ileus, is at least partly, from the pancreas.

*ATP12A* encodes the α-subunit of the non-gastric H^+^/K^+^ transporter. In a recent study, its expression in human and pig versus the negligible expression in the murine airway was argued to explain differences in airway surface liquid pH, viscosity and host defense in human and pig versus murine CF airways [34]. Although we were not able to detect Atp12a mRNA in total mouse pancreas using RT-PCR, ATP12A expression has been seen in human pancreatic ductal lines [64] and in isolated rat pancreatic ducts with localization toward the lumen [65]. The essential contributions of H^+^/HCO_3_^-^ movements and pH maintenance for fluid secretion in the pancreatic duct has been studied extensively, and any disturbance with decrease in pH would increase concern for effective digestive enzyme transport and risk of auto-activation. With the eQTL findings pointing to the pancreas, the common allele variants in *ATP12A* that associated with meconium ileus risk are also associated with increased ATP12A expression, where even modest increases in proton secretion, may be critical in the absence of CFTR [66].

Meconium ileus has complex etiology. The pig and murine models have highlighted the engagement of the gastrointestinal tract, where confined restoration of CFTR is sufficient to ameliorate both meconium ileus [53] and the intestinal obstruction phenotypes to substantial degrees [67, 68]. Although the genes identified in this GWAS are candidates for contributions to CF given their roles in epithelial transport and function, a working model in which their contribution to intestinal obstruction at birth is through common variation of their expression in the CF pancreas was not immediately obvious, beyond *PRSS1*. Understanding the organ of origin for any modifier gene is highly relevant as we embark on personalized medicine, first, toward designing the most optimal functional studies to test therapies and second, toward development of the complex models and signatures of prediction for both prognosis and therapeutic responses. Access to tissue-specific expression data for colocalization analysis here, beyond the nasal epithelia data, was limited to that available in the GTEx consortium project, and therefore our conclusions must be viewed in that context. Consideration of expression of modifier genes in early pancreatic stages rather than just adult tissue would be helpful for confirmation.

There is substantial variability in response to the recently developed CFTR-modulatory therapies between [69, 70] and within [71] individuals, and modifier studies highlight that individual level genetic background beyond *CFTR* genotype contributes to these differences. For example, we have shown that variation in lung response to modulator therapy can be explained in part by the *SLC26A9* modifier gene, possibly via interaction with CFTR [2]. Consideration of temporal and spatial expression of CFTR and modifier genes is needed when assessing CFTR function and therapeutic response for the purpose of choosing therapeutic strategies. This is specifically relevant where current testing paradigms use primary CF-tissue models such as nasal and lung epithelia, or rectal and lung organoid models [72–74]. Maximum benefit will be achieved with consideration of genetic background, as these models are being considered to decipher how to implement therapeutic decision-making and achieve personalized medicine [73].

Predictive genetic signatures that include organ specific aspects hold great promise to direct and complement therapeutic decision making in CF and other inherited diseases. They have additional potential in assisting clinical monitoring regimes, and toward stratification of patients for clinical trials to optimize outcome.

## Material and methods

### Ethics statement

Studies were approved by institutional review boards at participating sites and include Johns Hopkins School of Medicine eIRB2 (Committee: IRB-3); Research Ethics Board of the Hospital for Sick Children; Biomedical Institutional Review Board, Office of Human Research, University of North Carolina at Chapel Hill; University Hospitals Case Medical Center, Institutional Review Board for Human Investigation; and the French ethical committee (CPP n°2004/15) and the information collection was approved by CNIL (n°04.404). Written informed consent was obtained from adults, and for patients<18 years old consent was obtained from parents or guardians for participation in the study. The research ethics approval number for this cystic fibrosis gene modifier study was obtained from the Hospital for Sick Children Research Ethics Board, number 1000016662.

### Study sample

The International CF GMC consists of individuals from four consortium sites, Johns Hopkins University (JHU), the University of North Carolina/Case Western Reserve study (UNC/Case), the Canadian Gene Modifier Study (CGMS) and the French Gene Modifier Study (FGMS). The North American (NA) data were genotyped in two phases, Phase I-NA with 5,132 individuals and Phase II-NA with 2,830 individuals (before quality control; see below). Data from the FGMS were genotyped separately and include 1,300 individuals with CF. The Phase I-NA data were the basis of the first published meconium ileus genome-wide association study (GWAS) from the International CF GMC [12].

### Genotyping and quality control

Genotyping used four different Illumina platforms: the 610Quad, CNV370, 660W and Omni5, where 660W genotyping was performed in three batches (660W-NA, 660W-JHU and 660W-FR). Genotype calling was performed using GenomeStudio V2011.1. Quality control (QC) of genotypes and phenotypes was performed separately for each of the six genotyping platforms and batches: 610Quad, 660W-NA, 660W-JHU, Omni5, CNV370 and 660W-FR. Sample details after data quality control (QC) are listed in S1 Table, and QC procedures for SNP exclusion are presented in S2 Table.

SNP position and annotation information were based on Genome Reference Consortium 37 (GRCh37). The number of probes for each platform before QC was 570,572 for the 610Quad, 655,214 for 660W-NA and 660W-JHU, 4,301,332 for the Omni5, 309,012 for CNV370 and 554,649 for 660W-FR (S2 Table). SNPs were removed if they were not annotated to chromosomes 1–22 or X, with no rs number, or had a call rate <90%. Duplicated variants (occurred on the Omni5 chip after mapping kgp SNPs into dbSNPs) were also removed. Additionally, SNPs on chromosome X with heterozygosity rate more than 10% in the male sample were removed [75]. To reduce potential false negatives, the SNP QC criteria used here were relatively lenient, but any apparent association signals were subject to additional QC scrutiny to minimize potential false positives.

Detailed QC procedure for sample exclusion is as follows: individuals were removed if the initial call rates were <95%, or had extreme heterozygosity rates where the threshold was defined as in [12]: that is, 10 S.D. away from the mean autosomal heterozygous rate calculated from a set of pruned common SNPs (r^2^<0.2, MAF>0.05). Plink (v1.07, Web Resources in Material and Methods and [76]) inferred sex for each individual was compared to the reported gender and individuals with mismatched sex were then removed. After the aforementioned QC was performed separately for each platform and genotyping batch, all six subsets were then merged together and KING (Web Resources in Material and Methods and [77]) was used to identify any cryptic familial relationships among all individuals. Duplicated individuals/identical twins were identified and for each pair the ones with more genotype data were kept. Individuals without phenotypic information were excluded. All analyses were restricted to individuals with two severe CFTR mutations associated with pancreatic insufficiency (PI) since individuals with more mild mutations are in general at lower risk of meconium ileus [5]. Lastly, KING was used for population stratification using principal component analysis (PCA), and individuals identified as non-Europeans were excluded. Specifically, genotyped SNPs common across all platforms with MAF>0.05 and in low linkage disequilibrium (LD) with each other (r^2^<0.2) were included for PCA on the combined sample. Following the PCA procedure used in [12], non-European descendants were first excluded, defined as 6 S.D. away from the center of the HapMap3 [29] European (CEU/TSI) cluster. The Tracy-Widom statistic, computed using Eigensoft [78–81], was then used to determine the statistical significance of each principal component; six principal components were determined to be significant (p<0.05) and were included in subsequent association analyses as covariates. S1 Fig displays the pairwise comparison of the first three principal components.

### Imputation

A subset (n = 101) of the Canadian CF Gene Modifier cohort that mirrored the Canadian severe CF population also underwent whole genome sequencing by Complete Genomics (CG) at an average of 30X coverage. These participants were of European ancestry and had two *CFTR* mutations associated with pancreatic insufficiency [5, 12, 19], with 64.2% being homozygous for the most common *CFTR* mutation, Phe508del. The genome sequences were integrated with the 1000 Genomes (IKG) Phase 3 data [82] to generate a hybrid reference panel [83, 84] that was then used for genome-wide imputation of the full GMC sample using Beagle (Web Resources in Material and Methods) [85]. Details on the methodology can be found in [28]. The hybrid reference with in-sample whole genome sequence ensured the presence of CFTR disease-causing haplotypes that were not well represented in publicly available reference panels such as the 1000 Genomes Phase 3 reference [82] or the Haplotype reference consortium panel [86]. The importance for constructing this hybrid reference that includes individuals representative of the study population (CF in our case) is best illustrated by S2 Fig, which demonstrates the gain in fine-mapping achieved by augmenting the 1KG reference with WGS from 101 patients with CF, suggesting better coverage of study population-specific variants [28].

After obtaining this CF-specific hybrid reference panel, Beagle V4.1 [87] was used for phasing and imputing missing genotype data for our samples. We applied the same method as in [28] for whole genome pre-imputation phasing. Samples were phased and imputed separately for the six genotyping platforms. Approximately 9% of our samples displayed evidence of non-European ancestry by principal component analysis, thus all individuals in the reference panel were used for phasing and imputation.

Beagle used a sliding window of 250,000 markers from our genotype data with 25,000 markers overlapping with the next window for the phasing and then imputation steps. The phasing step included all individuals from our samples and from the reference panel to conduct 10 burn-in phasing iterations, followed by additional 5 phasing iterations to ensure accuracy, and the imputation step followed to fill in the markers not genotyped. Imputed SNPs with Beagle quality score AR2<0.3 were set to missing and SNPs with more than one alternative allele or with a minor allele frequency <1% were excluded from the association analysis.

### Association analysis

Genome-wide association analysis was carried out using Generalized Estimating Equations (GEE) with an exchangeable covariance structure to account for sibling relationships in the cohorts, as implemented previously [12]. Covariates included the six significant principal components, as well as three indicators for the four consortium sites (CGMS as baseline, JHU, UNC/Case and FGMS) and three indicators for the four genotyping platforms (namely, 610Quad as baseline, Omni5, CNV370 and 660W as defined in S1 and S2 Tables) to account for potential batch effects. Genotypes were coded additively for all the autosomal SNPs. For X chromosome SNPs females were coded additively, and males were coded as 0 and 2. Genome-wide significance was defined as p≤5x10^-8^ [30] and the accuracy of p-value calculation was up to 2.2×10^−16^ by the geeglm function [100] in R.

### Gene-based analysis of *CFTR*

In total, 241 (genotyped and imputed) SNPs with MAF ≥1% and within 10kb of the boundaries of *CFTR* were included in a gene-based permutation test as in [12] using the 5,869 unrelated individuals. For the observed data, the association analysis was performed using logistic regression adjusting for site and platform effects, for each of the 241 SNPs. The SNP-level Wald $\chi_{1}^{2}$ test statistics were aggregated, and the sum represented the gene-level association evidence. The observed meconium ileus phenotype values were then permuted within each of the site and platform groups, independently 10,000 times, and the corresponding sum of the Wald association statistics were obtained. The empirical p-value was the proportion of the permutation samples whose sum was larger than that calculated from the observed sample.

### Replication of the apical plasma membrane hypothesis

Previous studies demonstrated that multiple constituents of the apical plasma membrane that reside alongside CFTR also contribute to meconium ileus susceptibility [12]. To confirm this gene-set association, all 157 apical plasma membrane genes were extracted from the Refseq table in August, 2016 and the grand union of the boundaries in hg19 from multiple transcripts were used to extract corresponding SNPs. To be consistent with the original analysis in [12], the analysis here was limited to SNPs genotyped on the Illumina 610 Quad in the updated gene region with +/-10kb of the gene boundaries; corresponding SNPs for samples genotyped using other platforms were imputed. In total, 3,951 SNPs were included in the apical gene set analysis. A permutation test (using 10,000 replicates), similar to that for gene-based analysis above, was then carried out to obtain the empirical p-value for the gene-set.

### Chromosome X analysis

To investigate sex-specific effects of variants from the *SLC6A14* X chromosome locus, besides the association test above, GEE analyses were also conducted separately for males and females using either additive or genotypic coding. A contrast test comparing males with the risk allele (e.g. D) to females homozygous for the risk allele (DD) was also implemented to test for sex-SNP interaction effects. This test assumed a common baseline risk of meconium ileus between males and females without the risk alleles (e.g. d and dd), and the statistical model was constructed as

$$g(\mu )=\beta_{0}+\beta_{1}*I_{Female,1}+\beta_{2}*I_{Female,2}+\beta_{3}*I_{Male,1}+covariates,$$

where *I*_*Female*,*1*,_ *I*_*Female*,*2*_ and *I*_*Male*,*1*_ are indicator variables representing, respectively, the female dD and DD genotype groups and male D genotype group, and *g(μ)* is the logit link function. Under the X-inactivation assumption supported by evidence from the Roadmap Epigenomics Mapping Consortium [25], the DD genotype is expected to express one allele. Thus, testing whether *β*_*2*_ *= β*_*3*_ provides evidence for sex-specific differences in effect size or an interaction effect. This contrast test was performed using the doBy package (version 4.5–15) in R (Web Resources in Material and Methods).

### Estimation of phenotypic variance explained

Pseudo R^2^ was used as a measure of the phenotypic variance explained by the associated SNPs. The estimate was calculated based on multivariate logistic regression (covariates include the top significant SNP from each of the three genome-wide significant loci in addition to the six PCs and six site and platform indicators), applied to 5,869 unrelated individuals (a subset of the original sample of 6,770 that included siblings), using the rms package (version 4.5–0) in R.

### Mendelian randomization

Immunoreactive trypsinogen (IRT) is a biomarker of exocrine pancreatic damage (PD) [22]. In the CGMS subset, n = 126 individuals had longitudinal measures of IRT. A linear mixed effects (LME) regression model, accounting for the limits of detection of the IRT assay (<3ng/ml) by the statistical model [88], was used to estimate patient-specific IRT measurements at birth. IRT and age (days) were natural log transformed. The patient-specific birth IRT estimates from this model were then used as measures of prenatal pancreatic damage as in earlier biomarker studies [15].

To determine if variation in the exocrine pancreas contributes to the cause of meconium ileus, we estimated the causal effect using an application of instrumental variable analysis known as Mendelian Randomization (MR) [51]. We compared the estimate of causal effect to the (possibly confounded) direct effect estimate obtained by regressing meconium ileus status on estimated values of log(IRT) at birth. The causal effect on meconium ileus was estimated using the two-sample MR method with *SLC26A9* rs7512462 genotype (coded additively) as the instrumental variable [50, 51], applied to the CGMS Phase I genotyped sample (i.e. sample one, n = 1,661) and the sub-sample for whom IRT measurements were available (i.e. sample two, n = 126). Dividing log(OR), the association estimate obtained from the logistic regression of meconium ileus status on rs7512462 using sample one, by the average increase in log(IRT) at birth per additional rs7512462 risk allele, obtained from linear regression of log(IRT) on rs7512462 using sample two, gives the log(OR) that represents the association of genetically determined log(IRT) levels at birth with meconium ileus risk [51]. We obtained a cluster robust standard error for the estimated OR in sample one, and used the delta method to estimate the standard error for the two-sample causal OR estimate [89].

The application of MR required that the *SLC26A9* instrument be robustly associated with the exposure (exocrine PD). Although we obtained a modest F statistic of 6.7 from the regression of log(IRT) at birth on rs7512462 genotype, the log(IRT)-rs7512462 association has now been demonstrated in two independent samples from Canada [15] and the United States [14]. In addition, our use of the delta method to estimate the confidence interval surrounding the causal estimate is conservative [89]. MR assumes that the relationship between the instrument (*SLC26A9*) and meconium ileus was mediated by variation in prenatal exocrine PD, and not due to any pathway independent of PD. This critical assumption of ‘no horizontal pleiotropy’ [16], although difficult to prove in practice [90], is supported here by a lack of *SLC26A9* gene expression in intestinal tissue consistent with the ‘sites’ of meconium ileus in the ileum and proximal colon [52].

### Enhancer and promoter regions

The enhancer and promoter region data are obtained from the Broad Institute website [24, 25]. The enhancer regions included were defined as those with DNaseI signal with -log_10_P>2 and falling in enhancer states 6,7,12 in the 5-mark 15-state chromHMM observed data across 127 epigenomes from the Roadmap [25] and ENCODE [24] tissues. The promoter regions were defined as those falling within a DNaseI signal with–log_10_P>2 and 15-state chromHMM states 1,2,10.

### Genotype tissue expression consortium (GTEx) data

We investigated all genes in the relevant tissues that satisfied the GTEx v6 threshold for expression, which is a gene expression threshold of >0.1 RPKM in at least 10 individuals and > = 6 reads in at least 10 individuals. This criteria was slightly more liberal than in v7 which calculated eQTLs when there was >0.1 TPM in at least 20% of samples and > = 6 reads in at least 20% of samples. All eQTLs were obtained from the GTEx v7 portal with the exception of *SLC6A14* in the pancreas and *ATP12A* in the transverse colon, which satisfied v6 but were just shy of satisfying the v7 criteria. We calculated these eQTLs following the GTEx protocol. Briefly, normalization of the publicly available GTEx expression matrix (version 7, 15-Jan-2016 data freeze, phe000020.v1) was subsetted for the samples for the specific tissue of interest and expression values were normalized between samples [91], and each gene was then inverse quantile normalized across samples using normalize_expression.py script on GTEx’s Broad Institute’s GitHub page (Web Resources in Material and Methods). Probabilistic Estimation of Expression Residuals (PEER) [92] were calculated using run_PEER.R script from GTEx, while principal components were calculated using the PC-AiR package in R [93], with the kinship matrix input calculated using KING version 2.0 [77]. Sex and sequencing platform covariates were obtained from phs000424.v7. Genotypes were obtained from variants called and QC’d from WGS (phg000830.v1). The FastQTL version 2.0 program [94] was used for mapping *cis*-eQTLs for each variant-gene pair. Nominal p-values with a mapping window of 1 Mbp were used. The eQTL p-values obtained closely matched those provided on the GTEx v6 portal, although the v7 results provided more significant p-values for *SLC6A14* in the pancreas.

### RNA-sequencing

We conducted eQTL analysis with RNA sequencing of human nasal epithelial (HNE) cells from 63 CF Canadians enrolled in the GMC. Samples that were included had HNE cells collected that were drug-naïve, and their RNA had an RNA integrity number (RIN) > = 7. Sample processing and RNA sequencing was conducted in two batches following two protocols, n = 32 as part of the CF-Canada SickKids Program for Individualized Therapy (CFIT; [95]) and n = 31 from a UNC-led initiative [96]. In brief, HNE cells for the CFIT were collected by cytology brushes, and RNA sequencing was conducted by the Illumina HiSeq 2500 with the High Throughput Run Mode, producing 35 million paired-end reads with a length of 124 base pairs per sample. The HNE cells from the UNC-led initiative were collected by a Rhino-probe curette, and the Illumina HiSeq 2000 platform was used for RNA sequencing, producing 25 million paired-end reads with 49 base pair length per sample.

The sequencing reads were aligned to the hg19 genome reference using STAR v2.4.2a [97], aided by the GENCODE 19 gene annotation. Gene expression read counts and RPKM were calculated by RNA-SeQC [98] with the strictMode option, based on a collapsed GENCODE 19 gene model as describe by the GTEx consortium [23]. The eQTL analysis followed the GTEx v6p pipeline [23]. Genes with RPKM >0.1 and read counts > = 6 in at least 10 individuals were included. RPKM of selected genes at the chromosome X locus were first quantile normalized to the average empirical distribution across samples, then inverse quantile normalized to the standard normal across samples for each gene. The cis-eQTL mapping was conducted by FastQTL [94] with its default settings. The Beagle imputed SNPs with MAF>5% and within a one mega-base window up and downstream of the transcription start sites were included. Covariates included the first three genotype principal components calculated by KING, 15 hidden variables estimated by PEER [99], normalized RPKM of CD45 (an adjustment for immune cell composition) and sex.

### Colocalization *Simple Sum* testing

At each of the genome-wide significant loci, to statistically determine whether the GWAS associations were mediated through gene expression, and in which tissue, formal colocalization analysis was required to integrate the association evidence with available tissue-specific gene expression data. We implemented a frequentist colocalization framework, the *Simple Sum* (SS). We outline the method and evaluation strategies below, and we provide details in the Supporting Information including analytic derivations of the method, simulation design and parameter settings, and results of method evaluation and comparisons. The *Simple Sum* application and simulation results were compared to two other frequently implemented Bayesian colocalization methods designed for summary level data, COLOC [43] and eCAVIAR [44].

To carry out the *Simple Sum* approach, the meconium ileus-SNP association statistics are the Wald $\chi_{1}^{2}$ statistics obtained from this GWAS study. To facilitate the permutation-based method that was used to confirm the analytic results, we used the association results obtained from 5,869 unrelated CF participants. The tissue-specific gene expression-SNP association summary statistics (the eQTL p-values) were obtained from the GTEx version 7 (15-Jan-2016 data freeze) [23], except in the case of *SLC6A14* in the pancreas and *ATP12A* in the transverse colon; we calculated these using v7 data as explained above in the GTEx data section. eQTLs from the nasal epithelia of the 63 CF Canadians were used for colocalization analysis at the chromosome X locus to complement the lung eQTLs obtained from GTEx.

### The *Simple Sum* analytical framework

#### GWAS association-eQTL SS colocalization test for a single-tissue (or gene)

At each genome-wide significant locus, we extract all *m* genotyped or imputed SNPs that are within 0.1 Mbp on either side of the lead (most significant) SNP at the locus. Using the pancreas as an example, an intuitive approach is to assign each SNP to one of the two groups according to whether it is an eQTL or not based on the GTEx data. The definition of an eQTL can be subjective, e.g. GTEx p<0.05 or <0.005, but for the moment let us first consider using a p-value threshold for eQTL. In that case, let *t_jk_* be the gene-expression indicator variable for SNP *j* and tissue *k*, then a *Simple Sum* (*SS*) colocalization test statistic at a given locus can be defined as

$$SS=\frac{1}{\sum_{j}I(t_{jk}=1)}\sum_{j}S_{j}I(t_{jk}=1)-\frac{1}{\sum_{j}I(t_{jk}=0)}\sum_{j}S_{j}I(t_{jk}=0),$$ (1)

where *S*_*j, j = 1,…,m*_ is the phenotype-SNP association statistic (e.g. the Wald $\chi_{1}^{2}$ statistic, $S_{j}=Z_{j}^{2}$) for the *j*th SNP. This *SS* statistic represents the difference in average association test statistics for eQTL and non-eQTL variants, and a significant *positive* value intuitively suggests colocalization of association and eQTL evidence, implying the test must be *one-sided* which we elaborate on later.

The p-value for the *SS* test can be evaluated by a permutation procedure similar to that used in the gene-based and gene-set analyses above. That is, we permute the phenotype to preserve the LD pattern between SNPs within each cohort and platform, independently, say 10^5^ times. For each permutation sample, the corresponding phenotype-SNP association Wald statistics and the *SS* colocalization quantity shown in Eq (1) are re-calculated. The empirical colocalization (one-sided) p-value is the proportion of the permutation samples whose *SS* test statistics are *larger* than that in the observed sample. However, this permutation-based approach can be computationally challenging.

Alternatively, we derived the exact distribution of the *SS* statistic by expressing *SS* as a quadratic form **Z**′A**Z,** where ***Z*** = (*Z*_**1**_,*Z*_**2**_, …,*Z*_***m***_)’ is a vector containing the phenotype-SNP association statistics for the *m* SNPs in the region, and A = diag(a_1_,a_2_,…a_m_) with $a_{j}=\frac{t_{jk}-\bar{t_{k}}}{\sum_{j}t_{jk}^{2}-n{\bar{t}}_{k}^{2}}$. **Z**′A**Z** is distributed as a mixture of chi-squared distributions under the simple null hypothesis of no association and no-colocalization (S1 Appendix), thus the *SS* p-value can be obtained analytically without the need for permutation. Importantly, this analytic form allows for the use of a continuous measure of gene expression evidence (e.g. *t*_*jk*_ = −log_10_(GTEx p)) in the *SS* statistic, making the subjective definition of eQTL (e.g. eQTL p<0.05 or 0.005) unnecessary. Moreover, the intuitive interpretation when using the p-values rather than a cut-off is that the colocalization test assesses the similarity in pattern of eQTL and GWAS association, as is visualized in Fig 2. The validity of the proposed analytic method was confirmed by both the application (Table 3 and S10 Table, analytic vs. permutation p-value) and simulation studies (S8–S13 Figs and S8 and S9 Tables for empirical type 1 error control).

We first note that although the proposed *SS* method is motivated by a statistic that uses the difference in the sum of association test statistics for eQTLs and non-eQTLs, it inherently adjusts for LD between SNPs through properly calibrating the variance of the *SS* test statistic based on the LD pattern in the region of interest; this is similar to a phenotype-permutation-based empirical approach that preserves the LD pattern between SNPs.

We also note that the test must be *one-sided*. Besides the intuitive argument above, consider the case where both association and eQTL are significant but at distinct, uncorrelated SNPs (i.e. the H3 scenario of COLOC [43]). In that case, a large *t_jk_* corresponds to a small *S*_*j*_, and it is easy to see that the *SS* test statistic would be negative, resulting in a large p-value based on the proposed one-sided test. Thus, although the complement to the alternative of interest includes association without colocalization, the one-sided test ensures that this scenario is ruled out. We conducted simulation studies to provide corroborating empirical evidence (Case 4 simulation design in S6 Table with corresponding type 1 error results in S8 Table).

#### GWAS association-eQTL colocalization contrasting SSC test, contrasting two tissues (or genes)

To determine if there is better colocalization with tissue *k* (or gene) than with tissue *h* (or gene), intuitively, we can define a *SS* contrasting colocalization statistic that assesses the difference in average phenotype-SNP association test statistics for SNPs that are eQTLs for tissue *k* versus for tissue *h*,

$$SSC=\frac{1}{\sum_{j}I(t_{jk}=1)}\sum_{j}S_{j}I(t_{jk}=1)-\frac{1}{\sum_{j}I(t_{jh}=1)}\sum_{j}S_{j}I(t_{jh}=1),$$

where *t_jk_* and *t_jh_* are the gene-expression indicator variables for SNP *j* in tissue *k* and *h*, respectively (e.g. eQTL p<0.05 or 0.005). Again, we were able to reformulate this *SSC* test statistic in a quadratic form that is also distributed as a mixture of chi-squared distributions under the null hypothesis (S1 Appendix). Thus, we can obtain p-values for *SSC* efficiently without the need for permutations; p-values can be calculated by using the R package “CompQuadForm”. Similar to the single-tissue colocalization case, we can also use the original continuous measure of gene expression evidence without arbitrarily defining an eQTL (e.g. eQTL p<0.05 or 0.005). S15–S20 Figs provide simulation results for all three loci analyzed and demonstrate that the proposed analytic method controls the type 1 error.

The proposed single-tissue and tissue contrasting colocalization *SS* framework is quite general, and it requires only summary statistics for the respective eQTL and phenotype-SNP association evidence. The *t*_*jk*_ can be −log10(eQTL p) as presented or a Wald eQTL test statistic as was used for the phenotype-SNP association component. The developed analytical approach to obtain p-values for *SS* makes the implementation easier when applied genome-wide or across several tissues and genes at a locus (S14 Fig). In addition, the proposed method implicitly allows for allelic heterogeneity (S7 and S11–S16 Tables). If there were two (or more) causal variants in a locus of interest and both contributed through regulating gene expression of the same gene (e.g. Alter 6 scenario in S7 Table), then the phenotype-SNP association and eQTL evidence would be statistically correlated, thus be identified by the *SS* method given sufficient sample size (e.g. Power in S16 Table for the Alter 6 scenario).

### Method evaluation and comparisons

We outline the evaluation strategies here and provide additional details of the simulation designs and results in the Supporting Information.

#### Evaluation through data application

We applied the proposed Simple Sum colocalization and contrasting colocalization methods to the three meconium ileus genome-wide significant loci, *SLC6A14*, *SLC26A9* and *ATP12A* (Table 3, using -log10(eQTL p) as a continuous measure of the gene expression evidence); a heatmap of the colocalization test is shown in S14 Fig. To validate the analytical p-values, we also provide permutation-based p-values in Table 3. To be comprehensive, S10 Table also provides the corresponding results when we dichotomize the gene expression evidence using various thresholds (e.g. eQTL p< 0.05, <0.005 or <0.0005). For the *SCL6A14* locus on Chromosome X, S17 Table provides the results when the gene expression evidence for lung was obtained from GTEx or HNE, and when the contrasting colocalization test was performed comparing expression of *SLC6A14* with other *genes* in the lung tissue models. This example also illustrates the flexibility of the proposed contrasting colocalization method: it can be used to contrast tissues (as in Table 3 and S10 Table) or genes (as in S17 Table).

For method comparisons with alternatives existing in the literature, we focused on COLOC [43] and eCAVIAR [44], two frequently implemented Bayesian approaches that can be used with summary statistics. There are several other colocalization methods (the majority being Bayesian) including ENLOC [49] and SHERLOCK [45]. In a recent study [49], it has been shown that eCAVIAR is extremely conservative with low false positive rate and low power, which we also observe here in our simulation studies (S8 and S11–S16 Tables) with the exceptions of S13 and S16 Tables. In these two scenarios (Alter 3 and Alter 6 in S7 Table), the allelic heterogeneity is such that colocalization exists at the second peak, which was simulated in a region of low LD; the parameter setting for which eCAVIAR is reported to perform well. COLOC is a special case of ENLOC, is easy to implement for simulation studies, and agrees with ENLOC results in the presence of strong signals. Results in Table 3 include the corresponding posterior probability (PP) of eQTL-association colocalization based on COLOC, and the regional posterior probability (RCP) of eCAVIAR as defined and recommended in [49].

#### Evaluation through simulation: Type 1 error of SS and false positive rate of COLOC and eCAVIAR

For type 1 error evaluation, we considered a comprehensive set of null cases where Case 1 represents the simple null of no association and no eQTL, while Cases 2–4 correspond to composite null scenarios where for example both association and eQTL may be significant but distinct. Detailed descriptions and illustrations of the scenarios and their corresponding parameter values considered are provided in S5 and S6 Tables.

For Case 1, S8, S10 and S12 Figs demonstrate type 1 error control of the *SS* test when data were simulated based on the different LD patterns at the *SLC6A14*, *SLC26A9* and *ATP12A* loci, respectively, and when the eQTL evidence was measured as −log10(eQTL p). To be comprehensive, S9, S11 and S13 Figs provide the corresponding type 1 error control results when eQTL p-values were dichotomized using a liberal threshold of p<0.05 when calculating the SS test statistic. Similarly, S15–S20 Figs provide evidence of type 1 error rate control of the *SSC* contrasting test, for the three loci and using either continuous or dichotomized measures of the eQTL evidence.

For the other cases, without loss of generality we focus on using the LD pattern at the *SLC6A14* locus. S8 and S9 Tables provide the empirical type 1 error rates of the *SS* method, as well as the false positive rates of COLOC and eCAVIAR using three different cut-off values (0.5, 0.75 and 0.9) for the posterior probability (S8 Table).

#### Evaluation through simulation: Power of SS and true positive rate of COLOC and eCAVIAR

For power evaluation, we considered a variety of alternatives including allelic heterogeneity in addition to the simple case where one single GWAS signal colocalizes with one single eQTL signal. For example, there might be two eQTLs but only one of the two colocalizes with a GWAS signal, and vice versa. Detailed descriptions and illustrations of the scenarios considered are given in S7 Table, and the corresponding results are provided in S11–S16 Tables including the power of *SS* and the true positive rate of COLOC and eCAVIAR.

### Web resources

CFTR2, https://cftr2.org; Plink, http://zzz.bwh.harvard.edu/plink/; KING, http://people.virginia.edu/~wc9c/KING/kingpopulation.html; Beagle, https://faculty.washington.edu/browning/beagle/b4_1.html; R, https://cran.r-project.org/; Roadmap Epigenomics Mapping Consortium (REMC), http://www.roadmapepigenomics.org/; The Encyclopedia of DNA Elements (ENCODE), https://www.encodeproject.org; Haploreg, http://archive.broadinstitute.org/mammals/haploreg/haploreg.php; Genotype-Tissue Expression Project (GTEx) Portal, https://www.gtexportal.org/home/; Regulatory Regions Map (Reg2Map), https://personal.broadinstitute.org/meuleman/reg2map/HoneyBadger2-intersect_release/; The International Hapmap Project, https://www.genome.gov/10001688/international-hapmap-project/; UCSC browser, https://genome.ucsc.edu/; Gene Expression Omnibus (GEO), https://www.ncbi.nlm.nih.gov/geo/; JASPAR, http://jaspar.genereg.net/. GTEx’s Broad Institute’s GitHub page, https://github.com/broadinstitute/gtex-pipeline

## Supporting information

S1 Appendix The Simple Sum (SS) analytical framework and method evaluation and comparisons. (DOCX)

S1 Fig Pairwise comparison of the first three principal components from a PCA analysis of all CF samples together with the reference samples from the International Hapmap consortium. Different solid dots show the clusters of the samples from the International Hapmap consortium [7]. The red circles correspond to our samples with outliers highlighted as black dots; outliers defined as 6 S.D. away from the center of the HapMap3 European (CEU/TSI) cluster. See the detailed description of the Hapmap samples at http://www.sanger.ac.uk/resources/downloads/human/hapmap3.html.
(TIF)

S2 Fig Comparison of *CFTR*-regional association with meconium ileus between two different imputation references. The Locus zoom plot [8] of *CFTR* association imputed using (A) the 1000 Genome Project Phase 3 [3], and (B) the hybrid reference (augmenting the 1000 genome reference with the whole genome sequencing from 101 patients with CF). Imputed variants with MAF>1% were analyzed.
(TIF)

S3 Fig Locus Zoom plot of meconium ileus association in a 200 kb region surrounding *ATP12A* in individuals homozygous for Phe508del. (TIF)

S4 Fig Conditional association analysis of the SNPs from the three genome-wide significant loci for meconium ileus. All subfigures are plotted in 200kb regions surrounding (A) *SLC6A14*, (B) *SLC26A9*, and (C) *ATP12A*. The GWAS signals are obliterated after conditioning on the top SNP in each region. The color of each dot represents the amount of LD of the SNP to the purple diamond point, which is the top SNP in each region after the conditional analysis.
(TIF)

S5 Fig Locus zoom plot of meconium ileus association in a 200 kb region surrounding *PRSS1* in the GWAS using the whole GMC sample of 6,770 individuals with CF. (TIF)

S6 Fig *SLC6A14* sex-stratified eQTLs in the pancreas show co-localization of eQTLs in males, but not females. GTEx expression data was analyzed separately by sex for eQTL association with *SLC6A14* in the pancreas using the same linear regression model described in Materials and Methods, GTEx Data without the sex covariate. Dots in the figure represent the association with MI, while the lines depict the association pattern of eQTLs for *SLC6A14* in the pancreas when analyzed in males (black solid line) and in females (dashed blue line) separately.
(TIF)

S7 Fig Meconium ileus and lung associations with GTEx eQTL profiles for *AGTR2* shows lung association co-localizes with lung-specific *cis*-eQTLs for *AGTR2*. Overlay of p-values (on the −log_10_ scale) from the meconium ileus GWAS (red/yellow palette of colored dots; this study), lung function GWAS (green/blue palette of colored dots; [9]) and GTEx (v7, [4]) eQTLs association for *AGTR2* expression (colored lines, derived the same as for Fig 2) for the different tissues of interest. *AGTR2* is not expressed in the CF nasal epithelia.
(TIF)

S8 Fig Type 1 Error evaluation of the Simple Sum colocalization analytical method based on the LD pattern at the *SLC6A14* locus and when the eQTL evidence is measured as -log_10_ transform of eQTL p-value. Simulation method is outlined in S1 Appendix, and the null case considered is Case 1 described in S6 Table where there is no signal for either GWAS or eQTL. In total, 10^4^ replications were simulated to obtain (A) QQ-plot of the SS colocalization p-value on the original scale, (B) QQ-plot of the SS colocalization p-value on the −log_10_ scale, and (C) the histogram of the SS colocalization p-value that is expected to follow a Unif(0,1) distribution under the null. The empirical Type 1 error is 0.0501 at the 0.05 nominal level, and 0.0053 at the 0.005 level.
(TIF)

S9 Fig Type 1 Error evaluation of the Simple Sum colocalization analytical method based on the LD pattern at the *SLC6A14* locus and when the eQTL evidence is dichotomized using the eQTL p<0.05 threshold. Simulation method is outlined in S1 Appendix, and the null case considered is Case 1 described in S6 Table where there is no signal for either GWAS or eQTL. In total, 10^4^ replications were simulated to obtain (A) QQ-plot of the SS colocalization p-value on the original scale, (B) QQ-plot of the SS colocalization p-value on the −log_10_ scale, and (C) the histogram of the SS colocalization p-value that is expected to follow a Unif(0,1) distribution under the null. The empirical Type 1 Error is 0.048 at the 0.05 nominal level, and 0.0049 at the 0.005 level.
(TIF)

S10 Fig Type 1 Error evaluation of the Simple Sum colocalization analytical method based on the LD pattern at the *SLC26A9* locus and when the eQTL evidence is measured as −log_10_ transform of eQTL p values. Simulation method is outlined in S1 Appendix, and the null case considered is Case 1 described in S6 Table where there is no signal for either GWAS or eQTL. In total, 10^4^ replications were simulated to obtain (A) QQ-plot of the SS colocalization p-value on the original scale, (B) QQ-plot of the SS colocalization p-value on the −log_10_ scale, and (C) the histogram of the SS colocalization p-value that is expected to follow a Unif(0,1) distribution under the null. The empirical Type 1 error is 0.052 at the 0.05 nominal level, and 0.0064 at the 0.005 level.
(TIF)

S11 Fig Type 1 Error evaluation of the Simple Sum colocalization analytical method based on the LD pattern at the *SLC26A9* locus and when the eQTL evidence is dichotomized using the eQTL p<0.05 threshold. Simulation method is outlined in S1 Appendix, and the null case considered is Case 1 described in S6 Table where there is no signal for either GWAS or eQTL. In total, 10^4^ replications were simulated to obtain (A) QQ-plot of the SS colocalization p-value on the original scale, (B) QQ-plot of the SS colocalization p-value on the −log_10_ scale, and (C) the histogram of the SS colocalization p-value that is expected to follow a Unif(0,1) distribution under the null. The empirical Type 1 error is 0.0509 at the 0.05 nominal level, and 0.0051 at the 0.005 level.
(TIF)

S12 Fig Type 1 Error evaluation of the Simple Sum colocalization analytic method based on the LD pattern at the *ATP12A* locus and when the eQTL evidence is measured as -log_10_ transform of eQTL p values. Simulation method is outlined in S1 Appendix, and the null case considered is Case 1 described in S6 Table where there is no signal for either GWAS or eQTL. In total, 10^4^ replications were simulated to obtain (A) QQ-plot of the SS colocalization p-value on the original scale, (B) QQ-plot of the SS colocalization p-value on the −log_10_ scale, and (C) the histogram of the SS colocalization p-value that is expected to follow a Unif(0,1) distribution under the null. The empirical Type 1 error is 0.0487 at the 0.05 nominal level, and 0.0034 at the 0.005 level.
(TIF)

S13 Fig Type 1 Error evaluation of the Simple Sum colocalization analytical method based on the LD pattern at the *ATP12A* locus and when the eQTL evidence is dichotomized using the eQTL p<0.05 threshold. Simulation method is outlined in S1 Appendix, and the null case considered is Case 1 described in S6 Table where there is no signal for either GWAS or eQTL. In total, 10^4^ replications were simulated to obtain (A) QQ-plot of the SS colocalization p-value on the original scale, (B) QQ-plot of the SS colocalization p-value on the −log_10_ scale, and (C) the histogram of the SS colocalization p-value that is expected to follow a Unif(0,1) distribution under the null. The empirical Type 1 error is 0.0513 at the 0.05 nominal level, and 0.0046 at the 0.005 level.
(TIF)

S14 Fig Heatmaps of the Simple Sum colocalization test for a 1Mbp region encompassing the peak meconium ileus-associated variants. The *SS* colocalization test evaluates if the eQTLs for a given gene and in a given tissue colocalize with meconium ileus-associated variants in the regions of (A) chromosome X, (B) chromosome 1, and (C) chromosome 13. In each panel, each row shows the *SS* colocalization evidence for the specified tissue across all genes within 1Mbp of the peak GWAS variant. *SS* colocalization evidence for each gene is calculated for SNPs within 0.1Mbp of the peak GWAS variants; the genes on the x-axis are ordered by their chromosomal positions. Each column shows the *SS* colocalization evidence for the specified gene across each tissue tested. The color intensity corresponds to the *SS* colocalization evidence as measured by −log_10_(*SS* p-value), with red representing −log_10_(p) = 6 and white representing −log_10_(p) = 0. Grey indicates either insufficient expression levels attained for the gene in the tissue under study, or that there were no significant eQTLs for the gene in that tissue. The eQTL analyses used for all gene/tissue pairs are those conducted by GTEx version 7 release, except the boxes indicated on the margins. eQTL analysis for the boxes on the margins were calculated in version 6 but were not calculated in GTEx version 7 due to a more stringent expression threshold criteria set in GTEx v7 versus v6 (see Material and Methods for specifics); these analyses were conducted using the publicly available expression matrix (phe000020.v1) and genotypes from WGS (phg000830.v1) following GTEx’s protocol for eQTL analysis.
(TIF)

S15 Fig Type 1 Error evaluation of the Simple Sum contrasting colocalization analytical method based on the LD pattern at the *SLC6A14* locus and when the eQTL evidence is measured as -log_10_ transform of eQTL p values. Simulation method is outlined in S1 Appendix, and the null case considered is Case 1 described in S6 Table where there is no signal for either GWAS or eQTL. In total, 10^4^ replications were simulated to obtain (A) QQ-plot of the simple sum contrasting (*SSC*) colocalization p-value on the original scale, (B) QQ-plot of the *SSC* colocalization p-value on the −log_10_ scale, and (C) the histogram of the *SSC* colocalization p-value that is expected to follow a Unif(0,1) distribution under the null hypothesis. The empirical Type 1 Error is 0.0536 at the 0.05 nominal level, and 0.0048 at the 0.005 level.
(TIF)

S16 Fig Type 1 Error evaluation of the Simple Sum contrasting colocalization analytical method based on the LD pattern at the *SLC6A14* locus and when the eQTL evidence is dichotomized using the eQTL p<0.05 threshold. Simulation method is outlined in S1 Appendix, and the null case considered is Case 1 described in S6 Table where there is no signal for either GWAS or eQTL. In total, 10^4^ replications were simulated to obtain (A) QQ-plot of the simple sum contrasting (*SSC*) colocalization p-value on the original scale, (B) QQ-plot of the *SSC* colocalization p-value on the −log_10_ scale, and (C) the histogram of the *SSC* colocalization p-value that is expected to follow a Unif(0,1) distribution under the null hypothesis. The empirical Type 1 error is 0.0454 at the 0.05 nominal level, and 0.0043 at the 0.005 level.
(TIF)

S17 Fig Type 1 Error evaluation of the Simple Sum contrasting colocalization analytical method based on the LD pattern at the *SLC26A9* locus and when the eQTL evidence is measured as −log_10_ transform of eQTL p values. Simulation method is outlined in S1 Appendix, and the null case considered is Case 1 described in S6 Table where there is no signal for either GWAS or eQTL. In total, 10^4^ replications were simulated to obtain (A) QQ-plot of the simple sum contrasting (*SSC*) colocalization p-value on the original scale, (B) QQ-plot of the *SSC* colocalization p-value on the −log_10_ scale, and (C) the histogram of the *SSC* colocalization p-value that is expected to follow a Unif(0,1) distribution under the null hypothesis. The empirical Type 1 error is 0.0457 at the 0.05 nominal level, and 0.0039 at the 0.005 level.
(TIF)

S18 Fig Type 1 Error evaluation of the Simple Sum contrasting colocalization analytical method based on the LD pattern at the *SLC26A9* locus and when the eQTL evidence is dichotomized using the eQTL p<0.05 threshold. Simulation method is outlined in S1 Appendix, and the null case considered is Case 1 described in S6 Table where there is no signal for either GWAS or eQTL. In total, 10^4^ replications were simulated to obtain (A) QQ-plot of the simple sum contrasting (*SSC*) colocalization p-value on the original scale, (B) QQ-plot of the *SSC* colocalization p-value on the −log_10_ scale, and (C) the histogram of the *SSC* colocalization p-value that is expected to follow a Unif(0,1) distribution under the null hypothesis. The empirical Type 1 error is 0.0512 at the 0.05 nominal level, and 0.0052 at the 0.005 level.
(TIF)

S19 Fig Type 1 Error evaluation of the Simple Sum Contrasting colocalization analytical method based on the LD pattern at the *ATP12A* locus and when the eQTL evidence is measured as −log_10_ transform of eQTL p values. Simulation method is outlined in S1 Appendix, and the null case considered is Case 1 described in S6 Table where there is no signal for either GWAS or eQTL. In total, 10^4^ replications were simulated to obtain (A) QQ-plot of the simple sum contrasting (*SSC*) colocalization p-value on the original scale, (B) QQ-plot of the *SSC* colocalization p-value on the −log_10_ scale, and (C) the histogram of the *SSC* colocalization p-value that is expected to follow a Unif(0,1) distribution under the null hypothesis. The empirical Type 1 error is 0.052 at the 0.05 nominal level, and 0.0048 at the 0.005 level.
(TIF)

S20 Fig Type 1 Error evaluation of the Simple Sum Contrasting colocalization analytical method based on the LD pattern at the *ATP12A* locus and when the eQTL evidence is dichotomized using the eQTL p<0.05 threshold. Simulation method is outlined in S1 Appendix, and the null case considered is Case 1 described in S6 Table where there is no signal for either GWAS or eQTL. In total, 10^4^ replications were simulated to obtain (A) QQ-plot of the simple sum contrasting (*SSC*) colocalization p-value on the original scale, (B) QQ-plot of the *SSC* colocalization p-value on the −log_10_ scale, and (C) the histogram of the *SSC* colocalization p-value that is expected to follow a Unif(0,1) distribution under the null hypothesis. The empirical Type 1 error is 0.0503 at the 0.05 nominal level, and 0.0051 at the 0.005 level.
(TIF)

S1 Table Total sample after quality control. The total number of individuals used for the meconium ileus association analysis after quality control of both genotypes and phenotypes, stratified by consortium site and Illumina genotyping platform.
(DOCX)

S2 Table SNP quality control steps. The number of SNPs before quality control (QC) and the number of SNPs excluded by each stated QC criterion, stratified by consortium sites and Illumina genotyping platform.
(DOCX)

S3 Table Comparison of meconium ileus association results between this study and the previous consortium GWAS. SNPs significant in either study (this study with n = 6770 and previous consortium GWAS published in Sun et al [10]) and of particular functional relevance are provided in this table.
(DOCX)

S4 Table Sex-specific association analysis of variants in *SLC6A14*. The three variants here include the top two ranked SNPs, rs3788766 and rs5905177 as in S3 Table, and putative functional variant rs12710568.
(DOCX)

S5 Table Parameter values for the simulation studies. Values of standardized true effect size of the associated SNP for GWAS or an eQTL used in the various simulation settings, with the corresponding GWAS or eQTL power of individually detecting the SNP at the 10^−8^ significance level, and the expected -log10(p-value) of the GWAS association or the eQTL analysis if the observed signal strength is the true effect size. See S1 Appendix for other simulation details.
(DOCX)

S6 TableOverview and illustration of the cases under the composite null hypothesis that there is no colocalization.Plots (a)-(d) provide the general visualization of GWAS (red line) and eQTL (blue) patterns (on the -log10 p scale) in a region of interest (e.g. the *SLC6A14* locus). The value of $\lambda_{Z_{c}}$ represents the standardized true effect size of a GWAS associated variant, and $\lambda_{T_{c}}$ represents the true standardized effect size of an eQTL variant. The corresponding power of detecting the GWAS SNP or finding the eQTL are provided in S5 Table. For illustration purposes but without loss generality, if there was a GWAS association, $\lambda_{Z_{c}}$ was set to be 5.73 such that 0.5 power could be achieved to detect the signal. If there was an eQTL from the gene-expression study, $\lambda_{T_{c}}$ was set to be 7.01 such that 0.9 power could be achieved to detect the signal. See S1 Appendix for other simulation details.(DOCX)

S7 Table Overview with illustration of the cases under different types of alternatives when there is colocalization at at least one variant in the region. Plots (a)-(f) provide the general visualization of GWAS (red line) and eQTL (blue) patterns (on the -log10 p scale) in a region of interest (e.g. the *SLC6A14* locus). The parameter values are varied in the power study, thus they are provided in the corresponding referred tables.
(DOCX)

S8 Table Type 1 error evaluation of the proposed Simple Sum colocalization analytical method, and the false positive rate of COLOC and eCAVIAR under the different null cases. The null cases considered are detailed in S6 Table. The LD pattern at the simulated region follows that at the *SLC6A14* locus. For the SS method, the nominal type 1 error was set at alpha = 0.05 or alpha = 0.005. The eQTL evidence was measured continuously as -log10 (eQTL p-value), or dichotomized using the eQTL p<0.05 or <0.005 threshold. For COLOC and eCAVIAR, the false positive rates were calculated by applying the 0.5, 0.75 or 0.9 threshold (as in [1]) to the colocalization posterior probability. In total, 10^4^ replications were simulated to obtain each cell of the table. See S1 Appendix for other simulation details.
(DOCX)

S9 Table Type 1 error evaluation of the proposed Simple Sum colocalization analytical method under the null Case 3. All the null cases considered are detailed in S6 Table. Case 3 is the situation when there is a GWAS associated SNP but there is no eQTL (plot (c) in S6 Table). In that case, colocalization results should be interpreted with caution if the observed eQTL signal is weak with examples demonstrated below (i.e. the null hypothesis is not rejected if the maximum of -log10 (eQTL p-value) is below a threshold (column 1)). Among the remaining replicates, the null is rejected if the Simple Sum colocalization p-value is smaller than the nominal type 1 error level (alpha = 0.05 or alpha = 0.005). The LD pattern at the simulated region follows that at the *SLC6A14* locus. In total, 10^4^ replications were simulated to obtain each cell of the table. See S1 Appendix for other simulation details.
(DOCX)

S10 Table Results of Simple Sum colocalization and contrasting colocalization analyses for the three loci genome-wide significantly associated with meconium ileus. The eQTL evidence was dichotomized by using thresholds of eQTL p<0.05, <0.005 or <0.0005 instead of based on the -log10(p-value) as in Table 3. Analytical and permutation-based (# of replicates = 10^5^) Simple Sum *(SS)* colocalization p-values evaluate if the eQTLs for a given gene and tissue colocalize with meconium ileus-associated variants. All colocalization p-values were one-sided because only positive association implies eQTL-association colocalization (i.e. eQTL peaks coincide with association peaks). Simple Sum Contrasting (*SSC*) colocalization p-value evaluates if the eQTLs in the pancreas colocalize with meconium ileus-associated variants more than eQTLs in another tissue; NAs are listed for the pancreas since we do not contrast pancreas with itself. Other NAs are used when there are no SNPs with eQTLs p less than the thresholds considered (0.05, 0.005 or 0.0005) for that gene and tissue.
(DOCX)

S11 TablePower evaluation of the proposed Simple Sum colocalization analytical method, and the true positive rate of COLOC and eCAVIAR, under the alternative that ONE GWAS and ONE eQTL locus colocolizes (Alter1 in S7 Table).The LD pattern at the simulated region follows that at the *SLC6A14* locus. For the SS method, the nominal type 1 error was set at alpha = 0.05 or alpha = 0.005. The eQTL evidence was measured continuously as -log10 (eQTL p-value), or dichotomized using the eQTL p<0.05 or <0.005 threshold. For COLOC and eCAVIAR, the false positive rates were calculated by applying the 0.5, 0.75 or 0.9 threshold to the colocalization posterior probability. The value of $\lambda_{Z_{c}}$ represents the standardized true effect size of the GWAS associated variant, and $\lambda_{T_{c}}$ represents the standardized true effect size of the eQTL variant, as detailed in S5 Table. Here, $\lambda_{Z_{c}}$ is set to be 5.73 such that 0.5 power is achieved to detect the GWAS association at significance level of 10^−8^, while $\lambda_{T_{c}}$ is set to be 3.4, 4.09, 4.45, 5.21 or 5.73 for each row of the table such that 0.01, 0.05, 0.1, 0.3, or 0.5 power is achieved to detect the eQTL association at significance level 10^−8^. In total, 10^4^ replications were simulated to obtain each cell of the table. See S1 Appendix for other simulation details.(DOCX)

S12 TablePower evaluation of the proposed Simple Sum colocalization analytical method, and the true positive rate of COLOC and eCAVIAR, under the alternative that the eQTL peak overlapped with the higher GWAS peak (Alter2 in S7 Table).The LD pattern at the simulated region follows that at the *SLC6A14* locus. For the SS method, the nominal type 1 error was set at alpha = 0.05 or alpha = 0.005. The eQTL evidence was measured continuously as -log10 (eQTL p-value) or dichotomized using the eQTL p<0.05 or <0.005 threshold. For COLOC and eCAVIAR, the false positive rates were calculated by applying the 0.5, 0.75 or 0.9 threshold to the colocalization posterior probability. The values of $\lambda_{Z_{c1}}$ and $\lambda_{Z_{c2}}$ represent the standardized true effect sizes of two GWAS associated variants, while $\lambda_{T_{c1}}$ and $\lambda_{T_{c2}}$ represent the standardized true effect sizes of two eQTL variants. Here, $\lambda_{Z_{c1}}$ is set to be 6.57 and $\lambda_{Z_{c2}}$ is set to be 5.73 such that, respectively, 0.8 and 0.5 power are achieved to detect the two GWAS signals at significance level 10^−8^. $\lambda_{T_{c1}}$ is set to be 3.4, 4.09, 4.45, 5.21 or 5.73 for each row of the table such that 0.01, 0.05, 0.1, 0.3, or 0.5 power is achieved to detect the eQTL association at significance level of 10^−8^ and $\lambda_{T_{c2}}$ is set to be 0. In total, 10^4^ replications were simulated to obtain each cell of the table. See S1 Appendix for other simulation details.(DOCX)

S13 TablePower evaluation of the proposed Simple Sum colocalization analytical method, and the true positive rate of COLOC and eCAVIAR, under the alternative that the eQTL peak overlapped with the lower GWAS peak (Alter3 in S7 Table).The LD pattern at the simulated region follows that at the *SLC6A14* locus. For the SS method, the nominal type 1 error was set at alpha = 0.05 or alpha = 0.005. The eQTL evidence was measured continuously as -log10 (eQTL p-value) or dichotomized using the eQTL p<0.05 or <0.005 threshold. For COLOC and eCAVIAR, the false positive rates were calculated by applying the 0.5, 0.75 or 0.9 threshold to the colocalization posterior probability. The values of $\lambda_{Z_{c1}}$ and $\lambda_{Z_{c2}}$ represent the standardized true effect sizes of two GWAS associated variants, while $\lambda_{T_{c1}}$ and $\lambda_{T_{c2}}$ represent the standardized true effect sizes of two eQTL variants. Here, $\lambda_{Z_{c1}}$ is set to be 6.57 and $\lambda_{Z_{c2}}$ is set to be 5.73 such that 0.8 and 0.5 power are achieved to detect GWAS signals at significance level of 10^−8^. $\lambda_{T_{c1}}$ is set to be 0, and $\lambda_{T_{c2}}$ is set to be 3.4, 4.09, 4.45, 5.21 or 5.73 for each row of the table such that 0.01, 0.05, 0.1, 0.3, or 0.5 power is achieved to detect the eQTL association at significance level 10^−8^. In total, 10^4^ replications were simulated to obtain each cell of the table. See S1 Appendix for other simulation details.(DOCX)

S14 TablePower evaluation of the proposed Simple Sum colocalization analytical method, and the true positive rate of COLOC and eCAVIAR, under the alternative that the non-overlapped eQTL peak is lower than the GWAS peak (Alter4 in S7 Table).The LD pattern at the simulated region follows that at the *SLC6A14* locus. For the SS method, the nominal type 1 error was set at alpha = 0.05 or alpha = 0.005. The eQTL evidence was measured continuously as -log10 (eQTL p-value) or dichotomized using the eQTL p<0.05 or <0.005 threshold. For COLOC and eCAVIAR, the false positive rates were calculated by applying the 0.5, 0.75 or 0.9 threshold to the colocalization posterior probability. The values of $\lambda_{Z_{c1}}$ and $\lambda_{Z_{c2}}$ represent the standardized true effect sizes of two GWAS associated variants, while $\lambda_{T_{c1}}$ and $\lambda_{T_{c2}}$ represent standardized true effect sizes of two eQTL variants. Here, $\lambda_{Z_{c1}}$ is set to be 6.57 such that 0.8 power is achieved to detect that GWAS signal at significance level 10^−8^ and $\lambda_{Z_{c2}}$ is set to be 0. ${\lambda_{T}}_{c_{1}}$ is set to be 3.4, 4.09, 4.45, 5.21 or 5.73 for each row of the table such that 0.01, 0.05,0.1,0.3, or 0.5 power are achieved to detect the eQTL association at significance level of 10^−8^, and $\lambda_{T_{c2}}$ is set to be 5.73 such that 0.5 power is achieved to detect that eQTL signal at significance level of 10^−8^. In total, 10^4^ replications were simulated to obtain each cell of the table. See S1 Appendix for other simulation details.(DOCX)

S15 TablePower evaluation of the proposed Simple Sum colocalization analytical method, and the true positive rate of COLOC and eCAVIAR, under the alternative that the non-overlapped eQTL peak is higher than the GWAS peak (Alter5 in S7 Table).The LD pattern at the simulated region follows that at the *SLC6A14* locus. For the SS method, the nominal type 1 error was set at alpha = 0.05 or alpha = 0.005. The eQTL evidence was measured continuously as -log10 (eQTL p-value) or dichotomized using the eQTL p<0.05 or <0.005 threshold. For COLOC and eCAVIAR, the false positive rates were calculated by applying the 0.5, 0.75 or 0.9 threshold to the colocalization posterior probability. The values of $\lambda_{Z_{c1}}$ and $\lambda_{Z_{c2}}$ represent the standardized true effect sizes of two GWAS associated variants, while $\lambda_{T_{c1}}$ and $\lambda_{T_{c2}}$ represent the standardized true effect sizes of eQTL variants. Here, $\lambda_{Z_{c1}}$ is set to be 6.57 such that 0.8 power is achieved to detect that GWAS signal at significance level of 10^−8^ and $\lambda_{Z_{c2}}$ is set to be 0. ${\lambda_{T}}_{c_{1}}$ is set to be 3.4, 4.09, 4.45, 5.21 or 5.73 for each row of the table such that 0.01, 0.05,0.1,0.3, or 0.5 power are achieved to detect the eQTL association at significance level of 10^−8^ and $\lambda_{T_{c2}}$ is set to be 7.01 such that 0.9 power is achieved to detect that eQTL signal at significance level of 10^−8^. In total, 10^4^ replications were simulated to obtain each cell of the table. See S1 Appendix for other simulation details.(DOCX)

S16 TablePower evaluation of the proposed Simple Sum colocalization analytical method, and the true positive rate of COLOC and eCAVIAR, under the alternative that there are two overlapping GWAS and eQTL loci (Alter6 in S7 Table).The LD pattern at the simulated region follows that at the *SLC6A14* locus. For the SS method, the nominal type 1 error was set at alpha = 0.05 or alpha = 0.005. The eQTL evidence was measured continuously as -log10 (eQTL p-value) or dichotomized using the eQTL p<0.05 or <0.005 threshold. For COLOC and eCAVIAR, the false positive rates were calculated by applying the 0.5, 0.75 or 0.9 threshold to the colocalization posterior probability. The values of $\lambda_{Z_{c1}}$ and $\lambda_{Z_{c2}}$ represent the standardized true effect sizes of two GWAS associated variants, while $\lambda_{T_{c1}}$ and $\lambda_{T_{c2}}$ represent the standardized true effect sizes of eQTL variants. Here, $\lambda_{Z_{c1}}$ is set to be 6.57 and $\lambda_{Z_{c2}}$ is set to be 5.73 such that, respectively, 0.8 and 0.5 power are achieved to detect the two GWAS signals at significance level of 10^−8^. ${\lambda_{T}}_{c_{1}}$ is set to be 3.4, 4.09, 4.45, 5.21 or 5.73 for each row of the table such that 0.01, 0.05,0.1,0.3, or 0.5 power are achieved to detect the eQTL association at significance level of 10^−8^ and $\lambda_{T_{c2}}$ is set to be 7.01 such that 0.9 power is achieved to detect that eQTL signal at significance level of 10^−8^. In total, 10^4^ replications were simulated to obtain each cell of the table. See S1 Appendix for other simulation details.(DOCX)

S17 Table Results of Simple Sum colocalization and contrasting colocalization analyses for genes at the Chromosome X locus in the region including 0.1Mbp on either side of the lead SNP in CF human nasal epithelial and lung from GTEx. The exact region is 115248275–115448275 bp as in human genome reference assembly GRCh37. The eQTL evidence used include the -log_10_ transform of eQTL p value, and dichotomized eQTL p-value indicator by thresholds of eQTL p<0.05 or <0.005 for each specified gene and tissue. We focus on the analysis evaluating whether the eQTLs for *SLC6A14* in lung (or human nasal epithelial; HNE) colocalize with lung-associated variants more than eQTLs for *AGTR2*, *PLS3* and *CXorf61* in lung (or HNE). The contrasting colocalization test for *SLC6A14* is listed as NA since we do not contrast *SLC6A14* with itself; NA in other cells means no eQTL SNP with p<0.05 or p<0.005 for that gene. The column ‘No. of eQTL SNPs’ shows the number of SNPs with eQTL p-values< 0.05 or 0.005 in the 0.1Mbp region; it refers to the number of SNPs for which eQTL p-values were available at the locus when -log_10_(eQTLp) is used (first 4 rows of the table). All p-values are one-sided to ensure colocalization rather than negative correlation.
(DOCX)
