# Supplementary material for: Genetic association and transcriptome integration identify contributing genes and tissues at cystic fibrosis modifier loci
Source: PLoS Genet. 2019 Feb 26;15(2):e1008007. doi: 10.1371/journal.pgen.1008007 (PMC6407791; doi:10.1371/journal.pgen.1008007)
Supplement: S1 Appendix — (DOCX) [file pgen.1008007.s001.docx]

**Supporting Information**

# S1 Appendix: The Simple Sum (*SS*) analytical framework and method evaluation and comparisons

At each genomic locus of interest, to statistically determine whether the phenotype-SNP associations were mediated through gene expression, and in which tissue, we developed a colocalization framework, the Simple Sum (*SS*). *SS* is a robust GWAS and regulatory element integration method that uses only summary statistics, allows for allelic heterogeneity, and does not require permutation to assess statistical significance.

Here we provide the analytical details of the *SS* method. We first describe the two components to be integrated, namely, the phenotype-SNP association component and the gene expression-SNP association component, although this could be any SNP-level information. We then present the *SS* colocalization test for a single tissue (or gene). To determine if there is better colocalization with one tissue (or gene) than another tissue (or gene), we propose the *SS* contrast colocalization test, contrasting two tissues (or genes). Finally, we describe the simulation studies conducted to evaluate the performance of the method in terms of type 1 error control and power, and compare it with COLOC [1] and eCAVIAR [2], two alternative Bayesian approaches.

**The Phenotype-SNP association component**

Let ***Y*** denote the phenotype under study, where ***Y*** is the binary meconium ileus status in our analysis but can be continuous trait outcomes in other settings. Let $\boldsymbol{X}_{\boldsymbol{j}}$ be the genotype of SNP *j* for each of the *j = 1, . . ., m* SNPs from a region or locus of interest (e.g. the chromosome X locus around *SLC6A14*). Assume that there are *n* individuals, the phenotype vector and genotype matrix are ***Y*** and *X*, respectively, where

$\mathbf{Y}=\binom{\begin{aligned} y_{1} \\ \vdots\end{aligned}}{\begin{aligned} y_{i} \\ \vdots\\ y_{n} \end{aligned}}$ *,* $X=\left( \begin{matrix} \begin{matrix} x_{11} & \cdots& x_{1j} \\ \vdots& \cdots& \vdots\\ x_{i1} & \cdots& x_{ij} \end{matrix} & \begin{matrix} \cdots& x_{1m} \\ \cdots& \vdots\\ \cdots& x_{im} \end{matrix} \\ \begin{matrix} \vdots& \cdots& \vdots\\ x_{n1} & \cdots& x_{nj} \end{matrix} & \begin{matrix} \cdots& \vdots\\ \cdots& x_{nm} \end{matrix} \end{matrix} \right)=\left( \boldsymbol{X}_{\boldsymbol{1}}\boldsymbol{,\ldots,}\boldsymbol{X}_{\boldsymbol{j}}\boldsymbol{, \ldots,}\boldsymbol{X}_{\boldsymbol{m}} \right).$

Let $\boldsymbol{Z}=(Z_{1},\ldots Z_{j},\ldots,Z_{m})$ be the vector containing the phenotype-SNP association statistics for the *m* SNPs. As in convention, ***Z*** is multivariate normal with covariance matrix $\Sigma$ that captures the linkage disequilibrium (LD) in the region of interest. Since the direction of effect for a SNP depends on the choice of the reference allele, we thus use $S_{j}=Z_{j}^{2}$ as a measure of association evidence, where *Sj* represents the Wald-type $\chi_{1}^{2}$ test statistic for the *jth* SNP, and the vector of phenotype-SNP association statistics for the *m* SNPs is denoted as

$\boldsymbol{S} =\left（ \begin{matrix} \begin{matrix} S_{1} \\ \vdots\end{matrix} \\ \begin{matrix} S_{j} \\ \begin{matrix} \vdots\\ S_{m} \end{matrix} \end{matrix} \end{matrix} \right）=\left( S_{1}, \ldots,S_{j},\ldots, S_{m} \right)^{'}$.

For the proposed *SS* colocalization method, only the summary statistics ***Z*** are needed. The covariance matrix$\Sigma$, if not available, can be estimated from publicly available data such as the 1000 Genomes Project [3], provided the correct population reference sample was used.

For a gene of interest (e.g. *SLC6A14*), we assume that expression quantitative trait loci (eQTLs) p-values (or test statistics) were available, for example from the genotype tissue expression project (GTEx, [4]),

$$T=\left( \begin{matrix} \begin{matrix} t_{11} & \cdots& t_{1k} \\ \vdots& \cdots& \vdots\\ t_{j1} & \cdots& t_{jk} \end{matrix} & \begin{matrix} \cdots& t_{1K} \\ \cdots& \vdots\\ \cdots& t_{jK} \end{matrix} \\ \begin{matrix} \vdots& \cdots& \vdots\\ t_{m1} & \cdots& t_{mk} \end{matrix} & \begin{matrix} \cdots& \vdots\\ \cdots& t_{mK} \end{matrix} \end{matrix} \right)=\left( \boldsymbol{t}_{\boldsymbol{1}},\ldots,\boldsymbol{t}_{\boldsymbol{k}},\ldots,\boldsymbol{t}_{\boldsymbol{K}} \right),$$

$$\boldsymbol{t}_{k}=\left( t_{1k}, \ldots,t_{jk},\ldots,t_{mk} \right)^{'},$$

where $t_{jk}$ is the p-value of the gene expression-SNP association between gene expression in tissue *k*, *k* = 1, . . ., *K* (e.g. pancreas and small intestines) and SNP *j,* *j* = 1, . . ., *m*. Since we are interested in small p-values, we consider the gene expression p-values on the –log_10_ scale, that is $t_{jk}=$ –log_10_(eQTL p). Alternatively, we can dichotomize the p-values using a pre-specified criterion, e.g. if eQTL p<0.05, $t_{jk}$=1; otherwise $t_{jk}$=0. Obviously, the criterion is subjective, and eQTL p<0.005 or even 0.0005 can also be considered for comparison. Thus, $t_{jk}$is either log_10_(eQTL p) or a binary eQTL indicator variable for SNP *j* and the gene of interest in tissue *k*; we will make the meaning clear depending on the context.

Note that for a given tissue of interest (e.g. lung), *k* could also denote eQTLs for different genes (e.g. *SLC6A14* and *AGTR2*) as in S17 Table.

**The *SS* colocalization test for a single tissue (or gene)**

For a locus of interest, here we assume that what is available to us are the summary statistics for the two components: phenotype-SNP association statistics stored in vector ***S*** for the *m* SNPs in the region, and gene expression-SNP association p-values stored in matrix *T* for the *m* SNPs and *K* tissues (or genes) of interest.

Focusing on one specific gene (e.g. *SLC6A14*) and tissue *k* (e.g. SLC6A14 expression in the pancreas), we are interested in whether larger phenotype-SNP association evidence occurs on average when there is larger eQTL evidence. Thus, it is natural to consider the following *Simple Sum* (*SS*) statistic:

$$SS=\frac{1}{\sum_{j} I\left( t_{jk}=1 \right)}\sum_{j} S_{j}I\left( t_{jk}=1 \right)-\frac{1}{\sum_{j} I\left( t_{jk}=0 \right)}\sum_{j} S_{j}I\left( t_{jk}=0 \right).$$

where *j* = 1, . . ., *m*. The p-value for the *SS* test can be evaluated by standard permutation procedure, but this is computationally costly. Alternatively, we derived the exact distribution of the *SS* statistic by expressing *SS* in a quadratic form. To this end, a simple algebraic manipulation can show that

$SS=\frac{\sum_{j} S_{j}t_{jk}}{\sum_{j} t_{jk}}-\frac{\sum_{j} S_{j}\left( 1-t_{jk} \right)}{\sum_{j} \left( 1-t_{jk} \right)}=\frac{\sum_{j} S_{j}t_{jk}-m\bar{S}\bar{t}_{k}}{\sum_{j} t_{jk}-m\bar{t}_{k}^{2}}$*,*

where $\bar{S}=\sum_{j} S_{j}/m$ and $\bar{t}_{k}=\sum_{j} t_{jk}/m$. Note that $\sum_{j} t_{jk}=\sum_{j} t_{jk}^{2}$when $\boldsymbol{t}_{\boldsymbol{k}}$ is binary. Thus,

$$SS=\frac{\sum_{j} S_{j}t_{jk}-m\bar{S}\bar{t}_{k}}{\sum_{j} t_{jk}^{2}-m\bar{t}_{k}^{2}}=\frac{\sum_{j} S_{j}t_{jk}-\sum_{j} S_{j}\bar{t}_{k}}{\sum_{j} t_{jk}^{2}-m\bar{t}_{k}^{2}}$$

$=\sum_{j} S_{j} (\frac{t_{jk}-\bar{t_{k}}}{\sum_{j} t_{jk}^{2}-m\bar{t}_{k}^{2}})=\sum_{j} Z_{j}^{2} (\frac{t_{jk}-\bar{t_{k}}}{\sum_{j} t_{jk}^{2}-m\bar{t}_{k}^{2}})=\boldsymbol{Z}'A\boldsymbol{Z}$,

where $A=diag\left( a_{1},a_{2},\ldots,a_{m} \right)$ with $a_{j}=\frac{t_{jk}-\bar{t_{k}}}{\sum_{j} t_{jk}^{2}-m\bar{t}_{k}^{2}}$and $j=1,2,\ldots,m$.

First, consider the simple null hypothesis of no association and no-colocalization. In that case, $\boldsymbol{Z}\sim N\left( 0,\Sigma\right)$ and $\boldsymbol{Z}^{'}A\boldsymbol{Z}=\left( \Sigma^{-\frac{1}{2}}Z \right)^{'}\left( \Sigma^{\frac{1}{2}} \right)^{'}A\Sigma^{\frac{1}{2}}\left( \Sigma^{-\frac{1}{2}}Z \right).$Because $\left( \Sigma^{\frac{1}{2}} \right)^{'}A\Sigma^{\frac{1}{2}}$ is a symmetric matrix, we can carry out a spectral decomposition and $\left( \Sigma^{\frac{1}{2}} \right)^{'}A\Sigma^{\frac{1}{2}}= PDP'$, where *P* is an orthogonal matrix with columns that are eigenvectors of $\left( \Sigma^{\frac{1}{2}} \right)^{'}A\Sigma^{\frac{1}{2}}$and *D* is a diagonal matrix with diagonal elements that are corresponding eigenvalues, $D=diag\left( d_{1},\ldots d_{m} \right)$. Therefore,

$$\boldsymbol{Z}^{'}A\boldsymbol{Z}=\left( \Sigma^{-\frac{1}{2}}Z \right)'PDP'\left( \Sigma^{-\frac{1}{2}}Z \right)=\left( P{'\Sigma}^{-\frac{1}{2}}Z \right)^{'}D\left( P{'\Sigma}^{-\frac{1}{2}}Z \right).$$

Note that under this simple null hypothesis, $P'\Sigma^{-\frac{1}{2}}Z\sim N(0, P'IP)$, thus $P'\Sigma^{-\frac{1}{2}}Z\sim N(0, I)$ and

$$SS=(P{'\Sigma}^{-\frac{1}{2}}Z)'D(P{'\Sigma}^{-\frac{1}{2}}Z)=\sum_{j} d_{j}\chi_{1}^{2}.$$

Therefore, the *SS* colocalization p-value can be obtained without the need for simulation. In addition, this analytic form allows for use of a continuous measure of gene expression evidence (that is,$t_{jk}=$ –log_10_(eQTL p) instead of the 0-1 binary indicator) in the *SS* statistic, making the subjective choice of eQTL cut-off (e.g. eQTL p<0.05) unnecessary. The accuracy of this method was evaluated via simulation studies (see subsection below and S8-S13 Figs and S8-S9 Tables), and was also validated by the similarity between the empirical p-values calculated through permutation and the analytical p-values in applications (Table 3 and S10 Table).

Several important remarks need to be made. First, we note that although the proposed *SS* method intuitively only uses the difference in the sum of association test statistics for eQTL and non-eQTL SNPs, it inherently adjusts for LD between SNPs by properly calibrating the variance of the *SS* test statistic through $\Sigma$ that contains the LD pattern in the region of interest.

Second, the test must be *one-sided.* Since we are interested in whether larger phenotype-SNP association evidence occurs on average when there is larger eQTL evidence, intuitively only a *positive* test statistic suggests colocalization of association and eQTL evidence. Importantly, the one-sided test deals with the complex issue of a composite null inherent in colocalization studies.

Now consider the alternative of interest, which is that there is at least one GWAS signal and at least one eQTL signal and that the two coincide. The complement to this alternative includes the simple null considered above, but also other types of null such as the GWAS and eQTL signals occurring at two independent SNPs; this would be the H3 scenario considered by COLOC [1]. In that case, a large *tjk* corresponds to a small $S_{j}$, and it is easy to see that the *SS* test statistic would be negative, resulting in a large p-value if a one-sided test was performed. Thus, although the asymptotic distribution of the *SS* statistic was derived under the simple null, under this composite null the one-sided test guarantees the type 1 error rate will be well controlled below the nominal level. Simulation studies provide empirical corroborating evidence (Case 4 simulation design in S6 Table with corresponding type 1 error results in S8 Table).

The composite null also includes the situation when there is no association but the eQTL is significant (Case 2 simulation design in S6 Table), and the situation when there is association but no eQTL (Case 3 simulation design in S6 Table). Type 1 error is controlled for Case 2 (S8 Table), because the asymptotic distribution of the *SS* statistic was derived conditional on eQTL p-values via matrix $A$ which is $\mathrm{diag}\left( a_{1},a_{2},\ldots,a_{m} \right)$ with $a_{j}=\frac{t_{jk}-\bar{t_{k}}}{\sum_{j} t_{jk}^{2}-m\bar{t}_{k}^{2}}$ that inherently centralizes any eQTL signals. However, for Case 3 (association but no eQTL), type 1 error is not assured to be controlled when using –log_10_(eQTL p) or liberal eQTL cut-off criteria (e.g eQTL p <0.05). Specifically, the empirical type 1 error could be inflated (Case 3 in S8 Table) when the LD in the region of interest is extensive and colocalization analysis is computed despite eQTL p-values being large or eQTL evidence being weak. This is because any small peak of eQTL evidence occurring by chance will propagate to the rest of the region via LD, and could by chance overlap with the association SNP. Fortunately, we are aware of this situation in which type 1 error can be inflated and can use caution when interpreting colocalization findings in regions with low eQTL evidence. We conducted simulation studies to show type 1 error rate control for Case 3 when requiring the maximum eQTL evidence in the region to be at certain level (e.g. -log10(eQTL p-value) > 3, S9 Table). Results demonstrate that this is an effective strategy (S9 Table). It is important to note that in our application study the eQTL evidence at the three genome-wide significant loci (Fig 2) all exceed the p-values at which type 1 error inflation has been observed.

**The SS contrast colocalization test, contrasting two tissues (or genes)**

To determine if colocalization in tissue *k* (or gene) is significantly more so than in tissue *h* (or gene), we want to contrast the colocalization evidence between two tissues (or two genes). So, it is natural to consider the following statistic:

$SSC=\frac{1}{\sum_{j} I\left( t_{jk}=1 \right)}\sum_{j} S_{j}I\left( t_{jk}=1 \right)-\frac{1}{\sum_{j} I\left( t_{jh}=1 \right)}\sum_{j} S_{j}I\left( t_{jh}=1 \right)$,

where $t_{jk}$ and $t_{jh}$ are the gene-expression indicator variable (e.g. eQTL p<0.05 or 0.005) for SNP *j* in tissue *k* and *h*, respectively.

Again, to obtain p-values for *SSC* efficiently without permutations and to use the original continuous measure of gene expression evidence for an eQTL, we show that the above *SSC* test statistic can be reformulated in a quadratic form that is also distributed as a mixture of chi-squared distributions under the simple null.

To reformulate the *SSC* statistic above, where the $t_{jk}$and $t_{jh}$ are eQTL indicator variables for tissues *k* and *h*, we first separate the *m* SNPs from the region of interest into four sets. Let $I_{1}$ represent the set of SNPs that are eQTLs for tissue *k* but not for tissue *h* (i.e. $t_{jk}=1$ and $t_{jh}=0),$ $I_{2}$be the set of SNPs with $t_{jk}=0$ and $t_{jh}=1$, $I_{3}$be the set of SNPs with $t_{jk}=0$ and $t_{jh}=0$, $I_{4}$be the set of SNPs with $t_{jk}=1$ and $t_{jh}=1$, and let $m_{i}$be the number of SNPs in set $I_{i}$, i=1, 2, 3, and 4.

Let $\boldsymbol{t}_{\boldsymbol{k}}^{\boldsymbol{*}}$ be a vector of length ($m-m_{3})$with components from $\boldsymbol{t}_{\boldsymbol{k}}$ except the ones corresponding to SNPs from set $I_{3}$ (i.e. excluding the SNPs that are not eQTL for either tissue *k* or *h*); and similarly define$\boldsymbol{t}_{\boldsymbol{h}}^{\boldsymbol{*}}$ and ***S****=***Z****^2^. Then,

$SSC=\frac{1}{\sum_{j} I\left( t_{jk}^{*}=1 \right)}\sum S_{j}^{*}I\left( t_{jk}^{*}=1 \right)-\frac{1}{\sum_{j} I\left( t_{jh}^{*}=1 \right)}\sum S_{j}^{*}I\left( t_{jh}^{*}=1 \right)$

$=\frac{\sum_{j} S_{j}^{*}t_{jk}^{*}}{\sum_{j} t_{jk}^{*}}-\frac{\sum_{j} S_{j}^{*}t_{jh}^{*}}{\sum_{j} t_{jh}^{*}}$ $=\sum_{j} S_{j}^{*} (\frac{t_{jk}^{*}}{\sum_{j} t_{jk}^{*}}-\frac{t_{jh}^{*}}{\sum_{j} t_{jh}^{*}})$

$=\sum_{j} Z_{j}^{*2} (\frac{t_{jk}^{*}}{\sum_{j} t_{jk}^{*}}-\frac{t_{jh}^{*}}{\sum_{j} t_{jh}^{*}})$

$={Z^{*}}^{'}BZ^{*}$,

where $B=diag\left( b_{1},b_{2},\ldots,b_{m} \right)$ with $b_{j}=\frac{t_{jk}^{*}}{\sum_{j} t_{jk}^{*}}-\frac{t_{jh}^{*}}{\sum_{j} t_{jh}^{*}}$ and $j=1,2,\ldots,m$.

The analytical derivation is similar to that for the SS colocalization test. That is, under the null hypothesis, $Z^{*}\sim N\left( 0,\Sigma^{*} \right),$ where $\Sigma^{*}$captures the LD among those (*m-m_3_*) SNPs in the region of interest.

${Z^{*}}^{'}BZ^{*}=\left( {\Sigma^{*}}^{-\frac{1}{2}}Z^{*} \right)^{'}\left( {\Sigma^{*}}^{\frac{1}{2}} \right)^{'}B{\Sigma^{*}}^{\frac{1}{2}}\left( {\Sigma^{*}}^{-\frac{1}{2}}Z^{*} \right)$*.*

Due to the nature of $\boldsymbol{t}_{k}^{*}$ and $\boldsymbol{t}_{h}^{*}$, B is a full rank diagonal matrix and ($\left( {\Sigma^{*}}^{\frac{1}{2}} \right)^{'}B{\Sigma^{*}}^{\frac{1}{2}})'=\left( {\Sigma^{*}}^{\frac{1}{2}} \right)^{'}B{\Sigma^{*}}^{\frac{1}{2}}$, so $\left( {\Sigma^{*}}^{\frac{1}{2}} \right)^{'}B{\Sigma^{*}}^{\frac{1}{2}}$is also a full rank and symmetric matrix. Spectral decomposition leads to $\left( {\Sigma^{*}}^{\frac{1}{2}} \right)^{'}B{\Sigma^{*}}^{\frac{1}{2}}=FLF'$, where *F* is an orthogonal matrix with columns that are eigenvectors of $\left( {\Sigma^{*}}^{\frac{1}{2}} \right)^{'}B{\Sigma^{*}}^{\frac{1}{2}}$, and *L* is a diagonal matrix with diagonal elements that are corresponding eigenvalues, $L=diag\left( l_{1},\ldots l_{m-m_{3}} \right)$. Therefore,

${Z^{*}}^{'}BZ^{*}=\left( {\Sigma^{*}}^{-\frac{1}{2}}Z^{*} \right)'FLF'\left( {\Sigma^{*}}^{-\frac{1}{2}}Z^{*} \right)=(F'{\Sigma^{*}}^{-\frac{1}{2}}Z^{*})'L(F'{\Sigma^{*}}^{-\frac{1}{2}}Z^{*})$*.*

Note that under the null $F'{\Sigma^{*}}^{-\frac{1}{2}}Z^{*}\sim N(0, F'IF)$, thus $F'{\Sigma^{*}}^{-\frac{1}{2}}Z^{*}\sim N(0, I)$ and

$SSC=(F'{\Sigma^{*}}^{-\frac{1}{2}}Z^{*})'L(F'{\Sigma^{*}}^{-\frac{1}{2}}Z^{*})=\sum_{j} l_{j}\chi_{1}^{2}$*_._*

Therefore, the *SSC* colocalization p-value can be obtained without simulation; p-values can be calculated by using the R package CompQuadForm.

A technical detail should be noted for the implementation of the mixture of chi-squared distributions. The proof of the exact distribution assumes that $\Sigma$is invertible so $\Sigma^{-\frac{1}{2}}$ exists. When two SNPs are highly correlated, i.e. in strong LD, *Σ* may not be invertible. In that case, we use a ridge-type adjustment and replace $\Sigma$ with $\Sigma+\lambda I$, where *λ* used is selected via 10-fold cross-validation. Specifically, the createFolds function in R was used to randomly separate the observations into ten groups of equal size, *i* = 1, . . ., 10, and $\Sigma_{i}$represents the sample variance-covariance matrix for the $i_{th}$group, and Σ-i for the remaining nine groups. The chosen λ was the value that minimized $\sum_{i=1}^{10} |\left( \Sigma_{-i}+\lambda I \right)-\Sigma_{i}|$ where the F. norm2 R function was used to calculate the norm.

**Simulation Study**

To evaluate the performance of the proposed *SS* colocalization framework we conducted extensive simulation studies based on the LD pattern observed at the *SLC6A14* locus. For method comparisons with alternatives (which are Bayesian), we focused on COLOC [1] and eCAVIAR [2] based on comparative studies in the literature [5] (Material and Methods).

As in [2] , we considered all SNPs in the region of 0.1mb on either side of the lead SNP to generate each locus. GWAS summary statistics for the locus, 𝑍 = (𝑍_1_, 𝑍_2_, …, 𝑍_𝑚_)’, were generated from ***N*** (Σ$\Lambda_{z}$, Σ), and eQTL summary statistics were generated from ***N*** (Σ$\Lambda_{T}$, Σ) then converted to eQTL p-values, where Σ is the LD matrix for the locus of interest, i.e. the *SLC6A14* locus. $\Lambda_{z}=(\lambda_{z1},$ $\lambda_{z2}, \ldots,\lambda_{zm})'$and $\Lambda_{T}=(\lambda_{T1},$ $\lambda_{T2}, \ldots,\lambda_{Tm})'$ are vectors with each component being the standardized true effect size of the corresponding SNP, respectively for GWAS and eQTL analyses, where $\lambda_{Z_{c}}$is the value for the most significant GWAS SNP (or the lead GWAS SNP) while $\lambda_{T_{c}}$ is the value for the most significant eQTL (or the lead eQTL SNP). We will set different values for $\lambda_{Z_{c}}$and $\lambda_{T_{c}}$for studies of type 1 error and power (S5 Table). For each scenario considered, a total of 10^4^ replications were simulated.

*Type 1 error*

For type 1 error evaluation, we considered different null cases where Case 1 represents the simple null of no association and no eQTL (i.e. $\lambda_{Z_{c}}=0$ and $\lambda_{T_{c}}=0$) while Cases 2-4 correspond to the composite null scenarios where for example both the GWAS lead SNP and eQTL lead SNP are non-zero (e.g. $\lambda_{Z_{c}}$= 5.73 and $\lambda_{T_{c}}=$7.01 of Case 4 in S6 Table) but are independent of each other. We provide detailed descriptions and illustrations of the four cases in S6 Table.

For Case 1, in addition to the LD pattern at *SLC6A14,* we also considered the *SLC26A9* and *ATP12A* loci. S8, S10 and S12 Figs demonstrate type 1 error control of the *SS* test when data were simulated based on the different LD patterns at the *SLC6A14, SLC26A9* and *ATP12A* loci, respectively, and when the eQTL evidence was measured as –log10(eQTL p). To be comprehensive, S9, S11 and S13 Figs provide the corresponding type 1 error control results when eQTL p-values were dichotomized using a threshold of p<0.05 when calculating the SS test statistic. Similarly, S15-S20 Figs provide evidence for good type 1 error control of the *SSC* contrasting test, for the three loci and using either continuous or dichotomized measures of the eQTL evidence.

Without loss of generality, for the other scenarios we focused on using the LD pattern at the *SLC6A14* locus and S8 and S9 Tables provide the empirical type 1 error rate of the *SS* method. As discussed earlier, for Case 3 (association but no eQTL), the empirical type 1 error could be inflated when the LD in the region of interest is extensive and colocalization is calculated despite large eQTL p-values (Case 3 in S8 Table). In our simulation studies, we investigated type 1 error rate control when requiring larger -log10(eQTL p-value) for colocalization calculation in Case 3 (S9 Table), and showed that this is a pragmatic approach. It is of particular note that there is type 1 error rate control at the eQTL values observed in our application data at the *SLC26A9, SLC6A14* and *ATP12A* loci.

There are several colocalization methods in the literature [1, 2, 5, 6], the majority being Bayesian, with ENLOC, eCAVIAR and COLOC being amenable to analyses with summary statistics. The proposed frequentist *SS* framework complements existing approaches. A recent study [5] compared these Bayesian approaches and concluded eCAVIAR to be conservative with low false positive rate and low power compared to ENLOC, while COLOC was a special case of ENLOC and agreed in the presence of strong signals. We conducted a comprehensive comparison between the *SS*, COLOC and eCAVIAR methods through simulations. We calculated the false positive rates of COLOC and eCAVIAR using three different cut-off values (0.5,0.75 and 0.9) for the posterior probability as suggested in [1].

Of the colocalization methods, only the proposed contrast colocalization test in our *SS* framework formally tests whether colocalization is greater for a given tissue or gene. Here, similar to the single-tissue (or gene) test above, we conducted simulation studies based on the LD patterns observed at the three loci of interest: *SLC6A14, ATP12A and SLC26A9*. We simulated two sets of independent eQTL summary statistics from ***N*** (Σ$\Lambda_{T}$, Σ) corresponding to eQTL analyses of two tissues (or two genes) of interest, in addition to GWAS 𝑍 from ***N*** (Σ$\Lambda_{z}$, Σ). Focusing on the simple null of no association and no eQTL, $\lambda_{Z_{c}}=0$ for GWAS and $\lambda_{T_{c}}=0$ for eQTL in both tissues. We provide the corresponding type 1 error control of the *SSC* contrasting test in S15-S20 Figs, for the three loci and using either continuous or dichotomized measures of the eQTL evidence.

*Power*

For power evaluation, we also focused on the *SLC6A14* region but considered a variety of six alternatives including allelic heterogeneity, in addition to the simple case where one single GWAS lead SNP colocalizes with one single eQTL lead SNP. For example, there might be two eQTLs but only one of the two colocalizes with a GWAS signal, and vice versa. We provide a detailed description with illustrations of the cases considered in S7 Table, and the corresponding power results in S11-16 Tables alongside the true positive rate of COLOC and eCAVIAR.

Here, we let $\lambda_{Z_{c1}}$ and $\lambda_{Z_{c2}}$ represent the standardized true effect sizes of the first and second GWAS lead SNPs, and we let $\lambda_{T_{c1}}$ and $\lambda_{T_{c2}}$represent the standardized true effect sizes of the first and second eQTL lead SNPs. We set different values for $\lambda_{Z_{c1}},$ $\lambda_{Z_{c2}}, \lambda_{T_{c1}}$, and $\lambda_{T_{c2}}$to generate summary statistics under six alternative situations (S7 Table). For scenarios where there are two association peaks, we used the lead SNP for the GWAS study of meconium ileus at this locus (with substantial LD) to be the first lead SNP, and defined the second lead SNP at a different locus (with lesser LD) as the next adjacent SNP with r <0.002 and >-0.002 with the first lead SNP.

# Supplemental Figures

**S1 Fig.** **Pairwise comparison of the first three principal components from a PCA analysis of all CF samples together with the reference samples from the International Hapmap consortium.** Different solid dots show the clusters of the samples from the International Hapmap consortium [7]. The red circles correspond to our samples with outliers highlighted as black dots; outliers defined as 6 S.D. away from the center of the HapMap3 European (CEU/TSI) cluster. See the detailed description of the Hapmap samples at <http://www.sanger.ac.uk/resources/downloads/human/hapmap3.html>.

**S2 Fig.** **Comparison of CFTR-regional association with meconium ileus between two different imputation references.** The Locus zoom plot [8] of CFTR association imputed using (A) the 1000 Genome Project Phase 3 [3], and (B) the hybrid reference (augmenting the 1000 genome reference with the whole genome sequencing from 101 patients with CF). Imputed variants with MAF>1% were analyzed.

**S3 Fig. Locus Zoom plot of meconium ileus association in a 200 kb region surrounding *ATP12A* in individuals homozygous for Phe508del.**

**S4 Fig. Conditional association analysis of the SNPs from the three genome-wide significant loci for meconium ileus.** All subfigures are plotted in 200kb regions surrounding (A) *SLC6A14*, (B) *SLC26A9*, and (C) *ATP12A*. The GWAS signals are obliterated after conditioning on the top SNP in each region. The color of each dot represents the amount of LD of the SNP to the purple diamond point, which is the top SNP in each region after the conditional analysis.

**S5 Fig. Locus zoom plot of meconium ileus association in a 200 kb region surrounding *PRSS1* in the GWAS using the whole GMC sample of 6,770 individuals with CF.**

**S6 Fig. *SLC6A14* sex-stratified eQTLs in the pancreas show co-localization of eQTLs in males, but not females.** GTEx expression data was analyzed separately by sex for eQTL association with *SLC6A14* in the pancreas using the same linear regression model described in Materials and Methods, GTEx Data without the sex covariate. Dots in the figure represent the association with MI, while the lines depict the association pattern of eQTLs for *SLC6A14* in the pancreas when analyzed in males (black solid line) and in females (dashed blue line) separately.

**S7 Fig.** **Meconium ileus and lung associations with GTEx eQTL profiles for *AGTR2* shows lung association co-localizes with lung-specific *cis*-eQTLs for *AGTR2*.** Overlay of p-values (on the –log_10_ scale) from the meconium ileus GWAS (red/yellow palette of colored dots; this study), lung function GWAS (green/blue palette of colored dots; [9]) and GTEx (v7, [4]) eQTLs association for *AGTR2* expression (colored lines, derived the same as for Fig 2) for the different tissues of interest. AGTR2 is not expressed in the CF nasal epithelia.

**S8 Fig.** **Type 1 Error evaluation of the Simple Sum colocalization analytical method based on the LD pattern at the *SLC6A14* locus and when the eQTL evidence is measured as -log_10_ transform of eQTL p-value.** Simulation method is outlined in S1 Appendix, and the null case considered is Case 1 described in S6 Table where there is no signal for either GWAS or eQTL. In total, 10^4^ replications were simulated to obtain (A) QQ-plot of the SS colocalization p-value on the original scale, (B) QQ-plot of the SS colocalization p-value on the –log_10_ scale, and (C) the histogram of the SS colocalization p-value that is expected to follow a Unif(0,1) distribution under the null. The empirical Type 1 error is 0.0501 at the 0.05 nominal level, and 0.0053 at the 0.005 level.

**S9 Fig.** **Type 1 Error evaluation of the Simple Sum colocalization analytical method based on the LD pattern at the SLC6A14 locus and when the eQTL evidence is dichotomized using the eQTL p<0.05 threshold.** Simulation method is outlined in S1 Appendix, and the null case considered is Case 1 described in S6 Table where there is no signal for either GWAS or eQTL. In total, 10^4^ replications were simulated to obtain (A) QQ-plot of the SS colocalization p-value on the original scale, (B) QQ-plot of the SS colocalization p-value on the –log_10_ scale, and (C) the histogram of the SS colocalization p-value that is expected to follow a Unif(0,1) distribution under the null. The empirical Type 1 Error is 0.048 at the 0.05 nominal level, and 0.0049 at the 0.005 level.

**S10 Fig.** **Type 1 Error evaluation of the Simple Sum colocalization analytical method based on the LD pattern at the *SLC26A9* locus and when the eQTL evidence is measured as –log_10_ transform of eQTL p values.** Simulation method is outlined in S1 Appendix, and the null case considered is Case 1 described in S6 Table where there is no signal for either GWAS or eQTL. In total, 10^4^ replications were simulated to obtain (A) QQ-plot of the SS colocalization p-value on the original scale, (B) QQ-plot of the SS colocalization p-value on the –log_10_ scale, and (C) the histogram of the SS colocalization p-value that is expected to follow a Unif(0,1) distribution under the null. The empirical Type 1 error is 0.052 at the 0.05 nominal level, and 0.0064 at the 0.005 level.

**S11 Fig.** **Type 1 Error evaluation of the Simple Sum colocalization analytical method based on the LD pattern at the *SLC26A9* locus and when the eQTL evidence is dichotomized using the eQTL p<0.05 threshold.** Simulation method is outlined in S1 Appendix, and the null case considered is Case 1 described in S6 Table where there is no signal for either GWAS or eQTL. In total, 10^4^ replications were simulated to obtain (A) QQ-plot of the SS colocalization p-value on the original scale, (B) QQ-plot of the SS colocalization p-value on the –log_10_ scale, and (C) the histogram of the SS colocalization p-value that is expected to follow a Unif(0,1) distribution under the null. The empirical Type 1 error is 0.0509 at the 0.05 nominal level, and 0.0051 at the 0.005 level.

**S12 Fig.** **Type 1 Error evaluation of the Simple Sum colocalization analytic method based on the LD pattern at the *ATP12A* locus and when the eQTL evidence is measured as -log_10_ transform of eQTL p values.** Simulation method is outlined in S1 Appendix, and the null case considered is Case 1 described in S6 Table where there is no signal for either GWAS or eQTL. In total, 10^4^ replications were simulated to obtain (A) QQ-plot of the SS colocalization p-value on the original scale, (B) QQ-plot of the SS colocalization p-value on the –log_10_ scale, and (C) the histogram of the SS colocalization p-value that is expected to follow a Unif(0,1) distribution under the null. The empirical Type 1 error is 0.0487 at the 0.05 nominal level, and 0.0034 at the 0.005 level.

**S13 Fig.** **Type 1 Error evaluation of the Simple Sum colocalization analytical method based on the LD pattern at the *ATP12A* locus and when the eQTL evidence is dichotomized using the eQTL p<0.05 threshold.** Simulation method is outlined in S1 Appendix, and the null case considered is Case 1 described in S6 Table where there is no signal for either GWAS or eQTL. In total, 10^4^ replications were simulated to obtain (A) QQ-plot of the SS colocalization p-value on the original scale, (B) QQ-plot of the SS colocalization p-value on the –log_10_ scale, and (C) the histogram of the SS colocalization p-value that is expected to follow a Unif(0,1) distribution under the null. The empirical Type 1 error is 0.0513 at the 0.05 nominal level, and 0.0046 at the 0.005 level.

**S14 Fig.** **Heatmaps of the Simple Sum colocalization test for a 1Mbp region encompassing the peak meconium ileus-associated variants.** The *SS* colocalization test evaluates if the eQTLs for a given gene and in a given tissue colocalize with meconium ileus-associated variants in the regions of (A) chromosome X, (B) chromosome 1, and (C) chromosome 13. In each panel, each row shows the *SS* colocalization evidence for the specified tissue across all genes within 1Mbp of the peak GWAS variant. *SS* colocalization evidence for each gene is calculated for SNPs within 0.1Mbp of the peak GWAS variants; the genes on the x-axis are ordered by their chromosomal positions. Each column shows the *SS* colocalization evidence for the specified gene across each tissue tested. The color intensity corresponds to the *SS* colocalization evidence as measured by –log_10_(*SS* p-value), with red representing –log_10_(p)=6 and white representing –log_10_(p)=0. Grey indicates either insufficient expression levels attained for the gene in the tissue under study, or that there were no significant eQTLs for the gene in that tissue. The eQTL analyses used for all gene/tissue pairs are those conducted by GTEx version 7 release, except the boxes indicated on the margins. eQTL analysis for the boxes on the margins were calculated in version 6 but were not calculated in GTEx version 7 due to a more stringent expression threshold criteria set in GTEx v7 versus v6 (see Material and Methods for specifics); these analyses were conducted using the publicly available expression matrix (phe000020.v1) and genotypes from WGS (phg000830.v1) following GTEx’s protocol for eQTL analysis.

**S15 Fig.**  **Type 1 Error evaluation of the Simple Sum Contrasting colocalization analytical method based on the LD pattern at the *SLC6A14* locus and when the eQTL evidence is measured as -log_10_ transform of eQTL p values.** Simulation method is outlined in S1 Appendix, and the null case considered is Case 1 described in S6 Table where there is no signal for either GWAS or eQTL. In total, 10^4^ replications were simulated to obtain (A) QQ-plot of the simple sum contrasting (*SSC*) colocalization p-value on the original scale, (B) QQ-plot of the *SSC* colocalization p-value on the –log_10_ scale, and (C) the histogram of the *SSC* colocalization p-value that is expected to follow a Unif(0,1) distribution under the null hypothesis. The empirical Type 1 Error is 0.0536 at the 0.05 nominal level, and 0.0048 at the 0.005 level.

**S16 Fig.**  **Type 1 Error evaluation of the Simple Sum Contrasting colocalization analytical method based on the LD pattern at the *SLC6A14* locus and when the eQTL evidence is dichotomized using the eQTL p<0.05 threshold.** Simulation method is outlined in S1 Appendix, and the null case considered is Case 1 described in S6 Table where there is no signal for either GWAS or eQTL. In total, 10^4^ replications were simulated to obtain (A) QQ-plot of the simple sum contrasting (*SSC*) colocalization p-value on the original scale, (B) QQ-plot of the *SSC* colocalization p-value on the –log_10_ scale, and (C) the histogram of the *SSC* colocalization p-value that is expected to follow a Unif(0,1) distribution under the null hypothesis. The empirical Type 1 error is 0.0454 at the 0.05 nominal level, and 0.0043 at the 0.005 level.

**S17 Fig.**  **Type 1 Error evaluation of the Simple Sum Contrasting colocalization analytical method based on the LD pattern at the *SLC26A9* locus and when the eQTL evidence is measured as - log_10_ transform of eQTL p values.**  Simulation method is outlined in S1 Appendix, and the null case considered is Case 1 described in S6 Table where there is no signal for either GWAS or eQTL. In total, 10^4^ replications were simulated to obtain (A) QQ-plot of the simple sum contrasting (*SSC*) colocalization p-value on the original scale, (B) QQ-plot of the *SSC* colocalization p-value on the –log_10_ scale, and (C) the histogram of the *SSC* colocalization p-value that is expected to follow a Unif(0,1) distribution under the null hypothesis. The empirical Type 1 error is 0.0457 at the 0.05 nominal level, and 0.0039 at the 0.005 level.

**S18 Fig. Type 1 Error evaluation of the Simple Sum Contrasting colocalization analytical method based on the LD pattern at the *SLC26A9* locus and when the eQTL evidence is dichotomized using the eQTL p<0.05 threshold.** Simulation method is outlined in S1 Appendix, and the null case considered is Case 1 described in S6 Table where there is no signal for either GWAS or eQTL. In total, 10^4^ replications were simulated to obtain (A) QQ-plot of the simple sum contrasting (*SSC*) colocalization p-value on the original scale, (B) QQ-plot of the *SSC* colocalization p-value on the –log_10_ scale, and (C) the histogram of the *SSC* colocalization p-value that is expected to follow a Unif(0,1) distribution under the null hypothesis. The empirical Type 1 error is 0.0512 at the 0.05 nominal level, and 0.0052 at the 0.005 level.

**S19 Fig.**  **Type 1 Error evaluation of the Simple Sum Contrasting colocalization analytical method based on the LD pattern at the *ATP12A* locus and when the eQTL evidence is measured as - log_10_ transform of eQTL p values.** Simulation method is outlined in S1 Appendix, and the null case considered is Case 1 described in S6 Table where there is no signal for either GWAS or eQTL. In total, 10^4^ replications were simulated to obtain (A) QQ-plot of the simple sum contrasting (*SSC*) colocalization p-value on the original scale, (B) QQ-plot of the *SSC* colocalization p-value on the –log_10_ scale, and (C) the histogram of the *SSC* colocalization p-value that is expected to follow a Unif(0,1) distribution under the null hypothesis. The empirical Type 1 error is 0.052 at the 0.05 nominal level, and 0.0048 at the 0.005 level.

**S20 Fig.**  **Type 1 Error evaluation of the Simple Sum Contrasting colocalization analytical method based on the LD pattern at the *ATP12A* locus and when the eQTL evidence is dichotomized using the eQTL p<0.05 threshold.** Simulation method is outlined in S1 Appendix, and the null case considered is Case 1 described in S6 Table where there is no signal for either GWAS or eQTL. In total, 10^4^ replications were simulated to obtain (A) QQ-plot of the simple sum contrasting (*SSC*) colocalization p-value on the original scale, (B) QQ-plot of the *SSC* colocalization p-value on the –log_10_ scale, and (C) the histogram of the *SSC* colocalization p-value that is expected to follow a Unif(0,1) distribution under the null hypothesis. The empirical Type 1 error is 0.0503 at the 0.05 nominal level, and 0.0051 at the 0.005 level.

# Supplemental Tables

**S1 Table. Total sample after quality control.** The total number of individuals used for the meconium ileus association analysis after quality control of both genotypes and phenotypes, stratified by consortium site and Illumina genotyping platform.

**S2 Table. SNP Quality Control Steps**. The number of SNPs before quality control (QC) and the number of SNPs excluded by each stated QC criterion, stratified by consortium sites and Illumina genotyping platform.

**S3 Table. Comparison of meconium ileus association results between this study** **and the previous consortium GWAS.** SNPs significant in either study (this study with n=6770 and previous consortium GWAS published in Sun et al [10] ) and of particular functional relevance are provided in this table.

**S4 Table. Sex-specific association analysis of variants in *SLC6A14*.** The three variants here include the top two ranked SNPs, rs3788766 and rs5905177 as in S3 Table, and putative functional variant rs12710568**.**

**S5 Table:** **Parameter values for the simulation studies.**  Values of standardized true effect size of the associated SNP for GWAS or an eQTL used in the various simulation settings, with the corresponding GWAS or eQTL power of individually detecting the SNP at the 10^-8^ significance level, and the expected -log10(p-value) of the GWAS association or the eQTL analysis if the observed signal strength is the true effect size. See S1 Appendix for other simulation details.

**S6 Table: Overview and illustration of the cases under the composite null hypothesis that there is no colocalization.** Plots (a)-(d) provide the general visualization of GWAS (red line) and eQTL (blue) patterns (on the -log10 p scale) in a region of interest (e.g. the *SLC6A14* locus). The value of $\lambda_{Z_{c}}$represents the standardized true effect size of a GWAS associated variant, and $\lambda_{T_{c}}$represents the true standardized effect size of an eQTL variant. The corresponding power of detecting the GWAS SNP or finding the eQTL are provided in S5 Table. For illustration purposes but without loss generality, if there was a GWAS association, $\lambda_{Z_{c}}$was set to be 5.73 such that 0.5 power could be achieved to detect the signal. If there was an eQTL from the gene-expression study, $\lambda_{T_{c}}$was set to be 7.01 such that 0.9 power could be achieved to detect the signal. See S1 Appendix for other simulation details.

**S7 Table: Overview with illustration of the cases under different types of alternatives when there is colocalization at at least one variant in the region.** Plots (a)-(f) provide the general visualization of GWAS (red line) and eQTL (blue) patterns (on the -log10 p scale) in a region of interest (e.g. the *SLC6A14* locus). The parameter values are varied in the power study, thus they are provided in the corresponding referred tables.

**S8 Table: Type 1 error evaluation of the proposed Simple Sum colocalization analytical method, and the false positive rate of COLOC** **and eCAVIAR under the different null cases.** The null cases considered are detailed in S6 Table. The LD pattern at the simulated region follows that at the *SLC6A14* locus. For the SS method, the nominal type 1 error was set at alpha=0.05 or alpha= 0.005. The eQTL evidence was measured continuously as -log10 (eQTL p-value), or dichotomized using the eQTL p<0.05 or <0.005 threshold. For COLOC and eCAVIAR, the false positive rates were calculated by applying the 0.5, 0.75 or 0.9 threshold (as in [1]) to the colocalization posterior probability. In total, 10^4^ replications were simulated to obtain each cell of the table. See S1 Appendix for other simulation details.

**S9 Table.** **Type 1 error evaluation of the proposed Simple Sum colocalization analytical method under the null Case 3.** All the null cases considered are detailed in S6 Table. Case 3 is the situation when there is a GWAS associated SNP but there is no eQTL (plot (c) in S6 Table). In that case, colocalization results should be interpreted with caution if the observed eQTL signal is weak with examples demonstrated below (i.e. the null hypothesis is not rejected if the maximum of -log10 (eQTL p-value) is below a threshold (column 1)). Among the remaining replicates, the null is rejected if the Simple Sum colocalization p-value is smaller than the nominal type 1 error level (alpha=0.05 or alpha=0.005). The LD pattern at the simulated region follows that at the *SLC6A14* locus. In total, 10^4^ replications were simulated to obtain each cell of the table. See S1 Appendix for other simulation details.

**S10 Table. Results of Simple Sum colocalization and contrasting colocalization analyses for the three loci genome-wide significantly associated with meconium ileus.** The eQTL evidence was dichotomized by using thresholds of eQTL p<0.05, <0.005 or <0.0005 instead of based on the -log10(p-value) as in Table 3. Analytical and permutation-based (# of replicates=10^5^) Simple Sum *(SS)* colocalization p-values evaluate if the eQTLs for a given gene and tissue colocalize with meconium ileus-associated variants. All colocalization p-values were one-sided because only positive association implies eQTL-association colocalization (i.e. eQTL peaks coincide with association peaks). Simple Sum Contrasting (*SSC*) colocalization p-value evaluates if the eQTLs in the pancreas colocalize with meconium ileus-associated variants more than eQTLs in another tissue; NAs are listed for the pancreas since we do not contrast pancreas with itself. Other NAs are used when there are no SNPs with eQTLs p less than the thresholds considered (0.05, 0.005 or 0.0005) for that gene and tissue.

**S11 Table. Power evaluation of the proposed Simple Sum colocalization analytical method, and the true positive rate of COLOC and eCAVIAR, under the alternative that ONE GWAS and ONE eQTL locus colocolizes (Alter1 in S7 Table)**. The LD pattern at the simulated region follows that at the *SLC6A14* locus. For the SS method, the nominal type 1 error was set at alpha=0.05 or alpha= 0.005. The eQTL evidence was measured continuously as -log10 (eQTL p-value), or dichotomized using the eQTL p<0.05 or <0.005 threshold. For COLOC and eCAVIAR, the false positive rates were calculated by applying the 0.5, 0.75 or 0.9 threshold to the colocalization posterior probability. The value of $\lambda_{Z_{c}}$represents the standardized true effect size of the GWAS associated variant, and $\lambda_{T_{c}}$represents the standardized true effect size of the eQTL variant, as detailed in S5 Table. Here, $\lambda_{Z_{c}}$is set to be 5.73 such that 0.5 power is achieved to detect the GWAS association at significance level of 10^-8^, while $\lambda_{T_{c}}$is set to be 3.4, 4.09, 4.45, 5.21 or 5.73 for each row of the table such that 0.01, 0.05, 0.1, 0.3, or 0.5 power is achieved to detect the eQTL association at significance level 10^-8^. In total, 10^4^ replications were simulated to obtain each cell of the table. See S1 Appendix for other simulation details.

**S12 Table. Power evaluation of the proposed Simple Sum colocalization analytical method, and the true positive rate of COLOC** **and eCAVIAR, under the alternative that the eQTL peak overlapped with the higher GWAS peak (Alter2 in S7 Table).** The LD pattern at the simulated region follows that at the *SLC6A14* locus. For the SS method, the nominal type 1 error was set at alpha=0.05 or alpha= 0.005. The eQTL evidence was measured continuously as -log10 (eQTL p-value) or dichotomized using the eQTL p<0.05 or <0.005 threshold. For COLOC and eCAVIAR, the false positive rates were calculated by applying the 0.5, 0.75 or 0.9 threshold to the colocalization posterior probability. The values of $\lambda_{Z_{c1}}$and $\lambda_{Z_{c2}}$represent the standardized true effect sizes of two GWAS associated variants, while $\lambda_{T_{c1}}$and $\lambda_{T_{c2}}$represent the standardized true effect sizes of two eQTL variants. Here, $\lambda_{Z_{c1}}$is set to be 6.57 and $\lambda_{Z_{c2}}$is set to be 5.73 such that, respectively, 0.8 and 0.5 power are achieved to detect the two GWAS signals at significance level 10^-8^. $\lambda_{T_{c1}}$is set to be 3.4, 4.09, 4.45, 5.21 or 5.73 for each row of the table such that 0.01, 0.05, 0.1, 0.3, or 0.5 power is achieved to detect the eQTL association at significance level of 10^-8^ and $\lambda_{T_{c2}}$is set to be 0. In total, 10^4^ replications were simulated to obtain each cell of the table. See S1 Appendix for other simulation details.

**S13 Table. Power evaluation of the proposed Simple Sum colocalization analytical method, and the true positive rate of COLOC** **and eCAVIAR, under the alternative that the eQTL peak overlapped with the lower GWAS peak (Alter3 in S7 Table).** The LD pattern at the simulated region follows that at the *SLC6A14* locus. For the SS method, the nominal type 1 error was set at alpha=0.05 or alpha= 0.005. The eQTL evidence was measured continuously as -log10 (eQTL p-value) or dichotomized using the eQTL p<0.05 or <0.005 threshold. For COLOC and eCAVIAR, the false positive rates were calculated by applying the 0.5, 0.75 or 0.9 threshold to the colocalization posterior probability. The values of $\lambda_{Z_{c1}}$and $\lambda_{Z_{c2}}$represent the standardized true effect sizes of two GWAS associated variants, while $\lambda_{T_{c1}}$and $\lambda_{T_{c2}}$represent the standardized true effect sizes of two eQTL variants. Here, $\lambda_{Z_{c1}}$is set to be 6.57 and $\lambda_{Z_{c2}}$is set to be 5.73 such that 0.8 and 0.5 power are achieved to detect GWAS signals at significance level of 10^-8^. $\lambda_{T_{c1}}$is set to be 0, and $\lambda_{T_{c2}}$is set to be 3.4, 4.09, 4.45, 5.21 or 5.73 for each row of the table such that 0.01, 0.05, 0.1, 0.3, or 0.5 power is achieved to detect the eQTL association at significance level 10^-8^. In total, 10^4^ replications were simulated to obtain each cell of the table. See S1 Appendix for other simulation details.

**S14 Table. Power evaluation of the proposed Simple Sum colocalization analytical method, and the true positive rate of COLOC and eCAVIAR, under the alternative that the non-overlapped eQTL peak is lower than the GWAS peak (Alter4 in S7 Table).** The LD pattern at the simulated region follows that at the *SLC6A14* locus. For the SS method, the nominal type 1 error was set at alpha=0.05 or alpha= 0.005. The eQTL evidence was measured continuously as -log10 (eQTL p-value) or dichotomized using the eQTL p<0.05 or <0.005 threshold. For COLOC and eCAVIAR, the false positive rates were calculated by applying the 0.5, 0.75 or 0.9 threshold to the colocalization posterior probability. The values of $\lambda_{Z_{c1}}$and $\lambda_{Z_{c2}}$represent the standardized true effect sizes of two GWAS associated variants, while $\lambda_{T_{c1}}$and $\lambda_{T_{c2}}$represent standardized true effect sizes of two eQTL variants. Here, $\lambda_{Z_{c1}}$is set to be 6.57 such that 0.8 power is achieved to detect that GWAS signal at significance level 10^-8^ and $\lambda_{Z_{c2}}$is set to be 0. ${\lambda_{T}}_{c_{1}}$is set to be 3.4, 4.09, 4.45, 5.21 or 5.73 for each row of the table such that 0.01, 0.05,0.1,0.3, or 0.5 power are achieved to detect the eQTL association at significance level of 10^-8^, and $\lambda_{T_{c2}}$is set to be 5.73 such that 0.5 power is achieved to detect that eQTL signal at significance level of 10^-8^. In total, 10^4^ replications were simulated to obtain each cell of the table. See S1 Appendix for other simulation details.

**S15 Table. Power evaluation of the proposed Simple Sum colocalization analytical method, and the true positive rate of COLOC and eCAVIAR, under the alternative that the non-overlapped eQTL peak is higher than the GWAS peak (Alter5 in S7 Table).** The LD pattern at the simulated region follows that at the *SLC6A14* locus. For the SS method, the nominal type 1 error was set at alpha=0.05 or alpha= 0.005. The eQTL evidence was measured continuously as -log10 (eQTL p-value) or dichotomized using the eQTL p<0.05 or <0.005 threshold. For COLOC and eCAVIAR, the false positive rates were calculated by applying the 0.5, 0.75 or 0.9 threshold to the colocalization posterior probability. The values of $\lambda_{Z_{c1}}$and $\lambda_{Z_{c2}}$represent the standardized true effect sizes of two GWAS associated variants, while $\lambda_{T_{c1}}$and $\lambda_{T_{c2}}$represent the standardized true effect sizes of eQTL variants. Here, $\lambda_{Z_{c1}}$is set to be 6.57 such that 0.8 power is achieved to detect that GWAS signal at significance level of 10^-8^ and $\lambda_{Z_{c2}}$is set to be 0. ${\lambda_{T}}_{c_{1}}$is set to be 3.4, 4.09, 4.45, 5.21 or 5.73 for each row of the table such that 0.01, 0.05,0.1,0.3, or 0.5 power are achieved to detect the eQTL association at significance level of 10^-8^ and $\lambda_{T_{c2}}$is set to be 7.01 such that 0.9 power is achieved to detect that eQTL signal at significance level of 10^-8^. In total, 10^4^ replications were simulated to obtain each cell of the table. See S1 Appendix for other simulation details.

**S16 Table. Power evaluation of the proposed Simple Sum colocalization analytical method, and the true positive rate of COLOC and eCAVIAR, under the alternative that there are two overlapping GWAS and eQTL loci** **(Alter6 in S7 Table).** The LD pattern at the simulated region follows that at the *SLC6A14* locus. For the SS method, the nominal type 1 error was set at alpha=0.05 or alpha= 0.005. The eQTL evidence was measured continuously as -log10 (eQTL p-value) or dichotomized using the eQTL p<0.05 or <0.005 threshold. For COLOC and eCAVIAR, the false positive rates were calculated by applying the 0.5, 0.75 or 0.9 threshold to the colocalization posterior probability. The values of $\lambda_{Z_{c1}}$and $\lambda_{Z_{c2}}$represent the standardized true effect sizes of two GWAS associated variants, while $\lambda_{T_{c1}}$and $\lambda_{T_{c2}}$represent the standardized true effect sizes of eQTL variants. Here, $\lambda_{Z_{c1}}$is set to be 6.57 and $\lambda_{Z_{c2}}$is set to be 5.73 such that, respectively, 0.8 and 0.5 power are achieved to detect the two GWAS signals at significance level of 10^-8^. ${\lambda_{T}}_{c_{1}}$is set to be 3.4, 4.09, 4.45, 5.21 or 5.73 for each row of the table such that 0.01, 0.05,0.1,0.3, or 0.5 power are achieved to detect the eQTL association at significance level of 10^-8^ and $\lambda_{T_{c2}}$is set to be 7.01 such that 0.9 power is achieved to detect that eQTL signal at significance level of 10^-8^. In total, 10^4^ replications were simulated to obtain each cell of the table. See S1 Appendix for other simulation details.

**S17 Table. Results of Simple Sum colocalization and contrasting colocalization analyses for genes at the Chromosome X locus in the region including 0.1Mbp on either side of the lead SNP in CF human nasal epithelial and lung from GTEx.** The exact region is 115248275-115448275 bp as in human genome reference assembly GRCh37. The eQTL evidence used include the -log_10_ transform of eQTL p value, and dichotomized eQTL p-value indicator by thresholds of eQTL p<0.05 or <0.005 for each specified gene and tissue. We focus on the analysis evaluating whether the eQTLs for *SLC6A14* in lung (or human nasal epithelial; HNE) colocalize with lung-associated variants more than eQTLs for *AGTR2*, *PLS3* and *CXorf61* in lung (or HNE). The contrasting colocalization test for *SLC6A14* is listed as NA since we do not contrast *SLC6A14* with itself; NA in other cells means no eQTL SNP with p<0.05 or p<0.005 for that gene. The column ‘No. of eQTL SNPs’ shows the number of SNPs with eQTL p-values< 0.05 or 0.005 in the 0.1Mbp region; it refers to the number of SNPs for which eQTL p-values were available at the locus when -log_10_(eQTLp) is used (first 4 rows of the table). All p-values are one-sided to ensure colocalization rather than negative correlation.

# References

1. Giambartolomei C, Vukcevic D, Schadt EE, Franke L, Hingorani AD, Wallace C, et al. Bayesian test for colocalisation between pairs of genetic association studies using summary statistics. PLoS Genet. 2014;10(5):e1004383.

2. Hormozdiari F, van de Bunt M, Segre AV, Li X, Joo JW, Bilow M, et al. Colocalization of GWAS and eQTL Signals Detects Target Genes. Am J Hum Genet. 2016;99(6):1245-60.

3. Genomes Project Consortium, Auton A, Brooks LD, Durbin RM, Garrison EP, Kang HM, et al. A global reference for human genetic variation. Nature. 2015;526(7571):68-74.

4. GTEx Consortium. The Genotype-Tissue Expression (GTEx) project. Nature genetics. 2013;45(6):580-5.

5. Wen X, Pique-Regi R, Luca F. Integrating molecular QTL data into genome-wide genetic association analysis: Probabilistic assessment of enrichment and colocalization. PLoS Genet. 2017;13(3):e1006646.

6. He X, Fuller CK, Song Y, Meng Q, Zhang B, Yang X, et al. Sherlock: detecting gene-disease associations by matching patterns of expression QTL and GWAS. Am J Hum Genet. 2013;92(5):667-80.

7. International HapMap C, Altshuler DM, Gibbs RA, Peltonen L, Altshuler DM, Gibbs RA, et al. Integrating common and rare genetic variation in diverse human populations. Nature. 2010;467(7311):52-8.

8. Pruim RJ, Welch RP, Sanna S, Teslovich TM, Chines PS, Gliedt TP, et al. LocusZoom: regional visualization of genome-wide association scan results. Bioinformatics. 2010;26(18):2336-7.

9. Corvol H, Blackman SM, Boëlle PY, Gallins PJ, Pace RG, Stonebraker JR, et al. Genome-wide association meta-analysis identifies five modifier loci of lung disease severity in cystic fibrosis. Nat Commun. 2015;6:8382.

10. Sun L, Rommens JM, Corvol H, Li W, Li X, Chiang TA, et al. Multiple apical plasma membrane constituents are associated with susceptibility to meconium ileus in individuals with cystic fibrosis. Nat Genet. 2012;44(5):562-9.
