# Supplementary figures and images for: Genetic association and transcriptome integration identify contributing genes and tissues at cystic fibrosis modifier loci
Source: PLoS Genet. 2019 Feb 26;15(2):e1008007. doi: 10.1371/journal.pgen.1008007 (PMC6407791; doi:10.1371/journal.pgen.1008007)

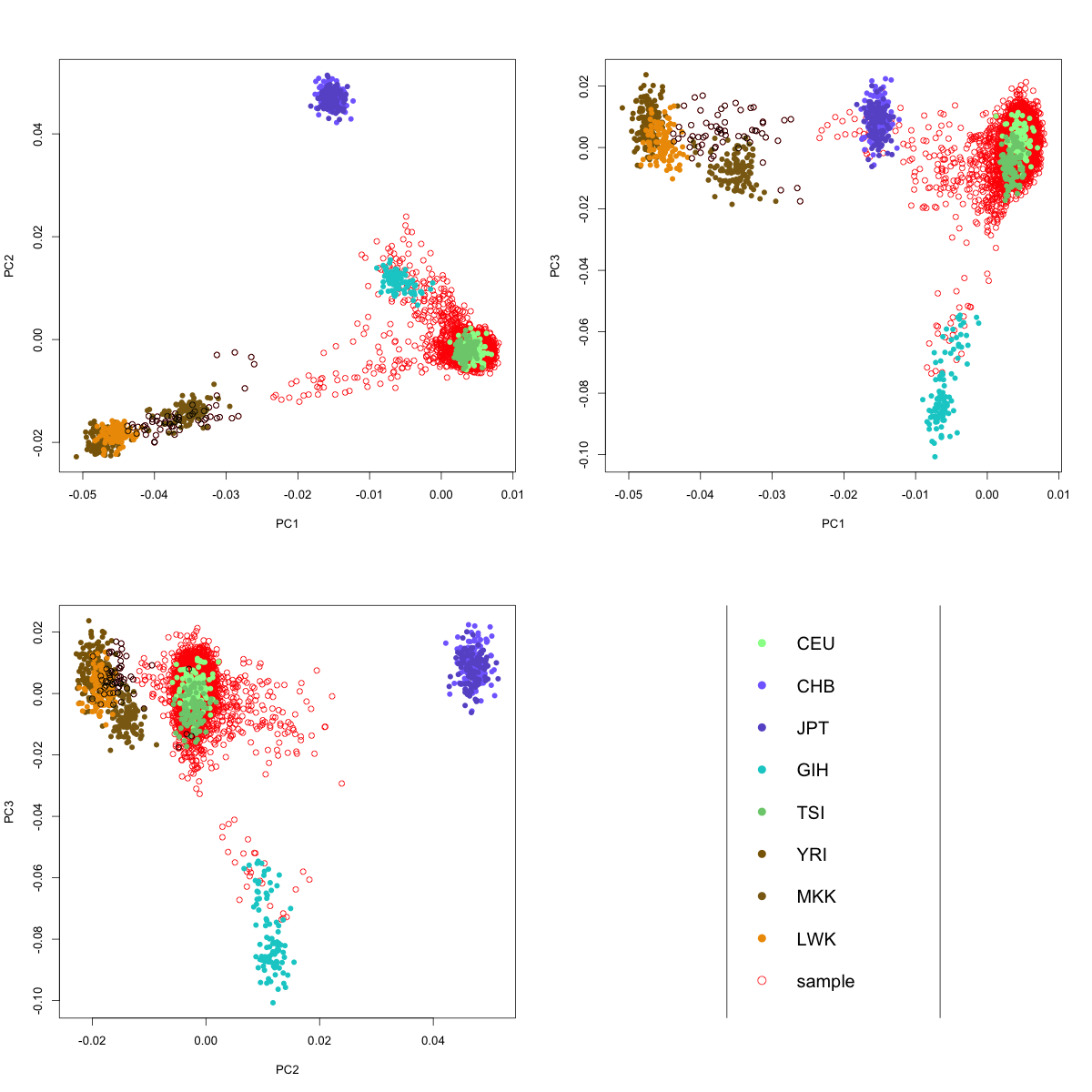

Supplement: S1 Fig — Different solid dots show the clusters of the samples from the International Hapmap consortium [7]. The red circles correspond to our samples with outliers highlighted as black dots; outliers defined as 6 S.D. away from the center of the HapMap3 European (CEU/TSI) cluster. See the detailed description of the Hapmap samples at http://www.sanger.ac.uk/resources/downloads/human/hapmap3.html. (TIF) [file pgen.1008007.s002.tif]

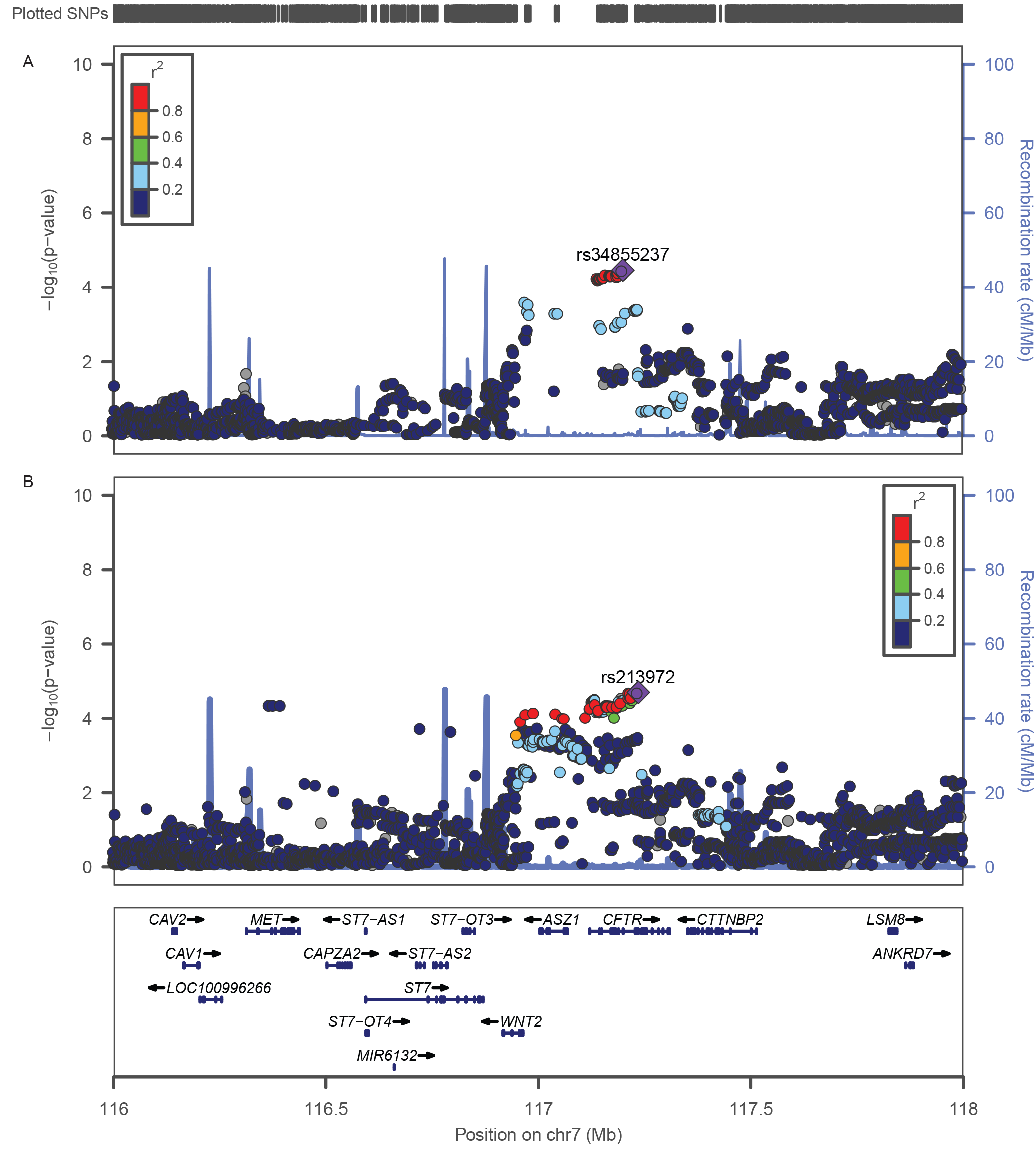

Supplement: S2 Fig — The Locus zoom plot [8] of CFTR association imputed using (A) the 1000 Genome Project Phase 3 [3], and (B) the hybrid reference (augmenting the 1000 genome reference with the whole genome sequencing from 101 patients with CF). Imputed variants with MAF>1% were analyzed. (TIF) [file pgen.1008007.s003.tif]

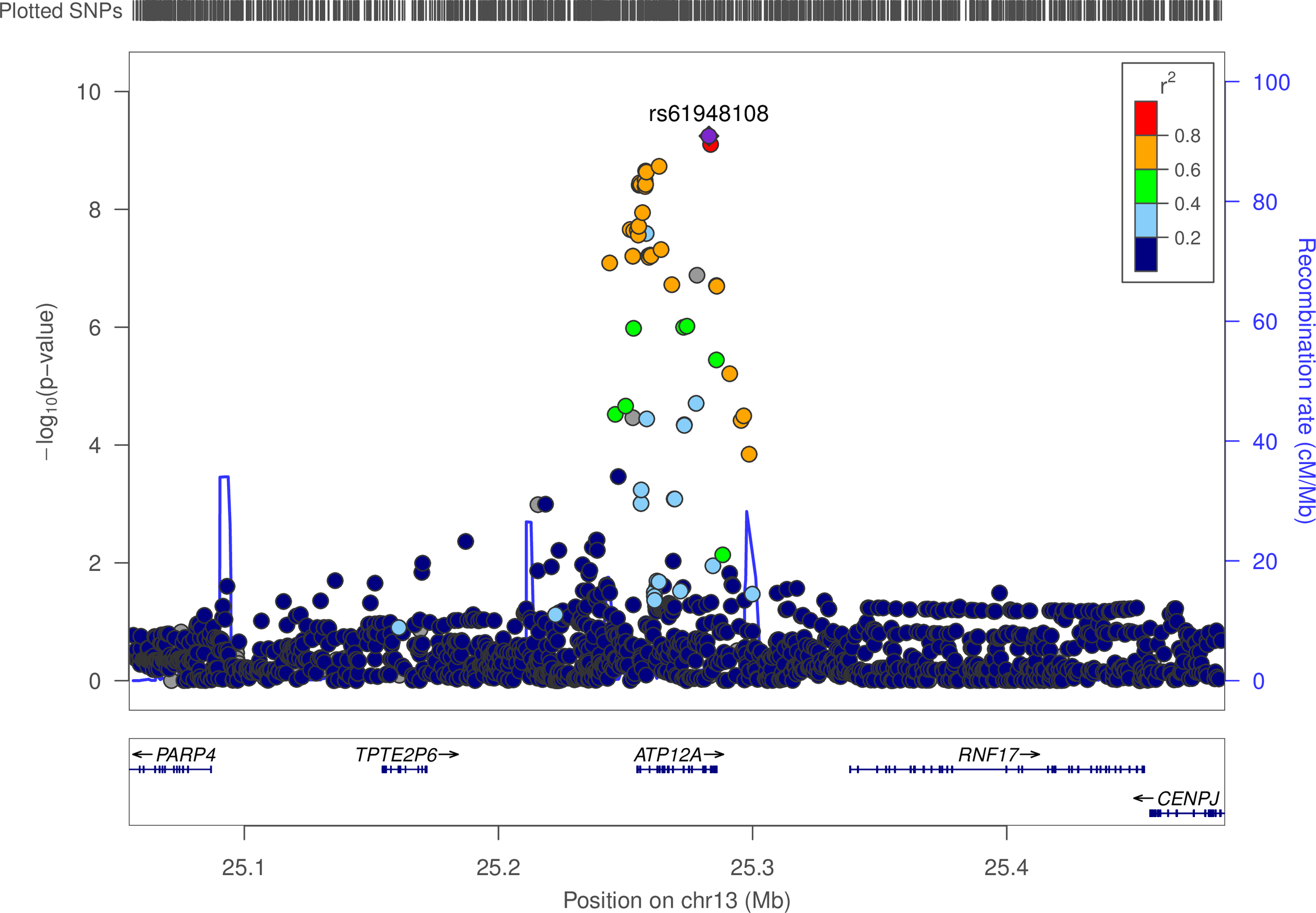

Supplement: S3 Fig — (TIF) [file pgen.1008007.s004.tif]

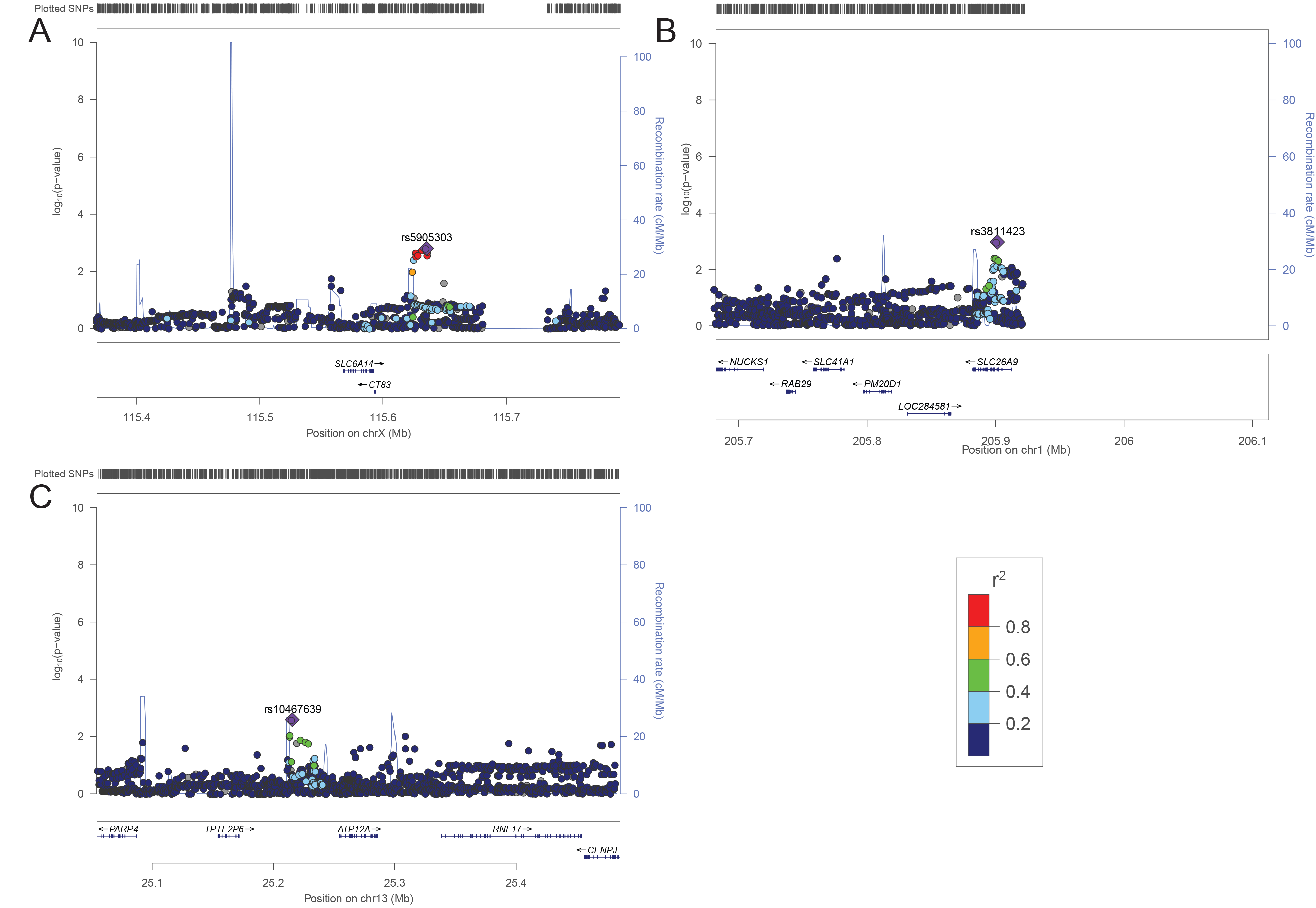

Supplement: S4 Fig — All subfigures are plotted in 200kb regions surrounding (A) SLC6A14, (B) SLC26A9, and (C) ATP12A. The GWAS signals are obliterated after conditioning on the top SNP in each region. The color of each dot represents the amount of LD of the SNP to the purple diamond point, which is the top SNP in each region after the conditional analysis. (TIF) [file pgen.1008007.s005.tif]

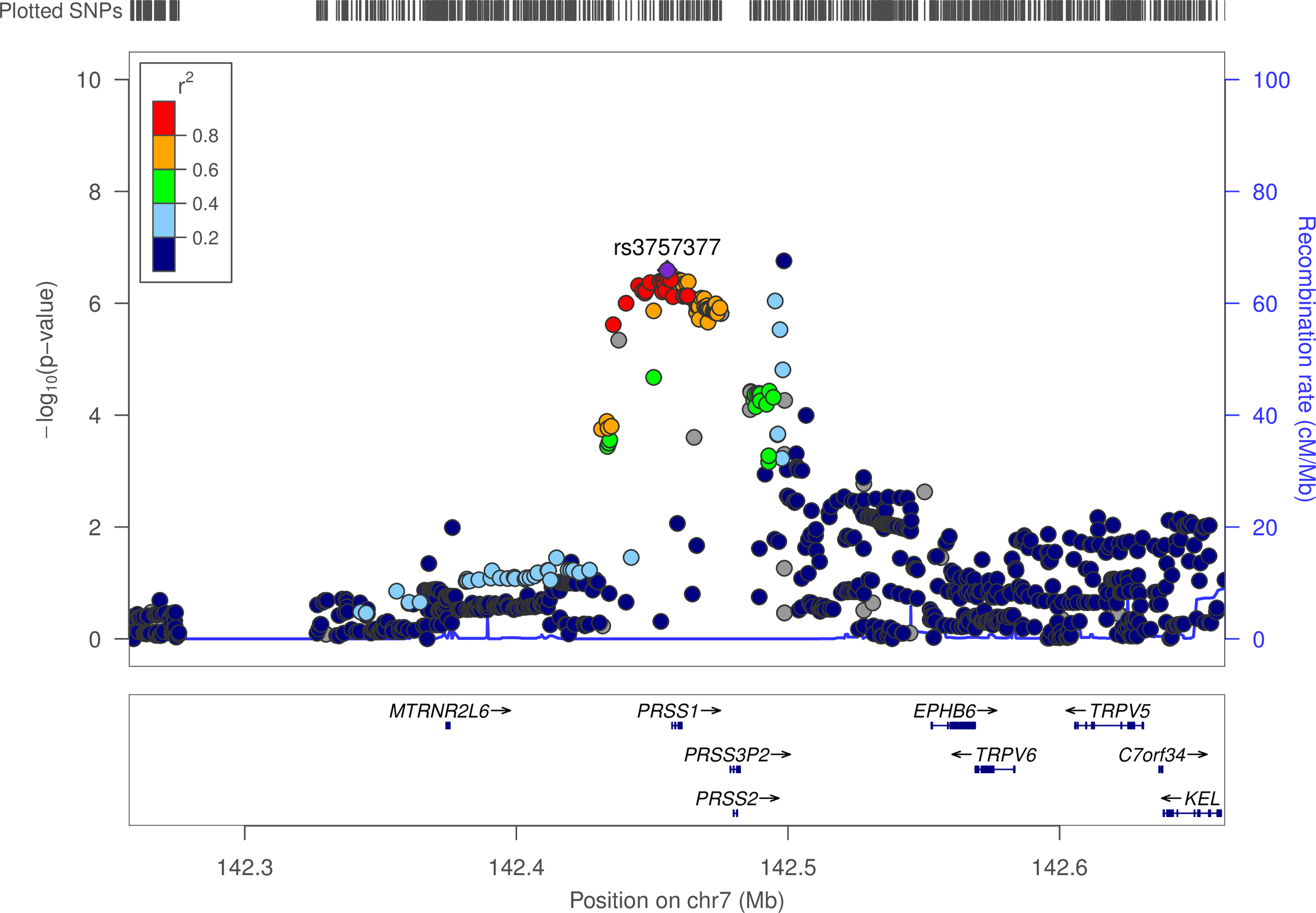

Supplement: S5 Fig — (TIF) [file pgen.1008007.s006.tif]

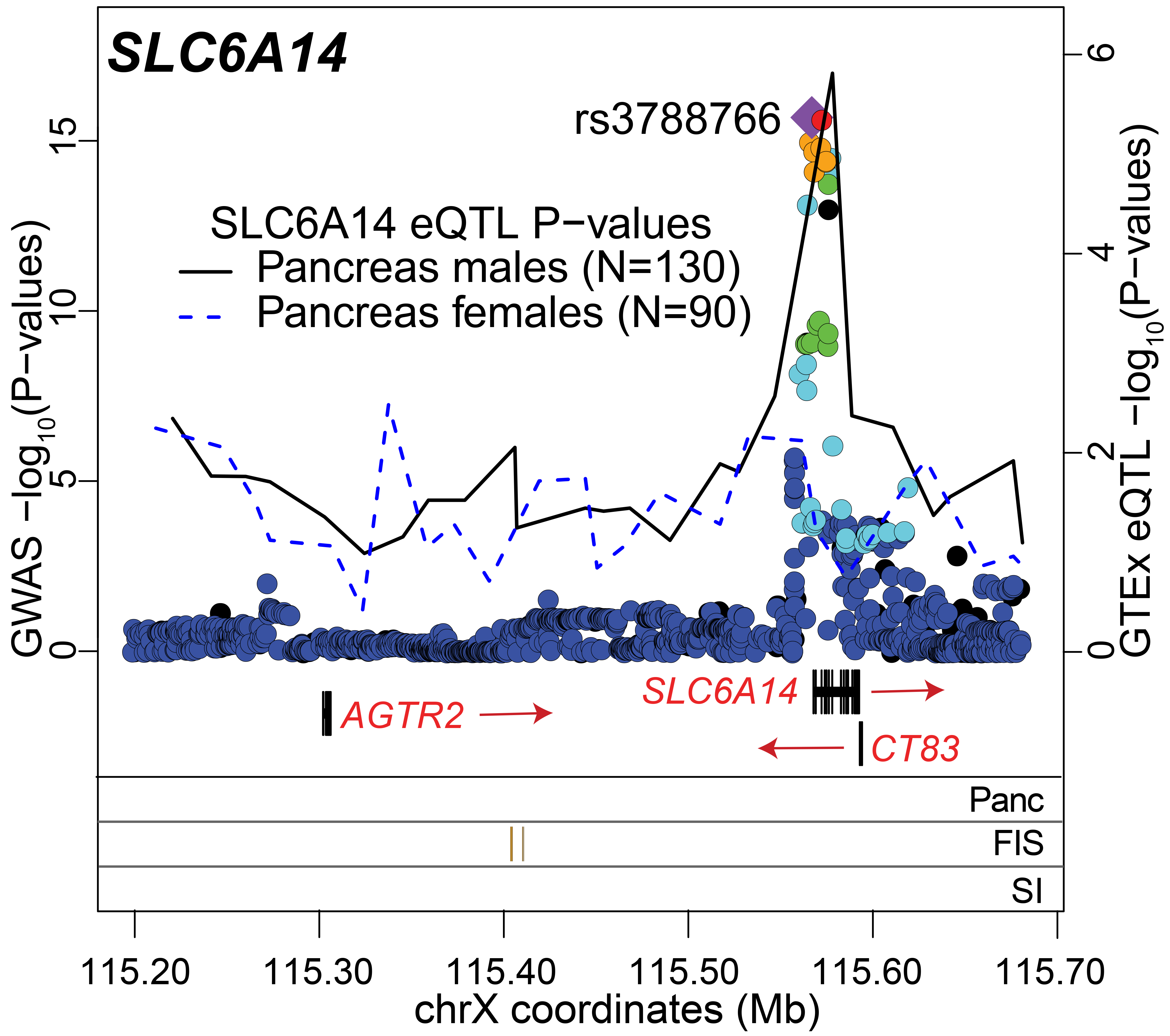

Supplement: S6 Fig — GTEx expression data was analyzed separately by sex for eQTL association with SLC6A14 in the pancreas using the same linear regression model described in Materials and Methods, GTEx Data without the sex covariate. Dots in the figure represent the association with MI, while the lines depict the association pattern of eQTLs for SLC6A14 in the pancreas when analyzed in males (black solid line) and in females (dashed blue line) separately. (TIF) [file pgen.1008007.s007.tif]

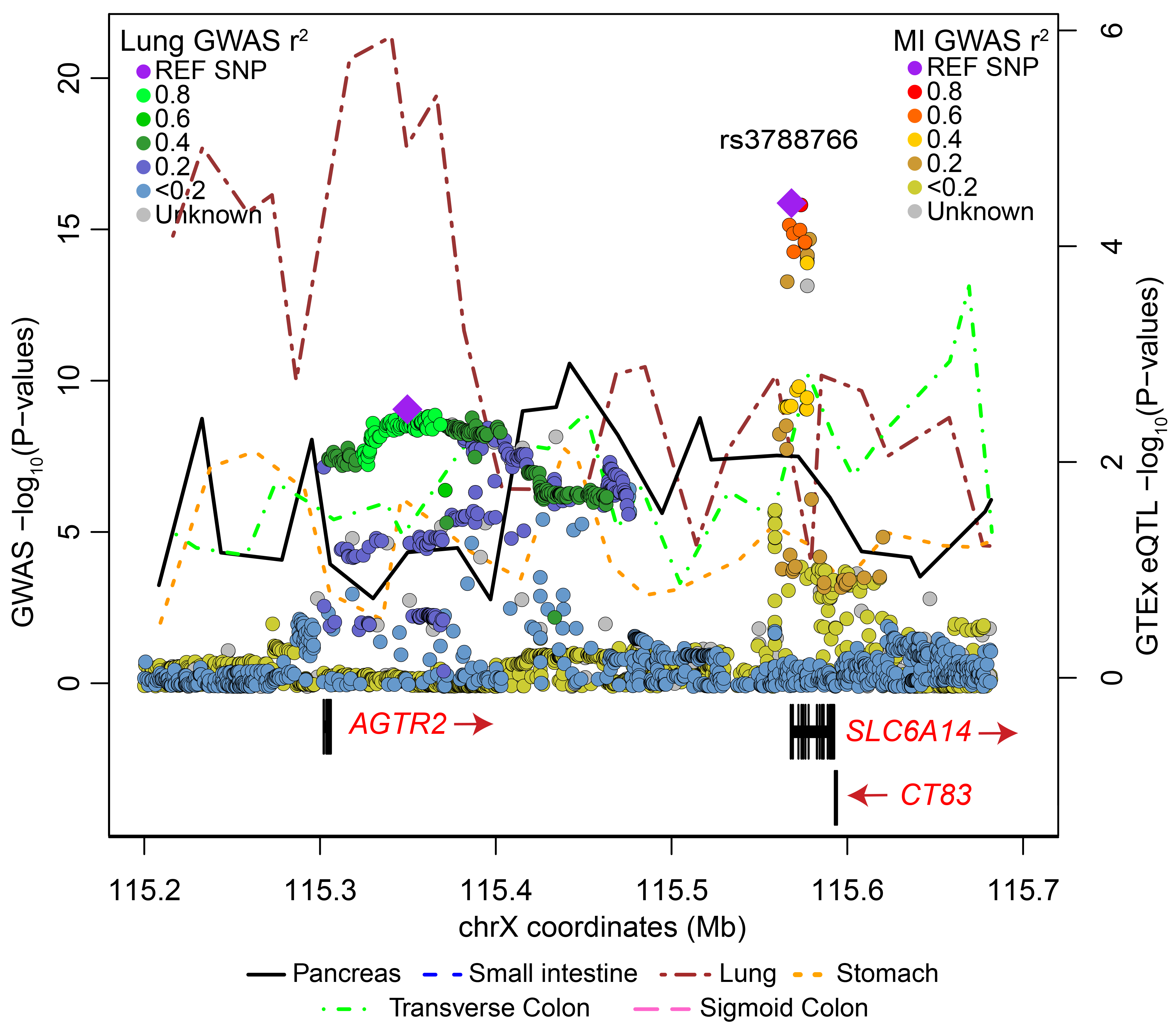

Supplement: S7 Fig — Overlay of p-values (on the −log10 scale) from the meconium ileus GWAS (red/yellow palette of colored dots; this study), lung function GWAS (green/blue palette of colored dots; [9]) and GTEx (v7, [4]) eQTLs association for AGTR2 expression (colored lines, derived the same as for Fig 2) for the different tissues of interest. AGTR2 is not expressed in the CF nasal epithelia. (TIF) [file pgen.1008007.s008.tif]

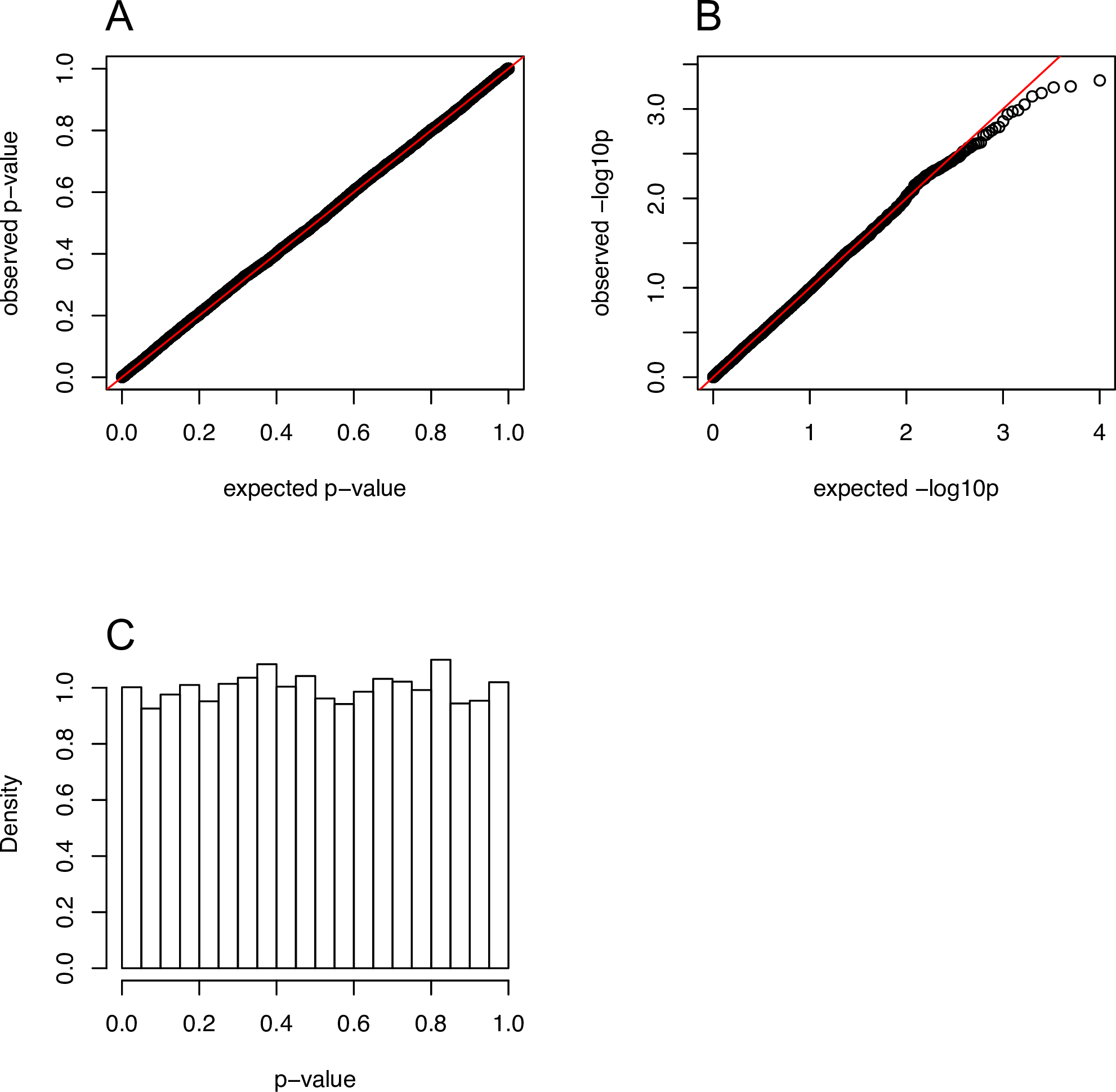

Supplement: S8 Fig — Simulation method is outlined in S1 Appendix, and the null case considered is Case 1 described in S6 Table where there is no signal for either GWAS or eQTL. In total, 104 replications were simulated to obtain (A) QQ-plot of the SS colocalization p-value on the original scale, (B) QQ-plot of the SS colocalization p-value on the −log10 scale, and (C) the histogram of the SS colocalization p-value that is expected to follow a Unif(0,1) distribution under the null. The empirical Type 1 error is 0.0501 at the 0.05 nominal level, and 0.0053 at the 0.005 level. (TIF) [file pgen.1008007.s009.tif]

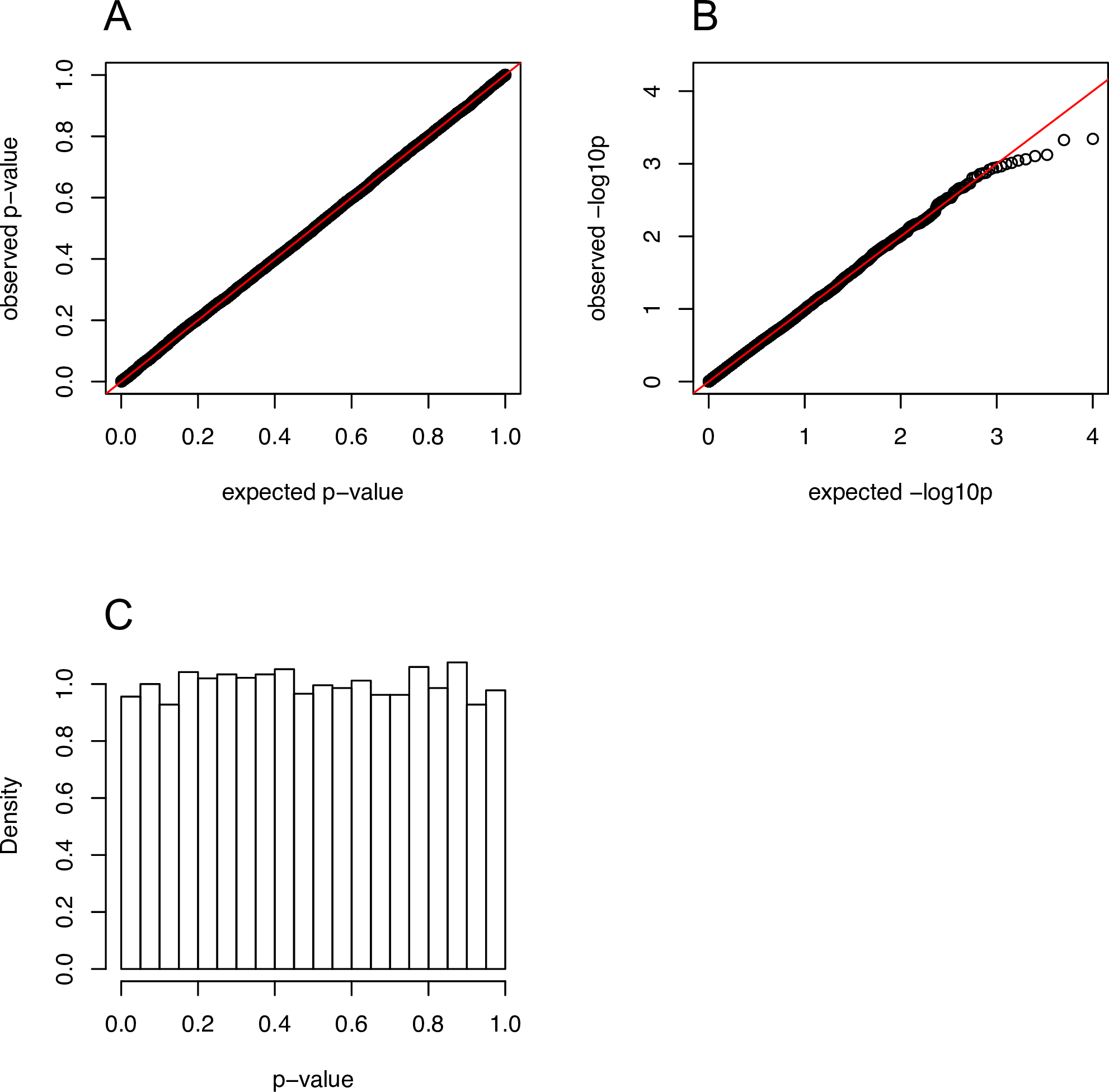

Supplement: S9 Fig — Simulation method is outlined in S1 Appendix, and the null case considered is Case 1 described in S6 Table where there is no signal for either GWAS or eQTL. In total, 104 replications were simulated to obtain (A) QQ-plot of the SS colocalization p-value on the original scale, (B) QQ-plot of the SS colocalization p-value on the −log10 scale, and (C) the histogram of the SS colocalization p-value that is expected to follow a Unif(0,1) distribution under the null. The empirical Type 1 Error is 0.048 at the 0.05 nominal level, and 0.0049 at the 0.005 level. (TIF) [file pgen.1008007.s010.tif]

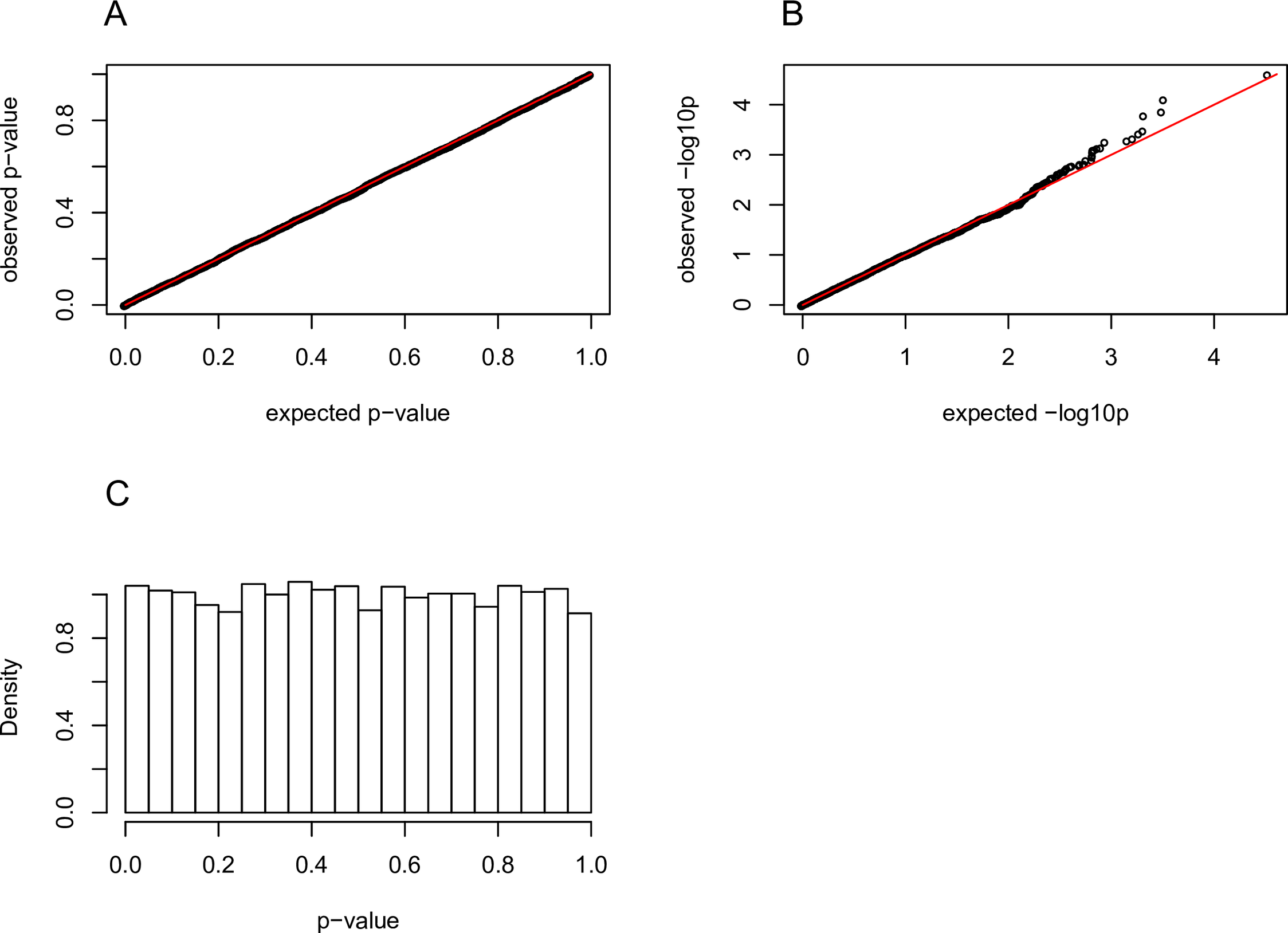

Supplement: S10 Fig — Simulation method is outlined in S1 Appendix, and the null case considered is Case 1 described in S6 Table where there is no signal for either GWAS or eQTL. In total, 104 replications were simulated to obtain (A) QQ-plot of the SS colocalization p-value on the original scale, (B) QQ-plot of the SS colocalization p-value on the −log10 scale, and (C) the histogram of the SS colocalization p-value that is expected to follow a Unif(0,1) distribution under the null. The empirical Type 1 error is 0.052 at the 0.05 nominal level, and 0.0064 at the 0.005 level. (TIF) [file pgen.1008007.s011.tif]

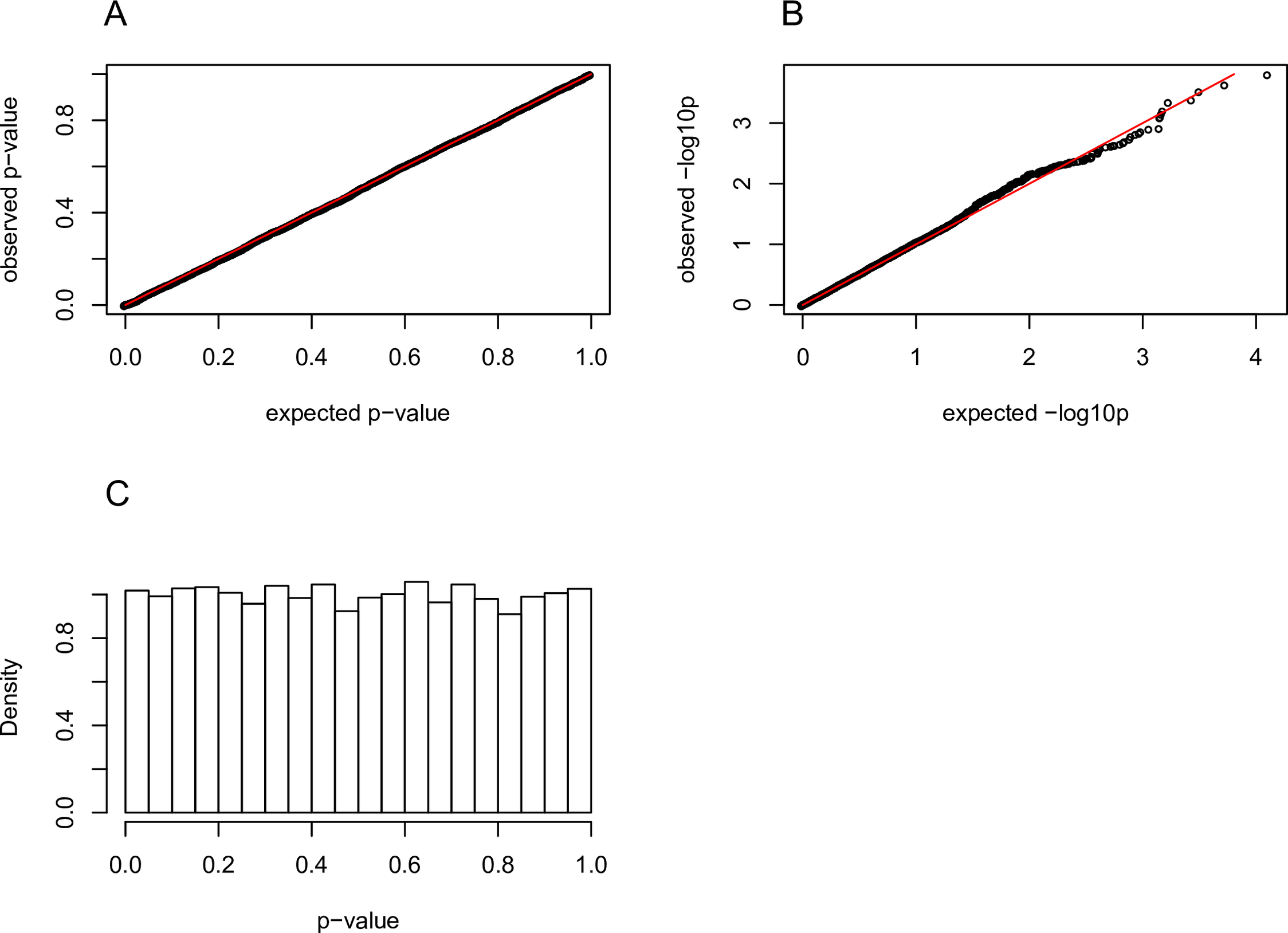

Supplement: S11 Fig — Simulation method is outlined in S1 Appendix, and the null case considered is Case 1 described in S6 Table where there is no signal for either GWAS or eQTL. In total, 104 replications were simulated to obtain (A) QQ-plot of the SS colocalization p-value on the original scale, (B) QQ-plot of the SS colocalization p-value on the −log10 scale, and (C) the histogram of the SS colocalization p-value that is expected to follow a Unif(0,1) distribution under the null. The empirical Type 1 error is 0.0509 at the 0.05 nominal level, and 0.0051 at the 0.005 level. (TIF) [file pgen.1008007.s012.tif]

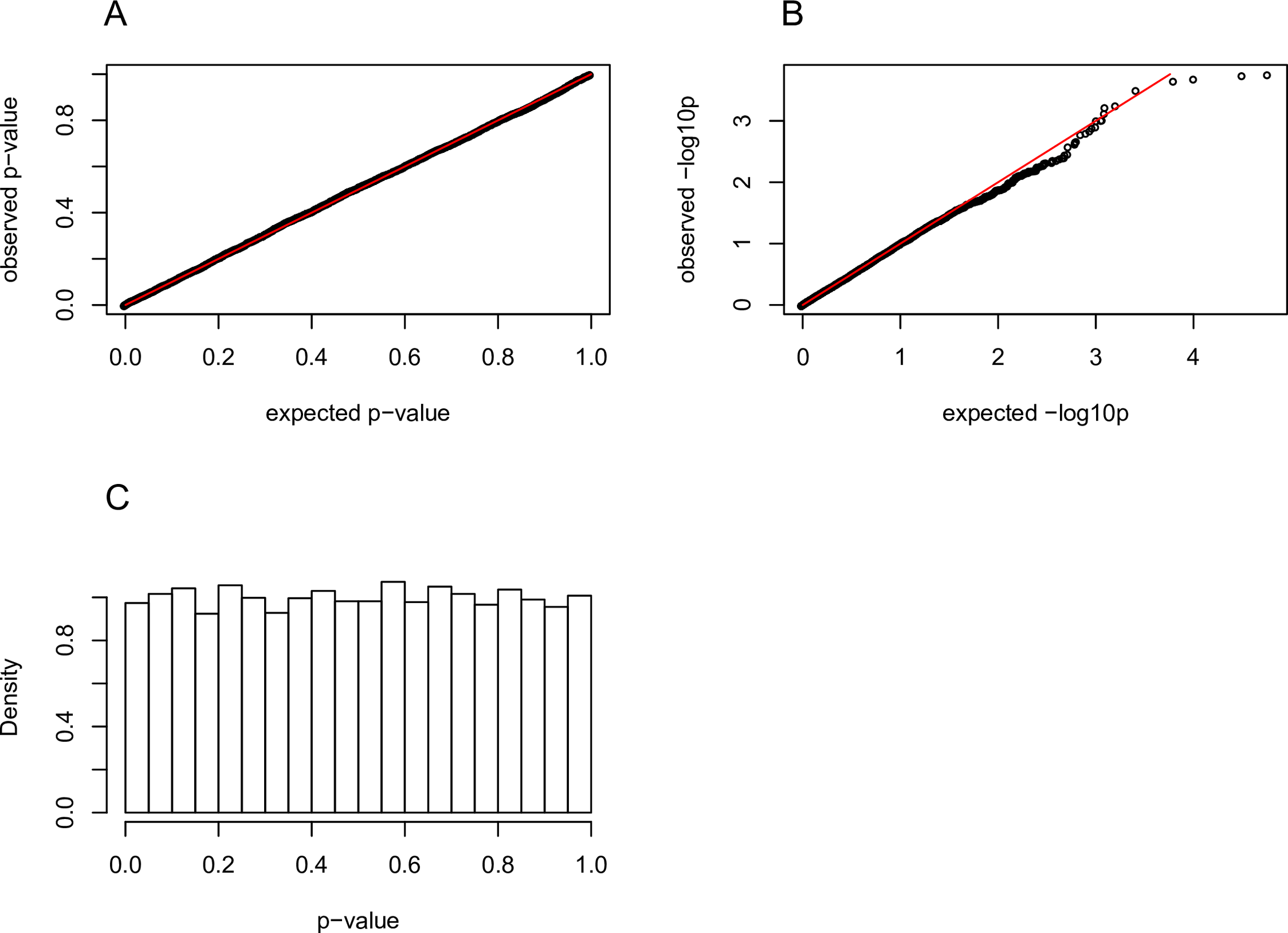

Supplement: S12 Fig — Simulation method is outlined in S1 Appendix, and the null case considered is Case 1 described in S6 Table where there is no signal for either GWAS or eQTL. In total, 104 replications were simulated to obtain (A) QQ-plot of the SS colocalization p-value on the original scale, (B) QQ-plot of the SS colocalization p-value on the −log10 scale, and (C) the histogram of the SS colocalization p-value that is expected to follow a Unif(0,1) distribution under the null. The empirical Type 1 error is 0.0487 at the 0.05 nominal level, and 0.0034 at the 0.005 level. (TIF) [file pgen.1008007.s013.tif]

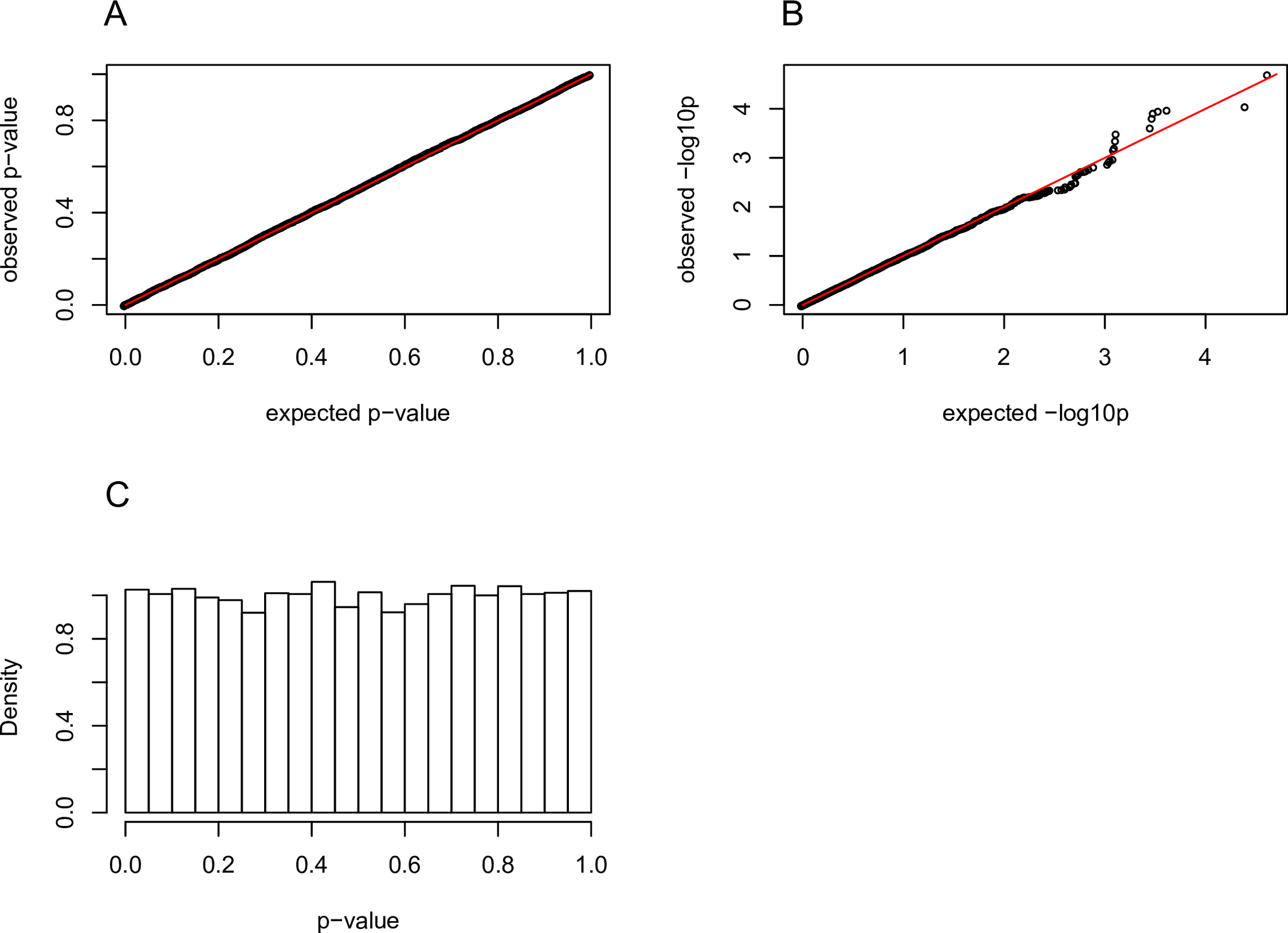

Supplement: S13 Fig — Simulation method is outlined in S1 Appendix, and the null case considered is Case 1 described in S6 Table where there is no signal for either GWAS or eQTL. In total, 104 replications were simulated to obtain (A) QQ-plot of the SS colocalization p-value on the original scale, (B) QQ-plot of the SS colocalization p-value on the −log10 scale, and (C) the histogram of the SS colocalization p-value that is expected to follow a Unif(0,1) distribution under the null. The empirical Type 1 error is 0.0513 at the 0.05 nominal level, and 0.0046 at the 0.005 level. (TIF) [file pgen.1008007.s014.tif]

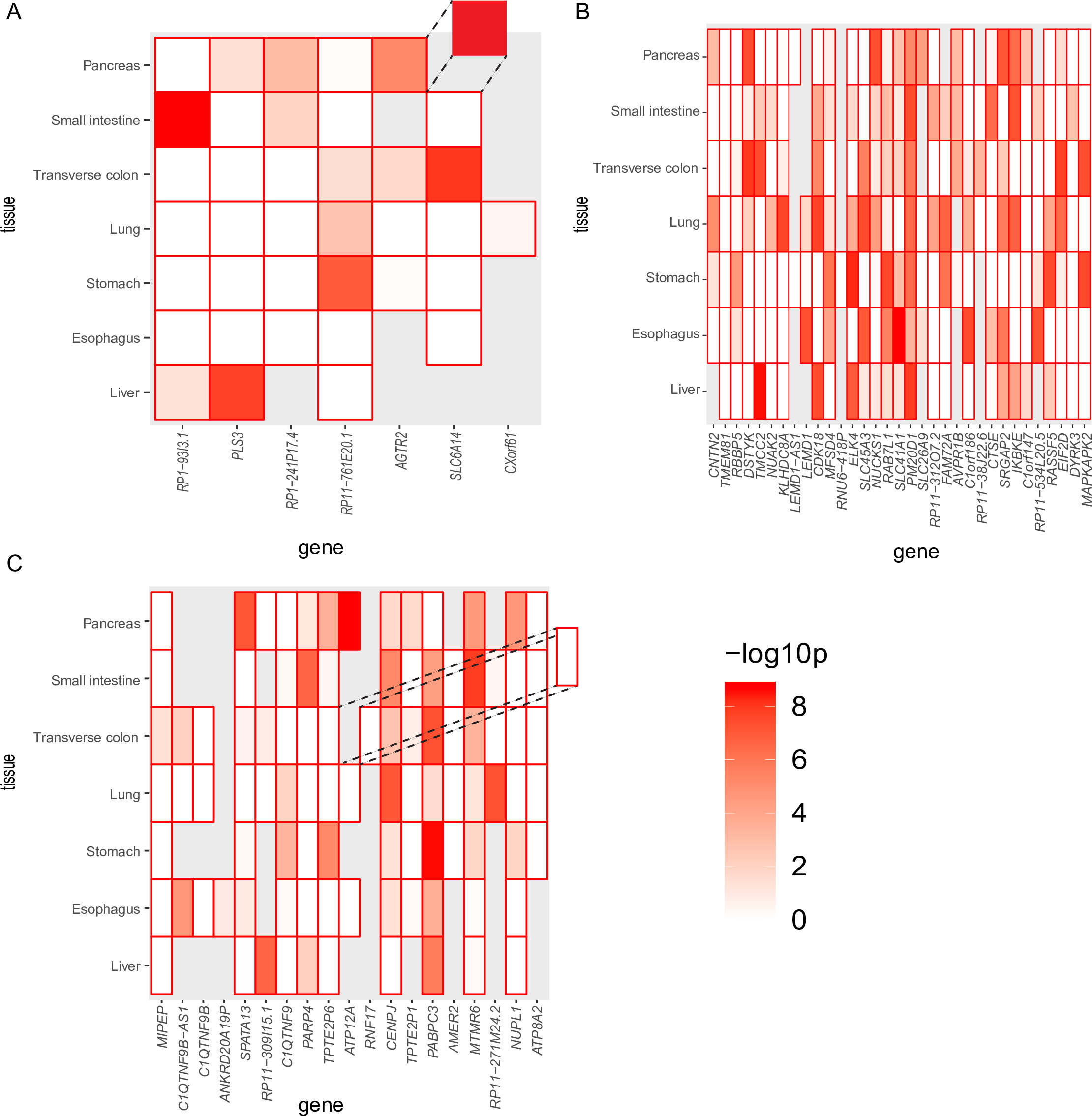

Supplement: S14 Fig — The SS colocalization test evaluates if the eQTLs for a given gene and in a given tissue colocalize with meconium ileus-associated variants in the regions of (A) chromosome X, (B) chromosome 1, and (C) chromosome 13. In each panel, each row shows the SS colocalization evidence for the specified tissue across all genes within 1Mbp of the peak GWAS variant. SS colocalization evidence for each gene is calculated for SNPs within 0.1Mbp of the peak GWAS variants; the genes on the x-axis are ordered by their chromosomal positions. Each column shows the SS colocalization evidence for the specified gene across each tissue tested. The color intensity corresponds to the SS colocalization evidence as measured by −log10(SS p-value), with red representing −log10(p) = 6 and white representing −log10(p) = 0. Grey indicates either insufficient expression levels attained for the gene in the tissue under study, or that there were no significant eQTLs for the gene in that tissue. The eQTL analyses used for all gene/tissue pairs are those conducted by GTEx version 7 release, except the boxes indicated on the margins. eQTL analysis for the boxes on the margins were calculated in version 6 but were not calculated in GTEx version 7 due to a more stringent expression threshold criteria set in GTEx v7 versus v6 (see Material and Methods for specifics); these analyses were conducted using the publicly available expression matrix (phe000020.v1) and genotypes from WGS (phg000830.v1) following GTEx’s protocol for eQTL analysis. (TIF) [file pgen.1008007.s015.tif]

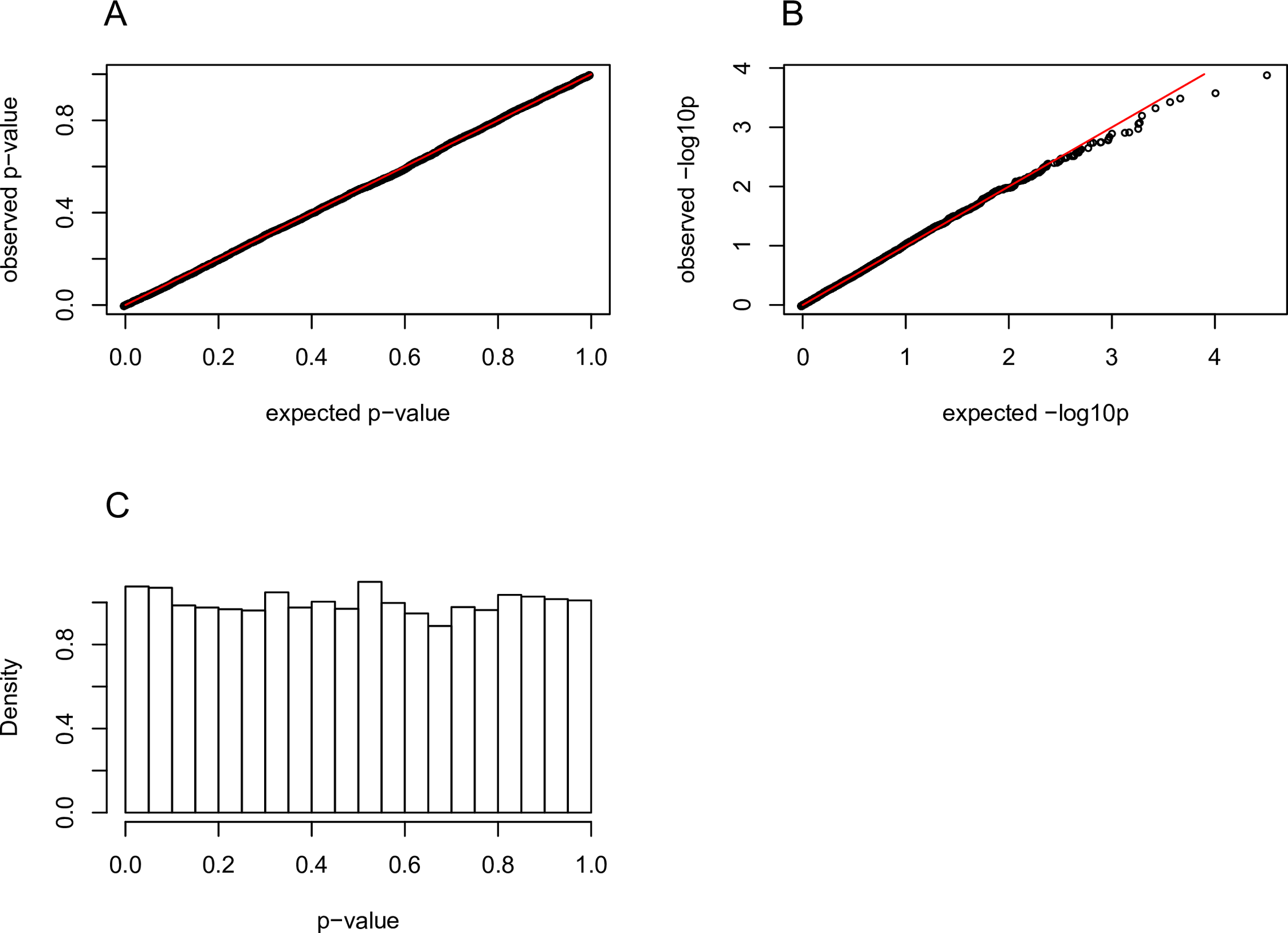

Supplement: S15 Fig — Simulation method is outlined in S1 Appendix, and the null case considered is Case 1 described in S6 Table where there is no signal for either GWAS or eQTL. In total, 104 replications were simulated to obtain (A) QQ-plot of the simple sum contrasting (SSC) colocalization p-value on the original scale, (B) QQ-plot of the SSC colocalization p-value on the −log10 scale, and (C) the histogram of the SSC colocalization p-value that is expected to follow a Unif(0,1) distribution under the null hypothesis. The empirical Type 1 Error is 0.0536 at the 0.05 nominal level, and 0.0048 at the 0.005 level. (TIF) [file pgen.1008007.s016.tif]

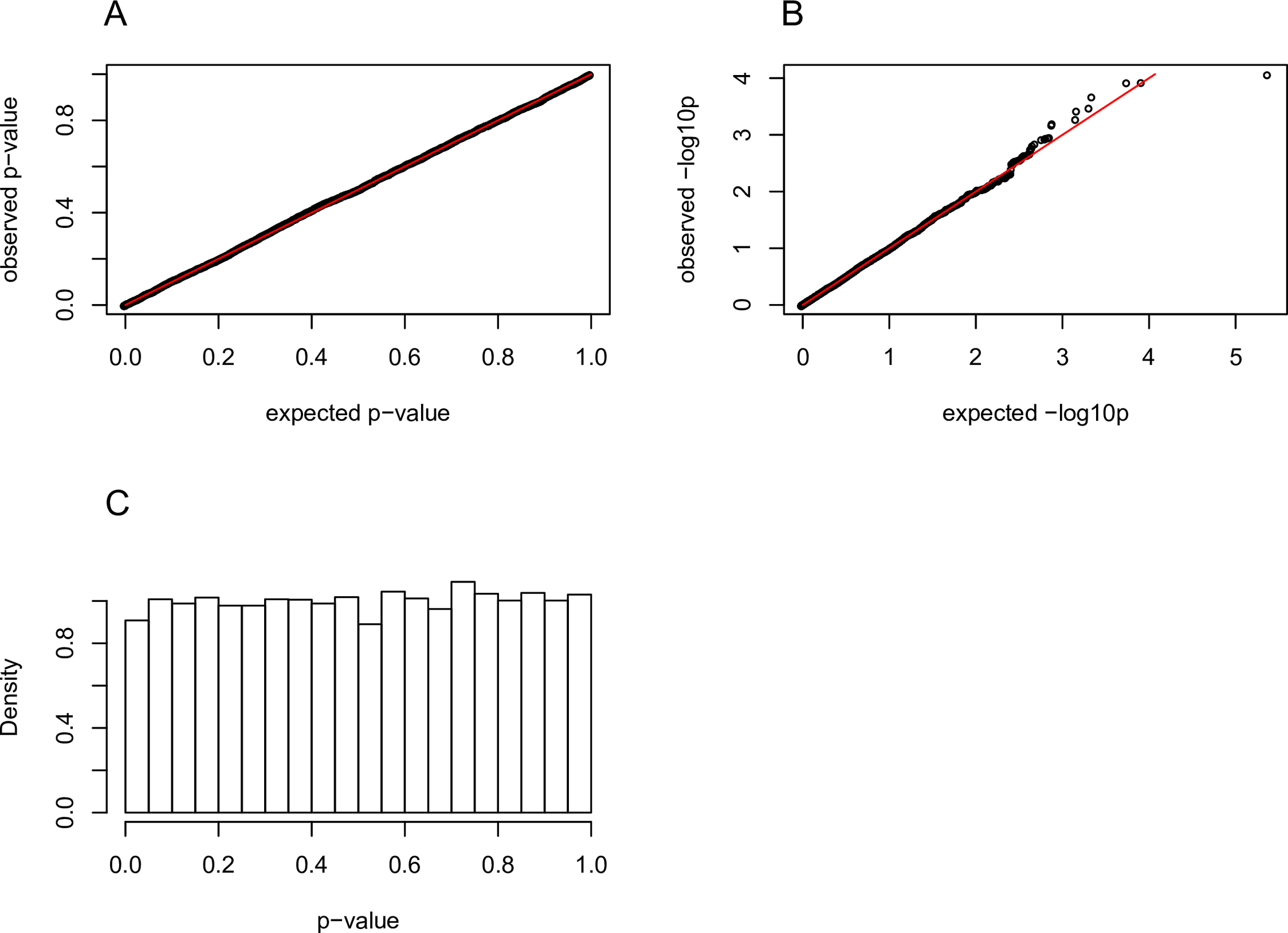

Supplement: S16 Fig — Simulation method is outlined in S1 Appendix, and the null case considered is Case 1 described in S6 Table where there is no signal for either GWAS or eQTL. In total, 104 replications were simulated to obtain (A) QQ-plot of the simple sum contrasting (SSC) colocalization p-value on the original scale, (B) QQ-plot of the SSC colocalization p-value on the −log10 scale, and (C) the histogram of the SSC colocalization p-value that is expected to follow a Unif(0,1) distribution under the null hypothesis. The empirical Type 1 error is 0.0454 at the 0.05 nominal level, and 0.0043 at the 0.005 level. (TIF) [file pgen.1008007.s017.tif]

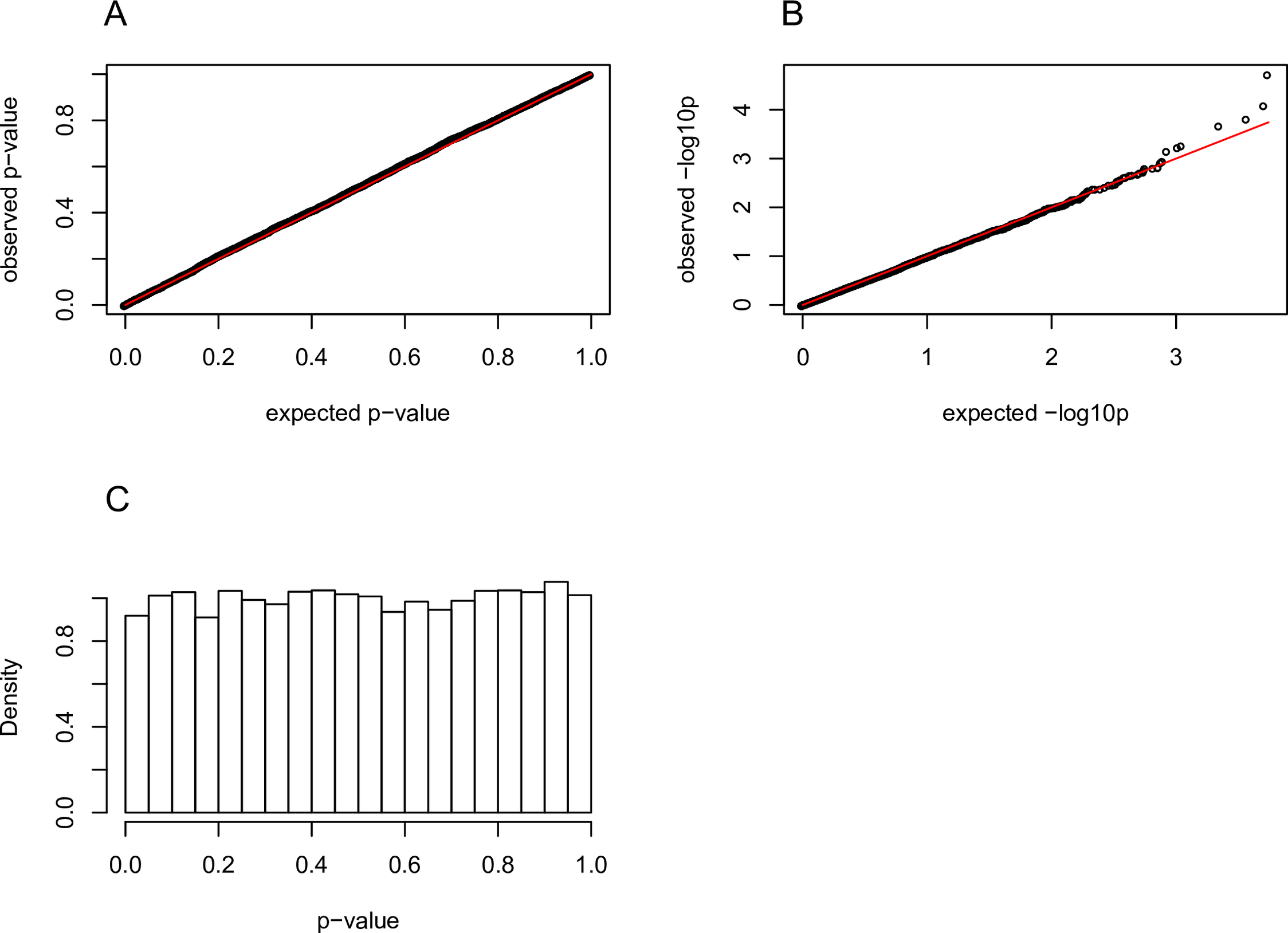

Supplement: S17 Fig — Simulation method is outlined in S1 Appendix, and the null case considered is Case 1 described in S6 Table where there is no signal for either GWAS or eQTL. In total, 104 replications were simulated to obtain (A) QQ-plot of the simple sum contrasting (SSC) colocalization p-value on the original scale, (B) QQ-plot of the SSC colocalization p-value on the −log10 scale, and (C) the histogram of the SSC colocalization p-value that is expected to follow a Unif(0,1) distribution under the null hypothesis. The empirical Type 1 error is 0.0457 at the 0.05 nominal level, and 0.0039 at the 0.005 level. (TIF) [file pgen.1008007.s018.tif]

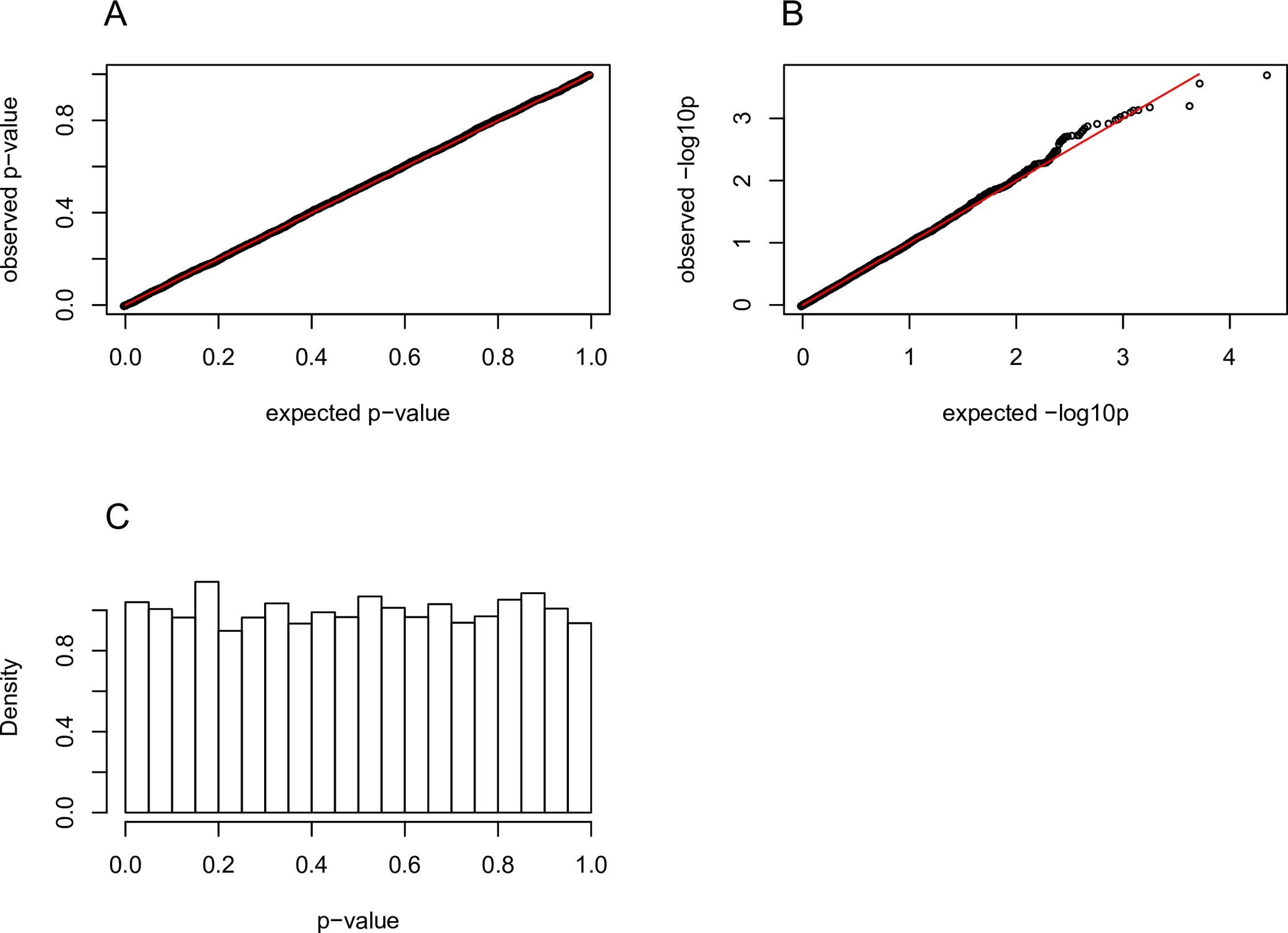

Supplement: S19 Fig — Simulation method is outlined in S1 Appendix, and the null case considered is Case 1 described in S6 Table where there is no signal for either GWAS or eQTL. In total, 104 replications were simulated to obtain (A) QQ-plot of the simple sum contrasting (SSC) colocalization p-value on the original scale, (B) QQ-plot of the SSC colocalization p-value on the −log10 scale, and (C) the histogram of the SSC colocalization p-value that is expected to follow a Unif(0,1) distribution under the null hypothesis. The empirical Type 1 error is 0.052 at the 0.05 nominal level, and 0.0048 at the 0.005 level. (TIF) [file pgen.1008007.s020.tif]

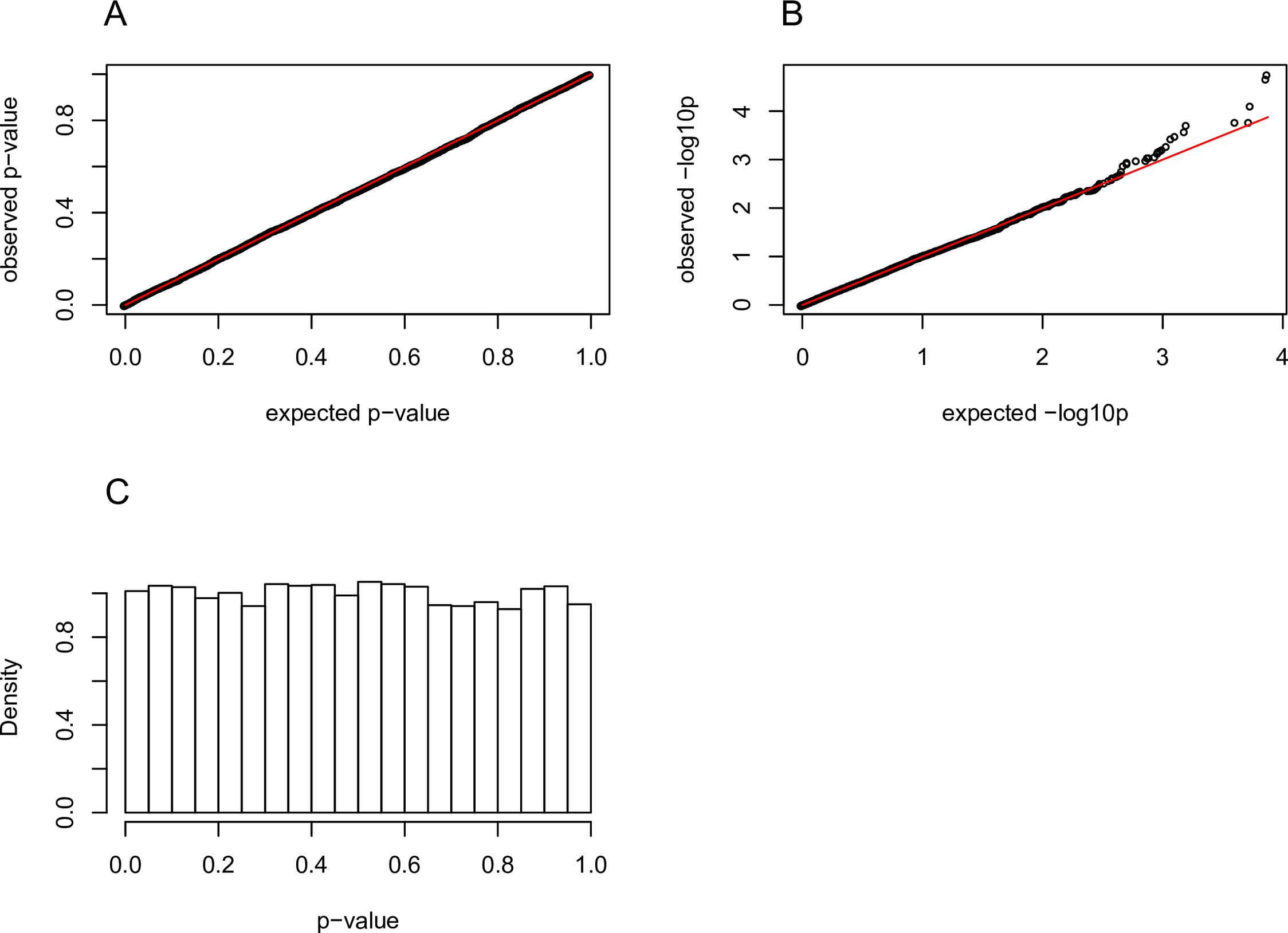

Supplement: S20 Fig — Simulation method is outlined in S1 Appendix, and the null case considered is Case 1 described in S6 Table where there is no signal for either GWAS or eQTL. In total, 104 replications were simulated to obtain (A) QQ-plot of the simple sum contrasting (SSC) colocalization p-value on the original scale, (B) QQ-plot of the SSC colocalization p-value on the −log10 scale, and (C) the histogram of the SSC colocalization p-value that is expected to follow a Unif(0,1) distribution under the null hypothesis. The empirical Type 1 error is 0.0503 at the 0.05 nominal level, and 0.0051 at the 0.005 level. (TIF) [file pgen.1008007.s021.tif]
