# Supplementary material for: Genetic association and transcriptome integration identify contributing genes and tissues at cystic fibrosis modifier loci
Source: PLoS Genet. 2019 Feb 26;15(2):e1008007. doi: 10.1371/journal.pgen.1008007 (PMC6407791; doi:10.1371/journal.pgen.1008007)
Supplement: S1 Table — The total number of individuals used for the meconium ileus association analysis after quality control of both genotypes and phenotypes, stratified by consortium site and Illumina genotyping platform. (DOCX) [file pgen.1008007.s022.docx]

**S1 Table. Total sample after quality control.** The total number of individuals used for the meconium ileus association analysis after quality control of both genotypes and phenotypes, stratified by consortium site and Illumina genotyping platform.

| Consortium  Site | Phase I-NA | Phase II-NA | | | FGMS | | Total |
| --- | --- | --- | --- | --- | --- | --- | --- |
|  | 610Quad | 660W-NA | 660W-JHU | Omni5 | CNV370 | 660W-FR |  |
| CGMS | 1519 | 278 | - | 60 | - | - | 1857 |
| JHU | 1042 | 461 | 163 | 76 | - | - | 1742 |
| UNC/Case | 1303 | 492 | - | 110 | - | - | 1905 |
| FGMS | - | - | - | - | 283 | 983 | 1266 |
| Total | 3864 | 1231 | 163 | 246 | 283 | 983 | 6770 |
