# Supplementary material for: Genetic association and transcriptome integration identify contributing genes and tissues at cystic fibrosis modifier loci
Source: PLoS Genet. 2019 Feb 26;15(2):e1008007. doi: 10.1371/journal.pgen.1008007 (PMC6407791; doi:10.1371/journal.pgen.1008007)
Supplement: S2 Table — The number of SNPs before quality control (QC) and the number of SNPs excluded by each stated QC criterion, stratified by consortium sites and Illumina genotyping platform. (DOCX) [file pgen.1008007.s023.docx]

**S2 Table. SNP Quality Control Steps**. The number of SNPs before quality control (QC) and the number of SNPs excluded by each stated QC criterion, stratified by consortium sites and Illumina genotyping platform.

| Platform | SNPs before QC | SNPs not annotated | Duplicated variants | Call rate <=90% | X heterozygosity rate >10% in male | SNPs passing QC |
| --- | --- | --- | --- | --- | --- | --- |
| 610Quad | 570572 | 0 | 0 | 33 | 0 | 570539 |
| 660W-NA | 655214 | 67721 | 0 | 29967 | 6 | 557520 |
| 660W-JHU | 655214 | 67721 | 0 | 33271 | 0 | 554222 |
| Omni5 | 4301332 | 80572 | 4603 | 4000 | 3015 | 4209142 |
| CNV370 | 309012 | 0 | 0 | 45310 | 0 | 263702 |
| 660W-FR | 554649 | 0 | 0 | 298 | 0 | 554351 |
