# Supplementary material for: Genetic association and transcriptome integration identify contributing genes and tissues at cystic fibrosis modifier loci
Source: PLoS Genet. 2019 Feb 26;15(2):e1008007. doi: 10.1371/journal.pgen.1008007 (PMC6407791; doi:10.1371/journal.pgen.1008007)
Supplement: S3 Table — SNPs significant in either study (this study with n = 6770 and previous consortium GWAS published in Sun et al [10]) and of particular functional relevance are provided in this table. (DOCX) [file pgen.1008007.s024.docx]

**S3 Table. Comparison of meconium ileus association results between this study** **and the previous consortium GWAS.** SNPs significant in either study (this study with n=6770 and previous consortium GWAS published in Sun et al [10] ) and of particular functional relevance are provided in this table.

| chr | bp | SNP | Nearest gene | LD r^2^(D’) to the top SNP in the gene | Risk Allele | Allele freq of risk allele^a^ | Sun et al ^b^ | | This Study | |
| --- | --- | --- | --- | --- | --- | --- | --- | --- | --- | --- |
|  |  |  |  |  |  |  | OR | P | OR | P |
| X | 115566839 | rs3788766 | *SLC6A14* | NA(NA) | T | 0.61 | 1.50 | 1.28 × 10^−12^ | 1.44 | <2.2x10^-16^ |
| X | 115565881 | rs5905283 | *SLC6A14* | 0.33(0.97) | C | 0.52 | 1.34 | 1.69 × 10^−8^ | 1.33 | 6.88x10^-14^ |
| X | 115565550 | rs12839137 | *SLC6A14* | 0.37(0.89) | C | 0.76 | 1.39 | 1.20 × 10^−6^ | 1.32 | 1.93x10^-8^ |
| X | 115567075 | rs12710568 | *SLC6A14* | 0.8(1) | C | 0.64 | 1.48 | 4.60 × 10^−11^ | 1.42 | 9.99x10^-16^ |
| X | 115573677 | rs5905177 | *SLC6A14* | 0.94(0.99) | T | 0.61 | 1.45 | 2.13 × 10^−10^ | 1.45 | <2.2x10^-16^ |
| 1 | 205906897 | rs7549173 | *SLC26A9* | NA(NA) | C | 0.39 | 1.41 | 6.24 × 10^−8^ | 1.37 | 2.81x10^–11^ |
| 1 | 205914757 | rs4077468 | *SLC26A9* | 0.43(0.98) | T | 0.59 | 1.45 | 9.88 × 10^−9^ | 1.35 | 2.08x10^-10^ |
| 1 | 205899595 | rs7512462 | *SLC26A9* | 0.42(0.97) | T | 0.59 | 1.45 | 2.14 × 10^−8^ | 1.34 | 1.86x10^-9^ |
| 1 | 205912859 | rs1342063 | *SLC26A9* | 0.44(0.99) | C | 0.59 | 1.45 | 1.05 × 10^−8^ | 1.35 | 2.10x10^-10^ |
| 1 | 205917309 | rs7419153 | *SLC26A9* | 0.66(-0.85) | T | 0.38 | 1.42 | 1.01 × 10^−7^ | 1.37 | 6.82x10^-11^ |
| 1 | 205916699 | rs12047830 | *SLC26A9* | 0.65(0.99) | C | 0.51 | 1.34 | 3.72 × 10^−6^ | 1.30 | 1.72x10^-8^ |
| 13 | 25282819 | rs61948108 | *ATP12A* | NA(NA) | T | 0.11 | 1.47 | 1.72x10^-5^ | 1.55 | 3.83x10^-10^ |
| 13 | 25283494 | rs895423 | *ATP12A* | 0.95(0.97) | T | 0.11 | 1.47 | 1.72x10^-5^ | 1.54 | 6.17x10^-10^ |
| 7 | 142455538 | rs3757377 | *PRSS1* | NA(NA) | T | 0.39 | 1.37 | 1.16x10^-6^ | 1.29 | 2.55x10^-7^ |
| 7 | 142456928 | rs10273639 | *PRSS1* | 0.71(-0.99) | T | 0.43 | 1.36 | 2.10x10^-6^ | 1.28 | 3.83x10^-7^ |
| 7 | 142457132 | rs4726576 | *PRSS1* | 0.58(-0.82) | T | 0.43 | 1.36 | 2.10x10^-6^ | 1.28 | 4.80x10^-7^ |
| 7 | 142498523 | rs1799886 | *PRSS1* | <0.2(<0.2) | T | 0.55 | 1.26 | 3.30x10^-4^ | 1.33 | 1.7410^-7^ |

^a^ Allele frequency of the risk allele is based on the whole sample

^b^ Results from Sun et al [10] had a sample size of n=3,763, which is a subset of the Phase I-NA (n=3,864) as described in the S1 Table of this study; this is because the current analysis includes a few additional samples with updated meconium ilues status.
