# Supplementary material for: Genetic association and transcriptome integration identify contributing genes and tissues at cystic fibrosis modifier loci
Source: PLoS Genet. 2019 Feb 26;15(2):e1008007. doi: 10.1371/journal.pgen.1008007 (PMC6407791; doi:10.1371/journal.pgen.1008007)
Supplement: S4 Table — The three variants here include the top two ranked SNPs, rs3788766 and rs5905177 as in S3 Table, and putative functional variant rs12710568. (DOCX) [file pgen.1008007.s025.docx]

**S4 Table. Sex-specific association analysis of variants in *SLC6A14*.** The three variants here include the top two ranked SNPs, rs3788766 and rs5905177 as in S3 Table, and putative functional variant rs12710568**.**

| SNP | Risk Allele | Risk Allele Frequency | | Sex-specific analysis | | P-value | OR (95% CI) |
| --- | --- | --- | --- | --- | --- | --- | --- |
|  | (+) | MI | non-MI |  |  |  |  |
| rs3788766 | A | 0.71 | 0.59 | Female only (454:1479:1238) | Additive | 2.27x10^-06^ | 1.42 (1.23, 1.64) |
|  |  |  |  |  | Genotypic (2df) | 1.50x10^-06^ | - |
|  |  |  |  |  | AA vs AG | 5.19x10^-06^ | 1.59 (1.30, 1.94) |
|  |  |  |  |  | AA vs GG | 5.19x10^-06^ | 1.80 (1.33, 2.45) |
|  |  |  |  | Male only (1419:2180) | A vs G | 2.41x10^-13^ | 2.16 (1.76, 2.66) |
|  |  |  |  | Female and Male | Additive (0/1/2 Female; 0/2 Male) | <2.2x10^-16^ | 1.44 (1.33,1.57) |
|  |  |  |  |  |  |  |  |
|  |  |  |  |  |  |  |  |
|  |  |  |  |  | Female vs Male effect difference^b^ | 0.074 | 1.17^b^ (0.98, 1.39) |
| rs5905177^a^ | T | 0.73 | 0.61 | Female only (428:1456:1287) | Additive | 1.50x10^-07^ | 1.43 (1.24, 1.66) |
|  |  |  |  |  | Genotypic (2df) | 8.57x10^-07^ | - |
|  |  |  |  |  | TT vs TC | 2.94x10^-07^ | 1.61 (1.32, 1.96) |
|  |  |  |  |  | TT vs CC | 2.01x10^-04^ | 1.81 (1.32, 2.48) |
|  |  |  |  | Male only (1359:2224) | T vs C | 2.55x10^-12^ | 2.11 (1.71, 2.61) |
|  |  |  |  | Female and Male | Additive (0/1/2 Female; 0/2 Male) | <2.2x10^-16^ | 1.45 (1.33, 1.57) |
|  |  |  |  |  |  |  |  |
|  |  |  |  |  |  |  |  |
|  |  |  |  |  | Female vs Male effect difference^b^ | 0.046 | 1.19^b^ (1.00, 1.41) |
| rs12710568 | C | 0.75 | 0.64 | Female only (362:1365:1444) | Additive | 1.54x10^-06^ | 1.44 (1.24, 1.67) |
|  |  |  |  |  | Genotypic (2df) | 4.44x10^-06^ | - |
|  |  |  |  |  | CC vs CG | 4.43x10^-05^ | 1.51 (1.24, 1.83) |
|  |  |  |  |  | CC vs GG | 2.08x10^-04^ | 1.94 (1.37, 2.76) |
|  |  |  |  | Male only (1248:2334) | C vs G | 2.70x10^-10^ | 1.99 (1.61, 2.47) |
|  |  |  |  | Female and Male | Additive (0/1/2 Female; 0/2 Male) | 9.99x10^-16^ | 1.42 (1.31, 1.55) |
|  |  |  |  |  |  |  |  |
|  |  |  |  |  |  |  |  |
|  |  |  |  |  | Female vs Male effect difference^b^ | 0.058 | 1.17^b^ (0.99, 1.38) |

^a^LD r^2^=0.94 with rs3788766 as in S3 Table.

^b^Testing for female and male effect size differences using contrast test under the assumption of X-inactivation, and equal meconium ileus frequency in non-risk allele carriers. Details of the test are in the *Chromosome X analysis* subsection of Material and Methods.
