# Supplementary material for: Genetic association and transcriptome integration identify contributing genes and tissues at cystic fibrosis modifier loci
Source: PLoS Genet. 2019 Feb 26;15(2):e1008007. doi: 10.1371/journal.pgen.1008007 (PMC6407791; doi:10.1371/journal.pgen.1008007)
Supplement: S5 Table — Values of standardized true effect size of the associated SNP for GWAS or an eQTL used in the various simulation settings, with the corresponding GWAS or eQTL power of individually detecting the SNP at the 10−8 significance level, and the expected -log10(p-value) of the GWAS association or the eQTL analysis if the observed signal strength is the true effect size. See S1 Appendix for other simulation details. (DOCX) [file pgen.1008007.s026.docx]

**S5 Table:** **Parameter values for the simulation studies.**  Values of standardized true effect size of the associated SNP for GWAS or an eQTL used in the various simulation settings, with the corresponding GWAS or eQTL power of individually detecting the SNP at the 10^-8^ significance level, and the expected -log10(p-value) of the GWAS association or the eQTL analysis if the observed signal strength is the true effect size. See S1 Appendix for other simulation details.

| Standardized true effect size for a GWAS SNP ($\lambda_{Z_{c}})$or an eQTL ($\lambda_{T_{c}})$ | 0 | 3.4 | 4.09 | 4.45 | 5.21 | 5.73 | 6.25 | 6.57 | 7.01 |
| --- | --- | --- | --- | --- | --- | --- | --- | --- | --- |
| Power of GWAS or eQTL detecting the SNP at 10^-8^ | 0 | 0.01 | 0.05 | 0.1 | 0.3 | 0.5 | 0.7 | 0.8 | 0.9 |
| Expected -log10 (p-value) of the GWAS SNP or the eQTL | 0 | 3.18 | 4.36 | 5.06 | 6.7 | 8 | 9.4 | 10.3 | 11.6 |
