# Supplementary material for: Genetic association and transcriptome integration identify contributing genes and tissues at cystic fibrosis modifier loci
Source: PLoS Genet. 2019 Feb 26;15(2):e1008007. doi: 10.1371/journal.pgen.1008007 (PMC6407791; doi:10.1371/journal.pgen.1008007)
Supplement: S6 Table — Plots (a)-(d) provide the general visualization of GWAS (red line) and eQTL (blue) patterns (on the -log10 p scale) in a region of interest (e.g. the SLC6A14 locus). The value of λZc represents the standardized true effect size of a GWAS associated variant, and λTc represents the true standardized effect size of an eQTL variant. The corresponding power of detecting the GWAS SNP or finding the eQTL are provided in S5 Table. For illustration purposes but without loss generality, if there was a GWAS association, λZc was set to be 5.73 such that 0.5 power could be achieved to detect the signal. If there was an eQTL from the gene-expression study, λTc was set to be 7.01 such that 0.9 power could be achieved to detect the signal. See S1 Appendix for other simulation details. (DOCX) [file pgen.1008007.s027.docx]

**S6 Table: Overview and illustration of the cases under the composite null hypothesis that there is no colocalization.** Plots (a)-(d) provide the general visualization of GWAS (red line) and eQTL (blue) patterns (on the -log10 p scale) in a region of interest (e.g. the *SLC6A14* locus). The value of $\lambda_{Z_{c}}$represents the standardized true effect size of a GWAS associated variant, and $\lambda_{T_{c}}$represents the true standardized effect size of an eQTL variant. The corresponding power of detecting the GWAS SNP or finding the eQTL are provided in S5 Table. For illustration purposes but without loss generality, if there was a GWAS association, $\lambda_{Z_{c}}$was set to be 5.73 such that 0.5 power could be achieved to detect the signal. If there was an eQTL from the gene-expression study, $\lambda_{T_{c}}$was set to be 7.01 such that 0.9 power could be achieved to detect the signal. See S1 Appendix for other simulation details.

| The Null Cases Considered | Parameter values (signal strength) | Illustration (red: GWAS, blue: eQTL) |
| --- | --- | --- |
| Case 1: NO GWAS association, and NO eQTL | $\lambda_{Z_{c}}$= 0, $\lambda_{T_{c}}=0$ | (a)   |
| Case 2: NO GWAS association, and YES eQTL | $\lambda_{Z_{c}}$= 0, $\lambda_{T_{c}}=$7.01 | ­­­­­­­(b)   |
| Case 3: YES GWAS association, and NO eQTL | $\lambda_{Z_{c}}$= 5.73, $\lambda_{T_{c}}=0$ | (c)   |
| Case 4: YES GWAS association, and YES eQTL, but signal occurring at two independent SNPs | $\lambda_{Z_{c}}$=5.73, $\lambda_{T_{c}}=7.01$ | (d)   |
