# Supplementary material for: Genetic association and transcriptome integration identify contributing genes and tissues at cystic fibrosis modifier loci
Source: PLoS Genet. 2019 Feb 26;15(2):e1008007. doi: 10.1371/journal.pgen.1008007 (PMC6407791; doi:10.1371/journal.pgen.1008007)
Supplement: S7 Table — Plots (a)-(f) provide the general visualization of GWAS (red line) and eQTL (blue) patterns (on the -log10 p scale) in a region of interest (e.g. the SLC6A14 locus). The parameter values are varied in the power study, thus they are provided in the corresponding referred tables. (DOCX) [file pgen.1008007.s028.docx]

**S7 Table: Overview with illustration of the cases under different types of alternatives when there is colocalization at at least one variant in the region.** Plots (a)-(f) provide the general visualization of GWAS (red line) and eQTL (blue) patterns (on the -log10 p scale) in a region of interest (e.g. the *SLC6A14* locus). The parameter values are varied in the power study, thus they are provided in the corresponding referred tables.

| Alternatives for Different Types of GWAS-eQTL Colocalization | | | Illustration (red: GWAS, blue: eQTL) |
| --- | --- | --- | --- |
| ONE shared or overlapping SNP | Alter1: ONE GWAS SNP, and ONE eQTL (Refer to S11 Table for power) | | (a)   |
|  | TWO independent GWAS SNPs, and ONE eQTL | Alter2: The eQTL peak overlapped with the higher GWAS peak  (Refer to S12 Table for power) | (b)   |
|  |  | Alter3: The eQTL peak overlapped with the lower GWAS peak  (Refer to S13 Table for power) | (c)   |
|  | ONE GWAS SNP, and TWO independent eQTLs | Alter4: The non-overlapped eQTL peak is lower than the GWAS peak  (Refer to S14 Table for power) | (d)   |
|  |  | Alter5: The non-overlapped eQTL peak is higher than the GWAS peak  (Refer to S15 Table for power) | (e)   |
| TWO shared or overlapping SNPs | Alter6: TWO overlapping GWAS SNPs and eQTLs  (Refer to S16 Table for power) | | (f)   |
