# Supplementary material for: Genetic association and transcriptome integration identify contributing genes and tissues at cystic fibrosis modifier loci
Source: PLoS Genet. 2019 Feb 26;15(2):e1008007. doi: 10.1371/journal.pgen.1008007 (PMC6407791; doi:10.1371/journal.pgen.1008007)
Supplement: S8 Table — The null cases considered are detailed in S6 Table. The LD pattern at the simulated region follows that at the SLC6A14 locus. For the SS method, the nominal type 1 error was set at alpha = 0.05 or alpha = 0.005. The eQTL evidence was measured continuously as -log10 (eQTL p-value), or dichotomized using the eQTL p<0.05 or <0.005 threshold. For COLOC and eCAVIAR, the false positive rates were calculated by applying the 0.5, 0.75 or 0.9 threshold (as in [1]) to the colocalization posterior probability. In total, 104 replications were simulated to obtain each cell of the table. See S1 Appendix for other simulation details. (DOCX) [file pgen.1008007.s029.docx]

**S8 Table: Type 1 error evaluation of the proposed Simple Sum colocalization analytical method, and the false positive rate of COLOC and eCAVIAR under the different null cases.** The null cases considered are detailed in S6 Table. The LD pattern at the simulated region follows that at the *SLC6A14* locus. For the SS method, the nominal type 1 error was set at alpha=0.05 or alpha= 0.005. The eQTL evidence was measured continuously as -log10 (eQTL p-value), or dichotomized using the eQTL p<0.05 or <0.005 threshold. For COLOC and eCAVIAR, the false positive rates were calculated by applying the 0.5, 0.75 or 0.9 threshold (as in [1]) to the colocalization posterior probability. In total, 10^4^ replications were simulated to obtain each cell of the table. See S1 Appendix for other simulation details.

| The Null Cases Considered | Type 1 error of the proposed Simple Sum colocalization analytical method | | | | | | False positive rate of COLOC | | | False positive rate of eCAVIAR | | |
| --- | --- | --- | --- | --- | --- | --- | --- | --- | --- | --- | --- | --- |
|  | -log10(eQTL p): alpha=0.05 | eQTL p<0.05: alpha=0.05 | eQTL p<0.005: alpha=0.05 | -log10(eQTL p): alpha=0.005 | eQTL p<0.05: alpha=0.005 | eQTL p<0.005: alpha=0.005 | cut off =0.5 | cut off =0.75 | cut off =0.90 | cut off =0.5 | cut off =0.75 | cut off =0.90 |
| Case 1: NO GWAS association, and NO eQTL | 0.0501 | 0.0478 | 0.0522 | 0.0053 | 0.0049 | 0.0041 | <10^-4^ | <10^-4^ | <10^-4^ | <10^-4^ | <10^-4^ | <10^-4^ |
| Case 2: NO GWAS association, and YES eQTL | 0.0476 | 0.0458 | 0.0472 | 0.0069 | 0.0066 | 0.007 | 0.0033 | 0.0001 | <10^-4^ | <10^-4^ | <10^-4^ | <10^-4^ |
| Case 3: YES GWAS association, and NO eQTL | 0.2802 | 0.1823 | 0.0578 | 0.1796 | 0.1129 | 0.0307 | 0.003 | 0.0006 | 0.0001 | <10^-4^ | <10^-4^ | <10^-4^ |
| Case 4: YES GWAS association, and YES eQTL, with the signals occurring at two independent SNPs | 0.0148 | 0.0259 | 0.0153 | 0.0016 | 0.0048 | 0.0017 | 0.0003 | 0.0000 | <10^-4^ | <10^-4^ | <10^-4^ | <10^-4^ |
