# Supplementary material for: Genetic association and transcriptome integration identify contributing genes and tissues at cystic fibrosis modifier loci
Source: PLoS Genet. 2019 Feb 26;15(2):e1008007. doi: 10.1371/journal.pgen.1008007 (PMC6407791; doi:10.1371/journal.pgen.1008007)
Supplement: S9 Table — All the null cases considered are detailed in S6 Table. Case 3 is the situation when there is a GWAS associated SNP but there is no eQTL (plot (c) in S6 Table). In that case, colocalization results should be interpreted with caution if the observed eQTL signal is weak with examples demonstrated below (i.e. the null hypothesis is not rejected if the maximum of -log10 (eQTL p-value) is below a threshold (column 1)). Among the remaining replicates, the null is rejected if the Simple Sum colocalization p-value is smaller than the nominal type 1 error level (alpha = 0.05 or alpha = 0.005). The LD pattern at the simulated region follows that at the SLC6A14 locus. In total, 104 replications were simulated to obtain each cell of the table. See S1 Appendix for other simulation details. (DOCX) [file pgen.1008007.s030.docx]

**S9 Table.** **Type 1 error evaluation of the proposed Simple Sum colocalization analytical method under the null Case 3.** All the null cases considered are detailed in S6 Table. Case 3 is the situation when there is a GWAS associated SNP but there is no eQTL (plot (c) in S6 Table). In that case, colocalization results should be interpreted with caution if the observed eQTL signal is weak with examples demonstrated below (i.e. the null hypothesis is not rejected if the maximum of -log10 (eQTL p-value) is below a threshold (column 1)). Among the remaining replicates, the null is rejected if the Simple Sum colocalization p-value is smaller than the nominal type 1 error level (alpha=0.05 or alpha=0.005). The LD pattern at the simulated region follows that at the *SLC6A14* locus. In total, 10^4^ replications were simulated to obtain each cell of the table. See S1 Appendix for other simulation details.

| Null Case 3  Threshold used for eQTL before performing colocalization analysis | Type I error | | | | | |
| --- | --- | --- | --- | --- | --- | --- |
|  | -log10(eQTL p):  alpha=0.05 | eQTL p<0.05:  alpha=0.05 | eQTL p<0.005:  alpha=0.05 | -log10(eQTL p):  alpha=0.005 | eQTL p<0.05:  alpha=0.005 | eQTL p<0.005:  alpha=0.005 |
| Threshold =2.5 | 0.0991 | 0.0714 | 0.0445 | 0.0626 | 0.0461 | 0.0242 |
| Threshold =2.7 | 0.0704 | 0.0518 | 0.0325 | 0.0449 | 0.0330 | 0.0176 |
| Threshold =2.9 | 0.0478 | 0.0362 | 0.0229 | 0.0310 | 0.0236 | 0.0129 |
| Threshold =3.0 | 0.0388 | 0.0302 | 0.0195 | 0.0250 | 0.0193 | 0.0109 |
| Threshold =3.1 | 0.0309 | 0.0247 | 0.0159 | 0.0200 | 0.0156 | 0.0097 |
| Threshold =3.3 | 0.0207 | 0.0171 | 0.0115 | 0.0136 | 0.0112 | 0.0071 |
| Threshold =3.5 | 0.0126 | 0.0096 | 0.0073 | 0.0080 | 0.0064 | 0.0043 |
| Threshold =3.7 | 0.0077 | 0.0059 | 0.0046 | 0.0052 | 0.0038 | 0.0028 |
| Threshold =3.9 | 0.0048 | 0.0035 | 0.0028 | 0.0032 | 0.0023 | 0.0018 |
| Threshold =4.0 | 0.0041 | 0.0029 | 0.0025 | 0.0026 | 0.0018 | 0.0015 |
