# Supplementary material for: Genetic association and transcriptome integration identify contributing genes and tissues at cystic fibrosis modifier loci
Source: PLoS Genet. 2019 Feb 26;15(2):e1008007. doi: 10.1371/journal.pgen.1008007 (PMC6407791; doi:10.1371/journal.pgen.1008007)
Supplement: S10 Table — The eQTL evidence was dichotomized by using thresholds of eQTL p<0.05, <0.005 or <0.0005 instead of based on the -log10(p-value) as in Table 3. Analytical and permutation-based (# of replicates = 105) Simple Sum (SS) colocalization p-values evaluate if the eQTLs for a given gene and tissue colocalize with meconium ileus-associated variants. All colocalization p-values were one-sided because only positive association implies eQTL-association colocalization (i.e. eQTL peaks coincide with association peaks). Simple Sum Contrasting (SSC) colocalization p-value evaluates if the eQTLs in the pancreas colocalize with meconium ileus-associated variants more than eQTLs in another tissue; NAs are listed for the pancreas since we do not contrast pancreas with itself. Other NAs are used when there are no SNPs with eQTLs p less than the thresholds considered (0.05, 0.005 or 0.0005) for that gene and tissue. (DOCX) [file pgen.1008007.s031.docx]

**S10 Table. Results of Simple Sum colocalization and contrasting colocalization analyses for the three loci genome-wide significantly associated with meconium ileus.** The eQTL evidence was dichotomized by using thresholds of eQTL p<0.05, <0.005 or <0.0005 instead of based on the -log10(p-value) as in Table 3. Analytical and permutation-based (# of replicates=10^5^) Simple Sum *(SS)* colocalization p-values evaluate if the eQTLs for a given gene and tissue colocalize with meconium ileus-associated variants. All colocalization p-values were one-sided because only positive association implies eQTL-association colocalization (i.e. eQTL peaks coincide with association peaks). Simple Sum Contrasting (*SSC*) colocalization p-value evaluates if the eQTLs in the pancreas colocalize with meconium ileus-associated variants more than eQTLs in another tissue; NAs are listed for the pancreas since we do not contrast pancreas with itself. Other NAs are used when there are no SNPs with eQTLs p less than the thresholds considered (0.05, 0.005 or 0.0005) for that gene and tissue.

| Gene | Tissue | SS colocalization for eQTL p<0.05 | | SS colocalization for eQTL p<0.005 | | SS colocalization for eQTL p<0.0005 | | SSC Colocalization p-value (Pancreas vs other tissue, eQTL p<0.05) |
| --- | --- | --- | --- | --- | --- | --- | --- | --- |
|  |  | Analytical  p-value | Permutation p-value | Analytical p-value | Permutation p-value | Analytical p-value | Permutation p-value |  |
| SLC6A14 | Pancreas | 2.99 x10^-8^ | 0 | 9.22 x10^-9^ | 0 | 4.69 x10^-9^ | 0 | NA |
|  | Esophagus | 1 | 1 | 1 | 1 | 1 | 0.98 | 1.22 x10^-8^ |
|  | Transverse Colon | 5.25 x10^-9^ | 0 | 6.99 x10^-9^ | 0 | 1 | 0.98 | 0.0036 |
|  | Stomach | 0.998 | 0.998 | 1 | 1 | 1 | 0.98 | 4.53 x10^-8^ |
|  | Lung | 1 | 1 | 1 | 1 | 1 | 0.98 | 9.44 x10^-9^ |
| SLC26A9 | Pancreas | 1.82 x10^-7^ | 0 | 1.01 x10^-6^ | 0 | 4.64 x10^-3^ | 0.0052 | NA |
|  | Esophagus | 0.0316 | 0.0319 | 0.99 | 0.99 | 0.791 | 0.788 | 2.67 x10^-7^ |
|  | Stomach | 1 | 1 | 1 | 1 | 1 | 1 | 5.25 x10^-8^ |
|  | Lung | 0.0139 | 0.0136 | 1 | 1 | 1 | 1 | 5.54 x10^-6^ |
| ATP12A | Pancreas | 2.54 x10^-7^ | 0 | 9.58 x10^-8^ | 0 | 2.77 x10^-8^ | 0 | NA |
|  | Transverse Colon | 1 | 1 | 1 | 1 | 1 | 1 | 2.35 x10^-7^ |
|  | Esophagus | 1 | 1 | 0.998 | 0.998 | 1 | 1 | 6.15 x10^-7^ |
