# Supplementary material for: Genetic association and transcriptome integration identify contributing genes and tissues at cystic fibrosis modifier loci
Source: PLoS Genet. 2019 Feb 26;15(2):e1008007. doi: 10.1371/journal.pgen.1008007 (PMC6407791; doi:10.1371/journal.pgen.1008007)
Supplement: S11 Table — The LD pattern at the simulated region follows that at the SLC6A14 locus. For the SS method, the nominal type 1 error was set at alpha = 0.05 or alpha = 0.005. The eQTL evidence was measured continuously as -log10 (eQTL p-value), or dichotomized using the eQTL p<0.05 or <0.005 threshold. For COLOC and eCAVIAR, the false positive rates were calculated by applying the 0.5, 0.75 or 0.9 threshold to the colocalization posterior probability. The value of λZc represents the standardized true effect size of the GWAS associated variant, and λTc represents the standardized true effect size of the eQTL variant, as detailed in S5 Table. Here, λZc is set to be 5.73 such that 0.5 power is achieved to detect the GWAS association at significance level of 10−8, while λTc is set to be 3.4, 4.09, 4.45, 5.21 or 5.73 for each row of the table such that 0.01, 0.05, 0.1, 0.3, or 0.5 power is achieved to detect the eQTL association at significance level 10−8. In total, 104 replications were simulated to obtain each cell of the table. See S1 Appendix for other simulation details. (DOCX) [file pgen.1008007.s032.docx]

**S11 Table. Power evaluation of the proposed Simple Sum colocalization analytical method, and the true positive rate of COLOC and eCAVIAR, under the alternative that ONE GWAS and ONE eQTL locus colocolizes (Alter1 in S7 Table)**. The LD pattern at the simulated region follows that at the *SLC6A14* locus. For the SS method, the nominal type 1 error was set at alpha=0.05 or alpha= 0.005. The eQTL evidence was measured continuously as -log10 (eQTL p-value), or dichotomized using the eQTL p<0.05 or <0.005 threshold. For COLOC and eCAVIAR, the false positive rates were calculated by applying the 0.5, 0.75 or 0.9 threshold to the colocalization posterior probability. The value of $\lambda_{Z_{c}}$represents the standardized true effect size of the GWAS associated variant, and $\lambda_{T_{c}}$represents the standardized true effect size of the eQTL variant, as detailed in S5 Table. Here, $\lambda_{Z_{c}}$is set to be 5.73 such that 0.5 power is achieved to detect the GWAS association at significance level of 10^-8^, while $\lambda_{T_{c}}$is set to be 3.4, 4.09, 4.45, 5.21 or 5.73 for each row of the table such that 0.01, 0.05, 0.1, 0.3, or 0.5 power is achieved to detect the eQTL association at significance level 10^-8^. In total, 10^4^ replications were simulated to obtain each cell of the table. See S1 Appendix for other simulation details.

| Alter1: ONE GWAS SNP, and ONE eQTL | Power of the proposed Simple Sum colocalization analytical method | | | | | | True positive rate of COLOC | | | True positive rate of eCAVIAR | | |
| --- | --- | --- | --- | --- | --- | --- | --- | --- | --- | --- | --- | --- |
|  | -log10(eQTL p): alpha=0.05 | eQTL p<0.05:  alpha=0.05 | eQTL p<0.005:  alpha=0.05 | -log10(eQTL p): alpha=0.005 | eQTL p<0.05:  alpha=0.005 | eQTL p<0.005:  alpha=0.005 | cut off =0.5 | cut off =0.75 | cut off =0.90 | cut off =0.5 | cut off =0.75 | cut off =0.90 |
| $\lambda_{T_{c}}=$ 3.40 | 0.9404 | 0.907 | 0.8042 | 0.8724 | 0.8312 | 0.7631 | 0.5988 | 0.4537 | 0.3101 | 4.00x10^-4^ | <10^-4^ | <10^-4^ |
| $\lambda_{T_{c}}=4.09$ | 0.979 | 0.963 | 0.9253 | 0.9412 | 0.8949 | 0.8907 | 0.8093 | 0.6981 | 0.5617 | 0.0012 | <10^-4^ | <10^-4^ |
| $\lambda_{T_{c}}=4.45$ | 0.9888 | 0.9765 | 0.9607 | 0.9597 | 0.9116 | 0.9309 | 0.8836 | 0.7977 | 0.6832 | 0.002 | <10^-4^ | <10^-4^ |
| $\lambda_{T_{c}}=5.21$ | 0.9972 | 0.9868 | 0.9902 | 0.9795 | 0.9274 | 0.9646 | 0.9695 | 0.9248 | 0.8515 | 0.0075 | <10^-4^ | <10^-4^ |
| $\lambda_{T_{c}}=5.73$ | 0.9986 | 0.9884 | 0.9947 | 0.9848 | 0.9285 | 0.9694 | 0.9871 | 0.9641 | 0.9137 | 0.0138 | 3.00x10^-4^ | <10^-4^ |
