# Supplementary material for: Genetic association and transcriptome integration identify contributing genes and tissues at cystic fibrosis modifier loci
Source: PLoS Genet. 2019 Feb 26;15(2):e1008007. doi: 10.1371/journal.pgen.1008007 (PMC6407791; doi:10.1371/journal.pgen.1008007)
Supplement: S14 Table — The LD pattern at the simulated region follows that at the SLC6A14 locus. For the SS method, the nominal type 1 error was set at alpha = 0.05 or alpha = 0.005. The eQTL evidence was measured continuously as -log10 (eQTL p-value) or dichotomized using the eQTL p<0.05 or <0.005 threshold. For COLOC and eCAVIAR, the false positive rates were calculated by applying the 0.5, 0.75 or 0.9 threshold to the colocalization posterior probability. The values of λZc1 and λZc2 represent the standardized true effect sizes of two GWAS associated variants, while λTc1 and λTc2 represent standardized true effect sizes of two eQTL variants. Here, λZc1 is set to be 6.57 such that 0.8 power is achieved to detect that GWAS signal at significance level 10−8 and λZc2 is set to be 0. λTc1 is set to be 3.4, 4.09, 4.45, 5.21 or 5.73 for each row of the table such that 0.01, 0.05,0.1,0.3, or 0.5 power are achieved to detect the eQTL association at significance level of 10−8, and λTc2 is set to be 5.73 such that 0.5 power is achieved to detect that eQTL signal at significance level of 10−8. In total, 104 replications were simulated to obtain each cell of the table. See S1 Appendix for other simulation details. (DOCX) [file pgen.1008007.s035.docx]

**S14 Table. Power evaluation of the proposed Simple Sum colocalization analytical method, and the true positive rate of COLOC and eCAVIAR, under the alternative that the non-overlapped eQTL peak is lower than the GWAS peak (Alter4 in S7 Table).** The LD pattern at the simulated region follows that at the *SLC6A14* locus. For the SS method, the nominal type 1 error was set at alpha=0.05 or alpha= 0.005. The eQTL evidence was measured continuously as -log10 (eQTL p-value) or dichotomized using the eQTL p<0.05 or <0.005 threshold. For COLOC and eCAVIAR, the false positive rates were calculated by applying the 0.5, 0.75 or 0.9 threshold to the colocalization posterior probability. The values of $\lambda_{Z_{c1}}$and $\lambda_{Z_{c2}}$represent the standardized true effect sizes of two GWAS associated variants, while $\lambda_{T_{c1}}$and $\lambda_{T_{c2}}$represent standardized true effect sizes of two eQTL variants. Here, $\lambda_{Z_{c1}}$is set to be 6.57 such that 0.8 power is achieved to detect that GWAS signal at significance level 10^-8^ and $\lambda_{Z_{c2}}$is set to be 0. ${\lambda_{T}}_{c_{1}}$is set to be 3.4, 4.09, 4.45, 5.21 or 5.73 for each row of the table such that 0.01, 0.05,0.1,0.3, or 0.5 power are achieved to detect the eQTL association at significance level of 10^-8^, and $\lambda_{T_{c2}}$is set to be 5.73 such that 0.5 power is achieved to detect that eQTL signal at significance level of 10^-8^. In total, 10^4^ replications were simulated to obtain each cell of the table. See S1 Appendix for other simulation details.

| Alter4:  the non-overlapped eQTL peak is lower than the GWAS peak | Power of the proposed Simple Sum colocalization analytical method | | | | | | True positive rate of COLOC | | | True positive rate of eCAVIAR | | |
| --- | --- | --- | --- | --- | --- | --- | --- | --- | --- | --- | --- | --- |
|  | -log10(eQTL p):  alpha=0.05 | eQTL p<0.05:  alpha=0.05 | eQTL p<0.005:  alpha=0.05 | -log10(eQTL p):  alpha=0.005 | eQTL p<0.05:  alpha=0.005 | eQTL p<0.005:  alpha=0.005 | cut off =0.5 | cut off =0.75 | cut off =0.90 | cut off =0.5 | cut off =0.75 | cut off =0.90 |
| $\lambda_{T_{c1}}=$3.40 | 0.8139 | 0.8527 | 0.7434 | 0.6837 | 0.7046 | 0.6364 | 0.2438 | 0.1747 | 0.1123 | 0.0019 | 0.0019 | <10^-4^ |
| $\lambda_{T_{c1}}=4.09$ | 0.9177 | 0.9337 | 0.8848 | 0.8353 | 0.8183 | 0.8002 | 0.4118 | 0.3305 | 0.248 | 0.0081 | 0.0081 | <10^-4^ |
| $\lambda_{T_{c1}}= 4.45$ | 0.9521 | 0.9556 | 0.9321 | 0.8867 | 0.8573 | 0.8608 | 0.5068 | 0.4236 | 0.3368 | 0.0147 | 0.0147 | 0 |
| $\lambda_{T_{c1}}=5.21$ | 0.9873 | 0.9815 | 0.9795 | 0.9603 | 0.9127 | 0.9329 | 0.6916 | 0.628 | 0.5423 | 0.0424 | 0.0424 | 1.00x10^-4^ |
| $\lambda_{T_{c1}}=5.73$ | 0.9948 | 0.9858 | 0.9922 | 0.9801 | 0.9336 | 0.9587 | 0.7953 | 0.7418 | 0.6703 | 0.0696 | 0.0696 | 5.00x10^-4^ |
