# Supplementary material for: Genetic association and transcriptome integration identify contributing genes and tissues at cystic fibrosis modifier loci
Source: PLoS Genet. 2019 Feb 26;15(2):e1008007. doi: 10.1371/journal.pgen.1008007 (PMC6407791; doi:10.1371/journal.pgen.1008007)
Supplement: S17 Table — The exact region is 115248275–115448275 bp as in human genome reference assembly GRCh37. The eQTL evidence used include the -log10 transform of eQTL p value, and dichotomized eQTL p-value indicator by thresholds of eQTL p<0.05 or <0.005 for each specified gene and tissue. We focus on the analysis evaluating whether the eQTLs for SLC6A14 in lung (or human nasal epithelial; HNE) colocalize with lung-associated variants more than eQTLs for AGTR2, PLS3 and CXorf61 in lung (or HNE). The contrasting colocalization test for SLC6A14 is listed as NA since we do not contrast SLC6A14 with itself; NA in other cells means no eQTL SNP with p<0.05 or p<0.005 for that gene. The column ‘No. of eQTL SNPs’ shows the number of SNPs with eQTL p-values< 0.05 or 0.005 in the 0.1Mbp region; it refers to the number of SNPs for which eQTL p-values were available at the locus when -log10(eQTLp) is used (first 4 rows of the table). All p-values are one-sided to ensure colocalization rather than negative correlation. (DOCX) [file pgen.1008007.s038.docx]

**S17 Table. Results of Simple Sum colocalization and contrasting colocalization analyses for genes at the Chromosome X locus in the region including 0.1Mbp on either side of the lead SNP in CF human nasal epithelial and lung from GTEx.** The exact region is 115248275-115448275 bp as in human genome reference assembly GRCh37. The eQTL evidence used include the -log_10_ transform of eQTL p value, and dichotomized eQTL p-value indicator by thresholds of eQTL p<0.05 or <0.005 for each specified gene and tissue (results for cut off p<0.0005 are not provided because no eQTL SNPs left in the genes rather than AGTR2). We focus on the analysis evaluating whether the eQTLs for *SLC6A14* in lung (or human nasal epithelial; HNE) colocalize with lung-associated variants more than eQTLs for *AGTR2*, *PLS3* and *CXorf61* in lung (or HNE). The contrasting colocalization test for *SLC6A14* is listed as NA since we do not contrast *SLC6A14* with itself; NA in other cells means no eQTL SNP with p<0.05 or p<0.005 for that gene. The column ‘No. of eQTL SNPs’ shows the number of SNPs with eQTL p-values< 0.05 or 0.005 in the 0.1Mbp region; it refers to the number of SNPs for which eQTL p-values were available at the locus when -log_10_(eQTLp) is used (first 4 rows of the table). All p-values are one-sided to ensure colocalization rather than negative correlation.

| Lung eQTL evidence | eQTL genes in Lung | HNE | | | GTEx | | |
| --- | --- | --- | --- | --- | --- | --- | --- |
|  |  | No. of eQTL SNPs | Colocalization SS p-value | Contrasting Colocalization SS p-value (SLC6A14 vs. other gene) | No. of eQTL SNPs | Colocalization SS p-value | Contrasting Colocalization SS p-value (SLC6A14 vs. other gene) |
| -log10 (eQTL p) | SLC6A14 | 468 | 2.4x10^-4^ | NA | 408 | 1 | NA |
|  | AGTR2 | NA | NA | NA | 408 | 2.82x10^-7^ | 1 |
|  | PLS3 | 468 | 4.58x10^-8^ | 1 | 408 | 0.0195 | 1 |
|  | CXorf61 | 468 | 0.0014 | 0.0247 | 408 | 0.13 | 1 |
| eQTL p<0.05 | SLC6A14 | 257 | 4.45x10^-4^ | NA | 29 | 1 | NA |
|  | AGTR2 | NA | NA | NA | 214 | 1.40x10^-8^ | 1 |
|  | PLS3 | 4 | 1 | 6.93x10^-5^ | 66 | 0.341 | 1 |
|  | CXorf61 | 3 | 0.975 | 8.29x10^-4^ | 0 | 1 | 5.51x10^-5^ |
| eQTL p<0.005 | SLC6A14 | 78 | 0.991 | NA | 15 | 1 | NA |
|  | AGTR2 | NA | NA | NA | 138 | 4.86x10^-5^ | 1 |
|  | PLS3 | 0 | 1 | 1.36x10^-7^ | 2 | 1 | 4.52x10^-5^ |
|  | CXorf61 | 0 | 1 | 1.36x10^-7^ | 0 | 1 | 1.05x10^-4^ |
